# The role of socio-economic material stocks for natural resource use in the United States of America from 1870 to 2100

**DOI:** 10.1111/jiec.13166

**Published:** 2021-07-01

**Authors:** Jan Streeck, Quirin Dammerer, Dominik Wiedenhofer, Fridolin Krausmann

**Affiliations:** 1 Institute of Social Ecology, University of Natural Resources and Life Sciences, Vienna, Austria; 2 Department for Economics and Social Sciences, Institute of Social Ecology, University of Natural Resources and Life Sciences, Schottenfeldgasse 29, Vienna, 1070 Austria

**Keywords:** circular economy, economy-wide material flow analysis (EW-MFA), industrial ecology, in-use material stocks, sustainable resource management, waste management

## Abstract

Globally, more than half of all extracted materials are used to build and maintain material stocks. The United States of America (USA) is one of the largest global consumers of these materials. To assess the role of stocks for long-term material use in an affluent industrialized economy, we present an analysis of material use and stock accumulation for the USA from 1870 to 2100. On the basis of the dynamics of stocks and resulting end-of-life (EoL) outflows, we investigate stock-building material demand, waste management, and circular economy potentials and present two prospective scenarios until 2100 to highlight the long-term effect of stock-flow dynamics. From 1870 to 2017, we found continuous stock growth, which strongly decelerated after the 2007 crisis. Overall, 40% of historical domestic material consumption was used to build and maintain stocks. EoL outflows from stocks increased until 2017, about half of which were discarded as final waste. In both prospective scenarios to 2100, stock build-up and maintenance require material inputs larger than those used over the whole historical period. Stabilizing stocks near the current level could mitigate 23% of material demand compared to a return to pre-2007 stock growth. Stabilized stocks also result in stable EoL outflows after 2050, whereas ongoing stock growth makes outflows grow until 2080. Increased recycling of these EoL outflows could potentially cover for large parts of material requirements for maintaining stable future stocks. Limiting demand for stocks and increasing recycling will require targeted demand-side policies, adjustments to EoL management, and the establishment of recycling industries within the USA. This article met the requirements for a gold–gold *JIE* data openness badge described at http://jie.click/badges.

**Supplementary Information:**

The online version of this article (doi:10.1111/jiec.13166) contains supplementary material, which is available to authorized users.

## INTRODUCTION

Socio-economic material stocks of manufactured capital in infrastructure, buildings, and durable goods play a crucial role for sustainability (Lanau et al., 2019; Weisz et al., 2015). On the one hand, material stocks are required to provide services (e.g., shelter, mobility) and a minimum level is essential for meeting basic human needs (Chester, 2019; Haberl et al., 2017; Haberl et al., 2019; Thacker et al., 2019). Achieving the sustainable development goals therefore requires massive investments into new infrastructure, especially in the Global South (Thacker et al., 2019). On the other hand, material stocks lock in large and continuous inflows of material and energy required to build up, maintain, and make use of them (Krausmann et al., 2017; Krausmann et al., 2020; Pauliuk & Müller, 2014). Therefore, material stock dynamics and resulting resource use patterns contribute to socio-ecological challenges like climate change (Davis et al., 2010; Edenhofer et al., 2014; IPCC, 2018) and biodiversity loss (Maxwell et al., 2016; UNEP, 2019a). The temporal dynamics of material accumulation in stocks further play a crucial role for closing material loops in a circular economy (Haas et al., 2020). Understanding and managing material stock dynamics is thus of considerable importance for strategies to reconcile societal well-being and ecological sustainability (Haberl et al., 2019; Mayer et al., 2017; Pauliuk & Müller, 2014; Ramaswami et al., 2016).

The USA has been the dominating economy of the last century and is one of the largest consumers of natural resources and emitters of GHG emissions worldwide, both in absolute and per capita terms (Gierlinger & Krausmann, 2012; Ritchie & Roser, 2017; UNEP, 2019a, 2019b): In the last century, the USA has used 16% of all materials extracted globally and emitted 25% of all anthropogenic CO_2_ emissions. Urbanization and the build-up of large infrastructure networks has been an important driver of resource use during the transition from a biomass-based to a mineral and fossil fuel-based economy in the last century (Gierlinger & Krausmann, 2012; Miatto, Schandl, Wiedenhofer et al., 2017). Many existing US infrastructures are in poor condition and require substantial investments (BTS, 2019; Choate & Walter, 1983; Petroski, 2016). US residential housing, for example, is a major source of emissions due to large floor area, low settlement density, and often energy inefficient construction (Goldstein et al., 2020). Building-up new and more resource efficient stocks in the context of meeting climate and sustainability targets will require substantial amounts of materials in the future (e.g., Fishman & Graedel, 2019; Fishman et al., 2018). Policy and science in the USA strive for improved material management and supply reliability. In the U.S. Environmental Protection Agency Sustainable Materials Management Program, specific goals for reducing US material demand and a prioritization of material reuse and recycling are set as goals, in order to decrease related impacts and material disposal (U.S. EPA, 2015, 2020b). Additionally, concern about material criticality and supply security, especially for metals, has a long tradition in the USA (Graedel et al., 2012; U.S. DOC, 2019). The USA, therefore, is an interesting case to scrutinize stock development and resource use implications during the historical socio-metabolic transition. With this study, we provide a comprehensive knowledge base on stock-flow relations in the USA and explore possible future pathways toward more sustainable resource use.

So far, many studies on the USA focused only on material flows (Gierlinger & Krausmann, 2012; Krones, 2016), on specific stocks of products or buildings (Chen & Graedel, 2015a, 2015b; Fishman & Graedel, 2019; Fishman et al., 2018; Miatto, Schandl, Wiedenhofer et al., 2017; Reyna & Chester, 2015), the urban or regional scale (Pincetl et al., 2014; Recalde et al., 2008) or stocks of specific substances, especially metals (e.g., Chen & Graedel, 2012; Kapur et al., 2008; Müller et al., 2006). Fishman et al. (2014) produced the first estimate of the USA's total stocks for the period 1930 to 2005. They used an inflow-driven material flow model and represented stocks in aggregate material groups. Their modeling however did not explicitly discern recycling and downcycling flows, omitted important materials such as plastics, and ends in 2005. A comprehensive and detailed analysis of economy-wide stocks and flows of materials including the recent and possible future development is so far lacking.

Herein, we present a long-term (1870–2017) estimation of economy-wide material flows and stocks for the USA, using the inflow-driven MISO model (Wiedenhofer et al., 2019). We relate resource flows for stock build-up and maintenance of 13 stock types to overall resource use and assess in how far the management of end-of-life (EoL) outflows from stocks, that is, recycling, downcycling and scrap exports, can contribute to decreasing primary resource use and final waste. In two main and one supplementary prospective scenario until 2100, assuming a continuation of low stock-building material use after the 2007/2008 financial crisis, reductions in material use, and a rebound to higher pre-crisis activity, we explore future stock trajectories, stock-building material demand, as well as potential EoL outflows and implications for the management of secondary materials and primary resource demand. More specifically we aim to answer the following research questions (RQs):

- RQ1: How did material stocks develop from 1870 to 2017?
- RQ2: What role does the build-up and maintenance of material stocks play in overall domestic material consumption?
- RQ3: How have the EoL outflows from stocks evolved and how much secondary material was recovered (recycled, downcycled, or exported)?
- RQ4: Which role does stock growth and EoL outflow management play for future resource use?

## METHODOLOGY AND DATA

### System definition and model description

**FIGURE 1:**
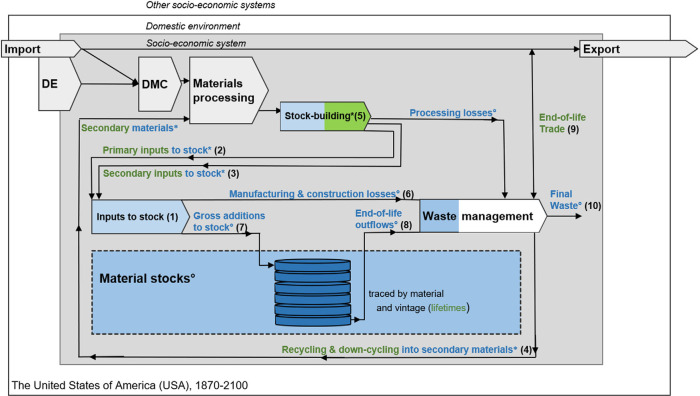
System definition of the stock-flow modeling applied to the USA from 1870 to 2100. Stock and flow labels in blue and marked with a circle (°) are endogenously calculated, while flow labels in blue and green and marked with an asterisk (*) are endogenous or exogenous depending on the data sources that were used for the respective material (see Sections 1 and 2 in Supporting Information S1). Parameter labels in green mark exogenous items. DE, domestic extraction; DMC, domestic material consumption. Figure adapted from Wiedenhofer et al. (2019)

This study employs the material inputs, stocks and outputs (MISO) model which follows an inflow-driven modeling approach (Wiedenhofer et al., 2019). The spatial boundary of the study is the USA in its current boundaries (including Alaska and Hawaii); the time frame spans from 1870 to 2100 (see Figure 1).

We use a spin-up period of 70 years from 1800–1870 to derive a stable initial value for stocks and flows in the first year of observation (1870). We distinguish 13 material stock types (see Table Table 1) which have been reported to comprise >98% of the mass of all global stocks (Krausmann et al., 2017).

**TABLE 1:** Mean lifetimes and standard deviations were sourced from literature (see Table S1.5 in the Supporting Information S1) and used in normal distributions for each of the 13 materials from 1870 to 2100

Material stocks of …	Lifetimes 1870 (years ± 1 stdv)	Lifetimes 2017 (years ± 1 stdv)
Concrete	46 ± 5	52 ± 6
Asphalt	35 ± 4	35 ± 4
Bricks (and stones)^a^	75 ± 8	75 ± 8
Aggregates	80 ± 24	80 ± 24
Solid wood	50 ± 5	63 ± 7
Paper and paperboard	3 ± 0.3	3 ± 1
Plastics	11 ± 2	10 ± 2
Iron/steel	29 ± 3	34 ± 4
Aluminum	31 ± 4	22 ± 3
Copper	39 ± 8	34 ± 7
Other metals	33 ± 5	30 ± 5
Flat glass	50 ± 5	30 ± 3
Container glass	5 ± 1	3 ± 0.3

Depending on data availability, values between 1870 and 2017 were either held constant (single lifetime datapoint), linearly interpolated between two or more datapoints, or estimated by weighting the lifetime of different stock types from literature (e.g., buildings and roads) by time series data on material use in these different stock types (see the Supporting Information S1 for detailed explanation and Figure S1.1 in Supporting Information S1 for the trajectory of lifetimes). For prospective modeling to 2100, lifetimes from 2017 were held constant.

Abbreviation: stdv, standard deviations.

^a^Bricks and stones are in the following referred to as Bricks.

The MISO-model requires data for the exogenous parameters in Table 2.

**TABLE 2:** Exogenous and endogenous parameters used in the MISO model

Exogenous	Input of stock-building materials*; primary and secondary inputs to stock*; rates for processing losses; rates for manufacturing and construction losses; lifetimes; re- and downcycling rates or flows*; trade in end-of-life materials
Endogenous	Input of stock-building materials*; primary and secondary inputs to stock*; re- and downcycling rates or flows*; gross additions to stock; end-of-life outflows from stocks; material stocks; final waste

Items marked with an asterisk (*) are either endogenous or exogenous depending on the information available for each material (see Sections 1 and 2 in Supporting Information S1).

For most materials, the inputs to stock (*its*, numbered as identifier 1 in Figure 1), consisting of primary and secondary inputs (*its_prim/sec*, identifiers 2–3), were used as exogenous model input (see Section 2 in Supporting Information S1). Inputs to stock originate from (Equation 1) statistics of primary production (*prod_prim*), end-of-life recycling (*Rcycl)*, downcycling (*Dcycl;* identifier 4; Equations 5 and 6) and net trade of materials in raw, semi-finished, and final products (*net_product_trade*; e.g., crude steel, steel tubes, and steel contained in a car).

1 $$ it{s}_m\kern0.33em (t)= its\_ pri{m}_m\kern0.33em (t)+ its\_ se{c}_m\kern0.33em (t)= prod\_ pri{m}_m(t)+\kern0.33em Rcyc{l}_m\kern0.33em (t)+\kern0.33em Dcyc{l}_m(t)+\kern0.33em net\_ product\_ trad{e}_m\kern0.33em (t). $$

Instead of inputs to stock, the input of stock-building materials (identifier 5) was partially used as exogenous model input for glass, asphalt, and bricks (see Section 2 in Supporting Information S1). Formulas for the calculation of inputs to stock from the input of stock-building materials can be found in Supporting Information S1, Section 1.

In a first model step, losses accruing during manufacturing and construction (identifier 6; e.g., losses during metal manufacturing) are subtracted from inputs to stock to obtain gross additions to stock (GAS, identifier 7; Equation 2). To calculate losses in manufacturing and construction, wastage rates were used (see Section 2 in Supporting Information S1).

2 $$ \kern0.33em \mathrm{GA}{\mathrm{S}}_m\kern0.33em (t)= it{s}_m\kern0.33em (t)\ast \left[\kern0.33em 1- manuf\_ constr\_ wastage\_ rat{e}_m(t)\right]. $$

The GAS remain in stock until they are released as EoL outflows, when reaching the end of their service lifetime (identifier 8). The stock of material *m* in year *t* is calculated from the balance of GAS, EoL outflows, and the stock from year *t* − 1 (Equation 3). The GAS are handled as own age-cohorts *c* (*c = t*). The total stock is the sum over all age-cohorts *c* of *S(c,m)*.

3 $$ {S}_{c,m}\kern0.33em (t)=\kern0.33em {S}_{c,m}\left(t-1\right)+\kern0.33em \mathrm{GA}{\mathrm{S}}_{c\kern0.33em =\kern0.33em t,m}(t)-\mathrm{Eo}{\mathrm{L}}_{c,m}(t).\kern0.33em $$

EoL outflows from stock are calculated as outflows over all stock age-cohorts *c* and modeled via cohort, material and time-specific lifetime normal distributions *L(t,t’)* (*t’* = year when stock cohort entered use phase; Equation 4). We calculated mean lifetimes as the average of different product group lifetimes (see Table 2 and Sections 1 and 2 in Supporting Information S1).

4 $$ \mathrm{EoL}\kern0.33em \mathrm{outflows}\kern0.33em \mathrm{from}\kern0.33em \mathrm{stoc}{\mathrm{k}}_{c,m}\kern0.66em (t)=\mathrm{Eo}{\mathrm{L}}_{c,m}\kern0.99em (t)=\kern0.66em {S}_{c,m}\kern0.33em (t)\ast \kern0.33em {L}_{c,m}\kern0.33em \left(t,{t}^{\prime}\right). $$

EoL outflows are either recovered, that is, recycled or downcycled domestically (identifier 4) to become secondary material inputs to stock, or exported (EoL trade, identifier 9); or become part of final waste (identifier 10). Recycling refers to using EoL outflows in the initial stock type, while downcycling only refers to the use of EoL outflows of concrete, asphalt, and bricks as secondary aggregates (e.g., for road base courses).

For those materials where absolute recycling flows were available (see Section 2 in Supporting Information S1) we directly used those as secondary inputs to stock. For all other materials, we estimated recycling flows via EoL recycling rates from the literature; as was also the case for downcycling flows of construction materials (Equations 5 and 6; *cstmin* = concrete, asphalt, bricks).

5 $$ \mathrm{Recycling}\kern0.33em \mathrm{flo}{\mathrm{w}}_m(t)\kern0.33em =\mathrm{Rcyc}{\mathrm{l}}_m\kern0.33em (t)\kern0.33em =\mathrm{Eo}{\mathrm{L}}_m\kern0.33em (t)\ast \kern0.33em \mathrm{Recycling}\kern0.33em \mathrm{rat}{\mathrm{e}}_m(t) $$

6 $$ \mathrm{Downcycling}\kern0.33em \mathrm{flow}\kern0.66em (t)=\kern0.33em \mathrm{Dcycl}\kern0.33em (t)\kern0.33em =\left[\mathrm{Eo}{\mathrm{L}}_{cstmin}\kern0.33em (t)-\mathrm{Rcyc}{\mathrm{l}}_{cstmin}(t)\right]\ast \mathrm{Downcycling}\kern0.33em \mathrm{rat}{\mathrm{e}}_{cstmin}(t) $$

Final waste (identifier 10) comprises losses from processing of stock-building materials (*pr_waste*; see the Supporting Information S1), manufacturing and construction (*mc_waste*), as well as EoL outflows less recycled, downcycled, and exported materials (Equation 7).

7 $$ \mathrm{Final}\kern0.33em \mathrm{wast}{\mathrm{e}}_m\kern0.33em (t)= pr\_ wast{e}_m(t)+ mc\_ wast{e}_m(t)+\kern0.33em \left[\mathrm{Eo}{\mathrm{L}}_m\kern0.33em (t)-\kern0.33em \mathrm{Rcyc}{\mathrm{l}}_m(t)-\kern0.33em \mathrm{Dcycl}(t)-\mathrm{Eo}{\mathrm{L}}_m\left(t,\mathrm{exported}\right)\right] $$

Due to lack of information, we do not quantify what happens to final waste, which will either be returned to the environment, accumulate in controlled landfills, be sent to other treatment facilities (e.g., incineration) or exported (if not already captured in Equation 6 export); or remain in place as hibernating stocks (e.g., abandoned rail tracks or buildings). For more detail on modeling, see Supporting Information S1 and Wiedenhofer et al. (2019).

### Data gathering and handling

Data was sourced from various national and international databases, MFA guidelines (Krausmann et al., 2018), reports (e.g., EPA, 2018; NAPA, 2019; USDT, 1993; Wilburn & Goonan, 1998) and scientific literature (e.g., Cochran & Townsend, 2010; Dammerer, 2020; Glöser et al., 2013; Ruth & Dell'Anno, 1997). A major source for data on material flows was the United States Geological Survey (Kelly & Matos, 2014) and its Mineral Commodity Summaries (USGS, 2019c), which provide time series data for the period 1900 to 2017. Data for the 19th century was taken from data compilations from the Bureau of the Census (1949, 1975) and from Gierlinger and Krausmann (2012). For some materials, data were available for the full spin-up period (i.e., from 1800 onward) or from the year when production began (e.g., 1886 for aluminum). If that was not the case, inputs to stock were calculated by multiplying constant per capita values for the earliest year available (e.g., 1869 for iron/steel) with population data for earlier years sourced from Bolt et al. (2018).

Trade data for stock-building materials and manufactured products was sourced primarily from the above mentioned sources. From 1962 onward, detailed trade data was additionally available from UNSD (2019). Material contents of iron/steel, aluminum, copper, bricks, glass, and solid wood in traded products were estimated using product-specific material intensities compiled previously (Streeck et al., 2020). Trade data from the UNSD (2019) was modified following the approach of Dittrich and Bringezu (2010) and Pauliuk et al. (2013). Detailed conduct and considered commodities can be found in Streeck et al. (2020) and Section 2.5 in Supporting Information S1.

Reported data on sand, gravel, and aggregates is of comparatively poor quality (Miatto, Schandl, Fishman et al., 2017). Therefore aggregates used in concrete and asphalt production were estimated based on bitumen and cement consumption, following standard MFA procedures (Gierlinger & Krausmann, 2012; Krausmann et al., 2018; see Section 2.3 in Supporting Information S1). Additionally, use of aggregates in sub-base and base-course layers was estimated based on GAS of concrete, bricks and asphalt, following procedures developed in previous work (Krausmann et al., 2017; Krausmann et al., 2018; Wiedenhofer et al., 2021) and using USA-specific, conservatively low factors derived from Miatto, Schandl, Wiedenhofer et al. (2017, see Section 2.3 in Supporting Information S1).

Detailed information for quantifying all exogenous parameters are provided in Section 2 in Supporting Information S1 and summarized in Tables S1.1–S1.5 in Supporting Information S1.

### Validation, uncertainty, and sensitivity

We cross-checked and validated our results against the published literature, finding good agreement of results (see Section 3 of Supporting Information S1), and additionally conducted uncertainty analysis of results via a probabilistic approach in combination with sensitivity analysis. The MISO model facilitates Monte Carlo simulation (MCS) for each input datapoint for which we used normal distributions (*n* = 1000). The uncertainty information input to MCS was based on semi-quantitative uncertainty evaluation as proposed by Laner et al. (2016). For assessing the sensitivity of the results, we varied material lifetimes by ±50%.

### Prospective assessment

To evaluate the effect of stock-flow dynamics on potential resource use and circularity, we designed two main and one supplementary scenario from 2018 to 2100 which only differ regarding the assumed future material inputs to stock. For scenario 1 “low growth continuation”, we assumed total inputs to stock on the low level that prevailed since the 2007/2008 financial crisis (in absolute terms: 2.3 Gt/year from 2018 to 2100). In scenario 2 “high growth return”, we assumed that total inputs to stock rebound to the pre-crisis level of 2006 in 2018 and then remain on that level (in absolute terms: 2.9 Gt/year from 2018 to 2100). Additionally, we provide a supplementary third scenario “additional reduction” in which we explore further reductions of material inputs to stock (−32% to 1.6 Gt/year from 2020 to 2100). The results of this supplementary scenario are shown in Section 4.3.1 in Supporting Information S1 and briefly referred to in the main text. The other exogenous parameters required to calculate material stocks and EoL outflows from stocks (manufacturing and construction wastage rates, lifetimes) were kept constant on the level of 2017 (see Table 2 and Section S2 in Supporting Information S1 for 2017 figures).

## RESULTS

Below we present our main results. For detailed information about the validation of results, their uncertainty and sensitivity, please refer to Sections 3 and 4 of Supporting Information S1.

### Inputs to stock, net additions to stock, and stock dynamics

**FIGURE 2:**
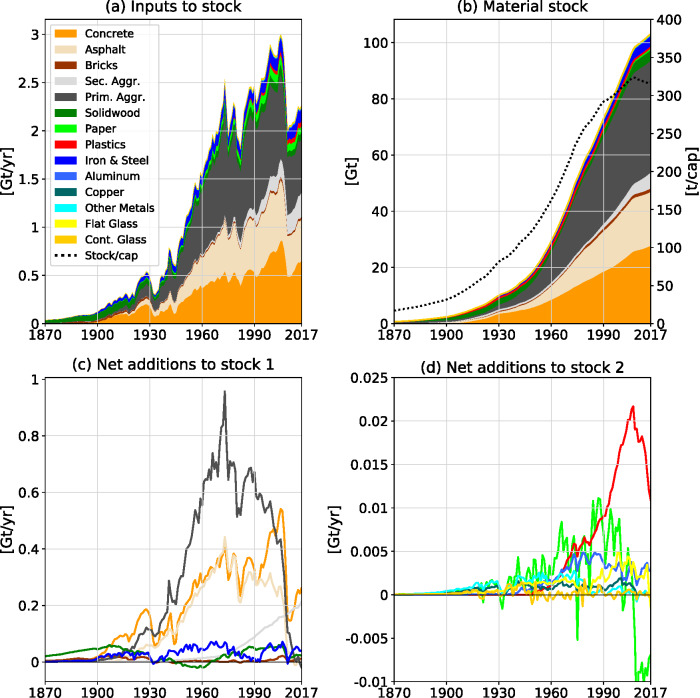
Development of (a) inputs to stock (primary and secondary inputs), (b) total and per-capita material stocks, and (c, d) net additions to stock (NAS) in the USA from 1870 to 2017. NAS equal the year-to-year changes of stocks (equals gross additions to stock minus end-of-life outflows). Population data to calculate per capita figures is from Bolt et al. (2018) and Bureau of the Census (2019b). Underlying data used to create this figure can be found in Supporting Information S2

Inputs to stock (primary and secondary) grew from 0.04 Gt/year in 1870 to 3 Gt/year in 2005, thereafter dropping to 2.3 Gt/year in 2017 (Figure 2a). Concrete, asphalt, and the aggregates in base courses and foundations of roads and buildings were quantitatively the most important materials over the entire time. Material stocks grew continuously, from 0.7 Gt in 1870 to 103 Gt in 2017, multiplying 150-fold (Figure 2b).

Only in recent years, a slow down of absolute stock growth became visible. Per capita stocks increased from 17 t/cap in 1870, peaked at 323 t/cap in 2007 and then slightly declined to 317 t/cap in 2017. In 2017, material stocks consisted primarily of aggregates in foundations of buildings and roads (45.4 Gt, 140t/cap), concrete (27.3 Gt, 84 t/cap) and asphalt (19.2 Gt, 59 t/cap), followed by wood (4.4 Gt, 13 t/cap), iron/steel (4.2 Gt, 13 t/cap), and bricks and stones (1.3 Gt, 4 t/cap). Plastics (0.6 Gt, 2 t/cap), paper and paperboard (0.24 Gt, 0.7 t/cap), aluminum (0.2 Gt, 0.6 t/cap), glass (0.19 Gt, 0.6 t/cap), copper (0.09 Gt, 0.3 t/cap), and other metals (0.13 Gt, 0.4 t/cap) were least present.

Net additions to stock (NAS) reflect the year to year change of stocks. Also, for NAS the dominance of construction minerals (aggregates, concrete, asphalt, bricks) shows (Figure 2c,d). NAS of aggregates peaked in 1973, those of concrete in 2005. After 2007 we observed a strong decline of NAS and thus stock growth (between 2006 and 2009 NAS dropped by 68%). For paper and paperboard, NAS were even negative at an average −8 Mt/year since 2007, indicating a declining stock.

### EoL outflows and their fate (RQ3)

**FIGURE 3:**
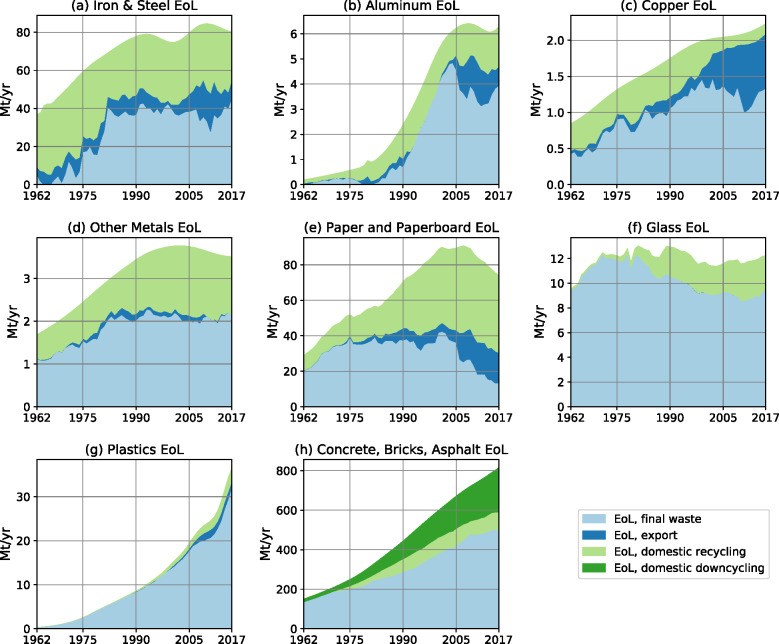
Fate of end-of-life (EoL) outflows from stocks for stock-building materials in the USA 1962–2017. EoL outflows are either recovered (recycled, downcycled, exported) or end up as final waste for further treatment. Final waste is calculated by subtracting recycled, downcycled, and exported material from total EoL outflows. Sources: total EoL outflows are a model output and therefore do not reflect year-to-year variations, while the amount that is recycled and downcycled is either taken from statistics (Kelly & Matos, 2014 and USGS, 2019d) or calculated via recycling and downcycling rates (see Section 2 in Supporting Information S1). EoL exports are from Gorman and Dzombak (2020), Resource Recycling Inc. (2019), UNSD (2019), USGS (2015). Note for iron and steel: the share of EoL scrap on total scrap recycling was estimated with old scrap shares for 1996–2017 (USGS, 2019d); before 1996 old scrap shares were kept constant at the level of 1996. For detailed information please see Section 2 in Supporting Information S1. Underlying data used to create this figure can be found in Supporting Information S2

At the end of their lifetimes, materials accumulated in stocks turn into EoL outflows. Modeled EoL outflows increased from 0.005 Gt/year in 1870, to 0.4 Gt/year in 1962 and further to 1.7 Gt/year in 2017 (see Figure S1.40 in Supporting Information S1). These outflows are then either recovered, that is, recycled or downcycled domestically or exported; or disposed of as final waste. For the investigation of EoL management, we focus on the period from 1962 onward, since appropriate data on scrap exports were not available for earlier years. For all materials, EoL outflows increased markedly from 1962 until the early 2000s (Figure 3). Since then, EoL flows of iron and steel, aluminum and glass stabilized at a high level, while for paper and other metals, they started to decline. For copper, plastics, and construction materials (asphalt, bricks, and concrete), we find a continuous increase of EoL outflows until 2017. In 2017, EoL construction materials (concrete, bricks, and asphalt) amounted to 815 Mt/year, about four times larger than the EoL outflows of all other materials combined (215 Mt/year).

Through recycling, downcycling, and scrap exports, 37% of all EoL outflows were recovered from 1962 to 2017. From 1962 to 2017, absolute recycling and downcycling increased for all materials except for iron and steel and copper. For iron and steel, recycling remained rather stable at an average 33 Mt/year, while for copper, recycling increased to 0.6 Mt/year in 1980 but then declined to ∼0.15 Mt/year in 2017.

This development reflects a decline of domestic copper industry in the USA which shifts to other countries with low-cost EoL processing (Gorman & Dzombak, 2020). Instead of domestic treatment, recycling was increasingly happening on an international scale, indicated by the shift from domestic recycling toward scrap exports. Of all recovered iron, aluminum, copper and paper in 2017, 71 Mt/year were recycled domestically and 28 Mt/year were exported to be recycled or treated elsewhere in the world, most notably in China and Turkey (Chen, 2013; Nathan Associates, 2004; U.S. ITC, 2020; USGS, 2019a, 2019b). However, Gorman and Dzombak (2020) note that for copper there seems to be a decreasing trend of scrap exports around and after 2010. The authors suggest that this might be related to changes in import policies of China, as also applicable to other materials, for example, plastics waste (Brooks et al., 2018). Also for iron and steel and aluminum scrap net exports declined since 2011 (Figure 3). Gorman and Dzombak (2020) argue that because of restrictions for scrap exports, the USA might need to increasingly manage EoL scrap domestically in the future.

Despite the recovery of EoL outflows through recycling, downcycling, or exports, 22 Gt of materials ended up as final waste (63% of EoL outflows) since 1962. Plastics were the material type where the highest share of EoL outflows ended up as final waste (91%), iron and steel showed the lowest share (40%). Final waste flows were growing for all materials in the first two decades after 1962. In the last two decades, however, final waste flows of many materials stabilized (iron and steel, copper, other metals) while massive increases of waste flows were observed for plastics and construction materials. The only materials showing a recently decreasing trend of final waste flows are paper (since 2001, in 2017: 18% of EoL outflows turning into final waste), glass (since 1978, in 2017: 77% of EoL outflows), and aluminum (since 2004, in 2017: 63% of EoL outflows). Except for these materials and despite efforts to increase material recovery (U.S. EPA, 2015, 2020a), no trend toward a reduction of final waste can be observed.

### Cumulative fate of stock-building material flows

**FIGURE 4:**
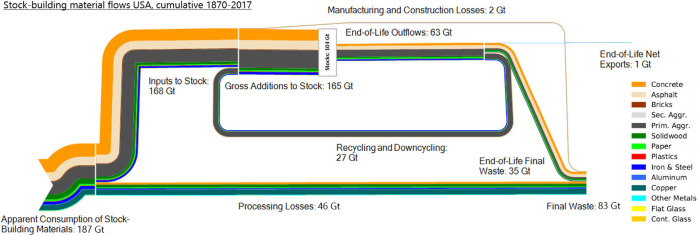
Sankey diagram of cumulative stock-building material flows from 1870 to 2017. Mass balance in figures might not fully match due to rounding (see Supporting Information S2 for exact figures). Apparent consumption of stock-building materials comprises primary raw materials used in production and net imports of raw, semi-finished, and final products. Gross additions to stock (GAS) contain all materials that actually accumulate in stocks. End-of-life (EoL) outflows are materials discarded from stocks that reached the end of their service lifetime. GAS minus EoL yield the cumulative net additions to stock which equal the stock in 2017 (additional small initial stock in 1870). End-of-life net-export denotes the export of scrap recovered from EoL outflows; note that data on scrap export was mostly only available from 1962 onward (except for iron and steel, aluminum; see caption of Figure 3 for data sources). Underlying data used to create this figure can be found in Supporting Information S2

Figure 4 shows the cumulative fate of stock-building materials in the USA from 1870 to 2017. During these 147 years, a total of 187 Gt of stock-building materials were processed, of which 139 Gt were used to build up and maintain stocks, while 26% turned into processing losses (46 Gt) or manufacturing and construction loss (2 Gt). The largest share of stock-building materials were non-metallic minerals (127 Gt, 67%) followed by metals and ores (33 Gt, 18%), biomass (25 Gt, 13%) and fossil energy carriers used to produce bitumen and plastics (3 Gt, 2%). Together with 27 Gt of secondary materials from recycling and downcycling of EoL outflows, a cumulative 165 Gt was added to stock as gross addition to stock (GAS).

Of the cumulative inflow of GAS, a total of 102 Gt (62%) was still in use in stocks in 2017 (additional small initial stock in 1870 = 103 Gt stock in 2017). Besides recycling and downcycling, the 63 Gt total EoL outflows were reduced by ∼1 Gt of exported scrap, leaving 35 Gt of EoL final waste. The recovery rate of EoL outflows (recycling, downcycling, export) over the whole period was 44%, corresponding to 17% of all inputs to stock. Cumulative final waste from losses in processing, manufacturing, construction, and from EoL outflows was 83 Gt.

### Material stock dynamics and resource use from 2018 to 2100

The trajectory of material stock growth will substantially shape future resource use. Following the drop of inputs to stock after 2007 (Figure 2a), the high level of affluence in the USA and the post COVID-19 economic recovery and potentially Green New Deal stimulus, one might hypothesize an upcoming stabilization of stocks and/or flows at some level (Bleischwitz et al., 2018; Lanau et al., 2019; Müller et al., 2011). To explore what such a stabilization could mean for material stocks and flows, we modeled two prospective scenarios from 2018 to 2100, in which we assume a continuation of inputs to stock on the low level of 2017, versus a rebound to pre-2007 crisis levels (see Section 2.4). Additionally, we provide a third scenario exploring an even further reduction of inputs from 2020 onward (see “additional reduction” scenario in Section 4.3.1 in Supporting Information S1).

**FIGURE 5:**
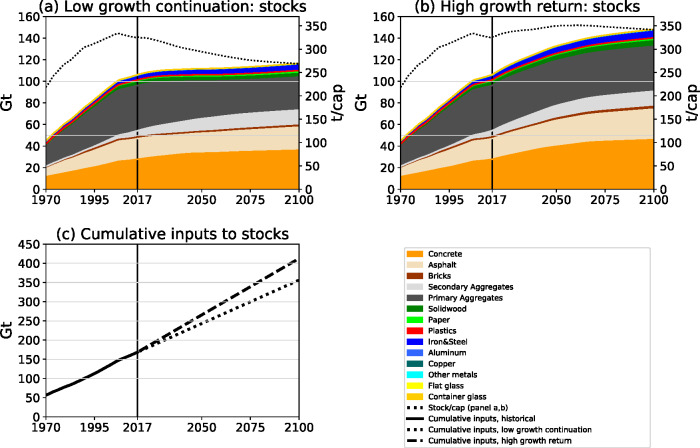
Material stock levels (absolute and per capita) for the two main prospective scenarios: (a) Scenario 1: “low growth continuation,” (b) Scenario 2: “high growth return,” and (c) cumulative material inputs to stock from 1970 to 2100 for the historical period and both scenarios. Prospective modeling starts from the year 2018 onward, values from 1970 to 2017 are historical trends, shown for comparison. Per capita values were calculated based on the medium population projections by UN DESA Population Division (2019). Underlying data used to create this figure can be found in Supporting Information S2

In the “low growth continuation” scenario, stocks increase only slowly at 0.1% per year until 2100, and reach 117 Gt or 10% larger stocks than in 2017 (Figure 5). This stock growth is lower than population growth in the medium variant forecast for the USA (UN DESA Population Division, 2019), which results in a decline in per capita stocks by 10% in 2050 (295 t/cap) and by 18% in 2100 (269 t/cap). In contrast, in the “high growth return” scenario, total stocks increase by 0.3% per year and reach 148 Gt in 2100, a 40% increase over 2017. In this scenario, stocks per capita effectively stabilize around mid-century, with 350 t/cap in 2050 and 342 t/cap by 2100.

The required stock-building material quantities in both scenarios are larger than material use in the historical period. Historical cumulative material use for stock-building amounted to 166 Gt between 1900 and 2017 (Figure 5c); from 2018 until 2100, the two scenarios result in cumulative material inputs to stock of 188 and 244 Gt. The “low growth continuation” scenario, in which stocks stabilize close to the 2017 level, shows a 56 Gt lower cumulative inputs to stock compared to the “high growth return” scenario. Limiting further stock growth might thus be a lever for curbing future material demand.

### Stock maintenance and expansion versus end-of-life outflows and recycling from 2018 to 2100

Here we explore the prospective dynamics of the materials required to expand and maintain material stocks, versus the EoL outflows potentially available for recovery, which also yields insights into the future demand for primary materials. To indicate the potential and required expansion of EoL management and increased recycling capacities, we provide a “current-recycling” baseline by keeping recycling and downcycling flows constant at their 2017 levels.

For the two prospective scenarios, the dynamics and magnitude of EoL outflows only begin to differ markedly after 2040, when stocks built after 2017 increasingly reach the end of their lifetime (see Figure S1.44 in Supporting Information S1). In the low growth scenario, total EoL outflows grow until 2052 and then stabilize at 2.1 Gt/year. In the high growth scenario, EoL outflows stabilize ∼30 years later and at a 29% higher level of ∼2.7 Gt/year. Thus, a return to stock growth from before the 2007/2008 financial crisis would result in substantially higher EoL outflows and a later peak.

**FIGURE 6:**
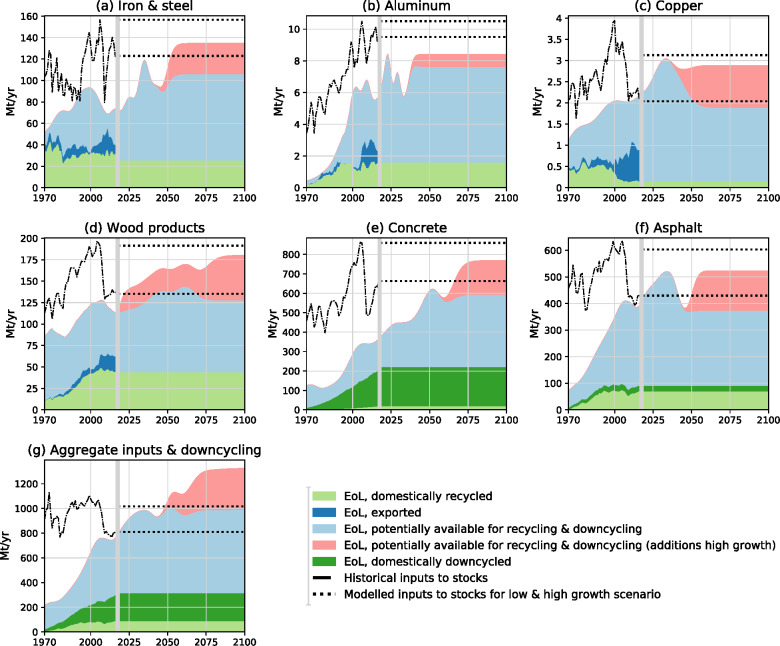
Historical inputs to stock and end-of-life (EoL) outflows from 1970 to 2017 and assumed inputs for 2018–2100 under the scenario conditions. For Scenario 1: “low growth continuation,” all parameters were kept constant on 2017 levels, for Scenario 2: “high growth return,” inputs to stock were assumed to return to the level of 2006 (i.e., before the 2007/2008 financial crisis). The figure shows which part of EoL outflows was recycled (light green), exported (dark blue), downcycled (dark green), and remained as outflows of final waste (light blue) for 1970–2017. For 2018–2100 the assessment is prospective. The pink area reflects the additional EoL outflows in Scenario 2: “high growth return” compared to Scenario 1: “low growth continuation.” Underlying data used to create this figure can be found in Supporting Information S2

For some materials, we find that in the "low growth continuation" scenario substantially improved recovery of EoL outflows could, by sheer numbers, cover nearly all required material inputs to stock (Figure 6). An example is copper, for which modeled EoL outflows between 2018 and 2050 (light blue shades) exceed the required inputs to stock (lower dotted line) and could thus theoretically allow for closing the loop (Figure 6c). However, actually achieving increased recovery and recycling of EoL outflows in the future is a substantial challenge and perfect loop closing with 100% recycling is practically impossible and hard to argue theoretically (Allwood et al., 2011; Ayres, 1999; Cullen, 2017). Recycling limitations apply, for example, for metals, due to copper contamination of iron and steel or aluminum scrap (Cooper et al., 2020; Zhu & Cooper, 2019) or the physical complexity of modern technology (Graedel et al., 2011; Graedel et al., 2015); but also for construction and demolition waste (Zhang et al., 2020).

Similarly, we find that the demand for aggregates from 2019 to 2100 could quantitatively be covered by recycling of aggregates and downcycling of concrete, asphalt, and bricks (Figure 6g) which could mitigate rising demand for natural sand (Ioannidou et al., 2020), but might have logistical (cost) constraints due to high volumes and low value. For shorter periods of time, EoL outflows of wood products and asphalt also match the required material inputs (Figure 6d,f).

For iron and steel, aluminum, and concrete, we find a gap between EoL outflows and required inputs to stock. However, also for these materials, large parts (>60–73% from 2030 onward, >78–84% from 2050) of inputs to stock could in theory be supplied by recovery of EoL material (Figure 6a,b,e). Thus, overall EoL outflows show a large potential for closing resource loops. An increase in the utilization of EoL outflows as secondary resources would, however, require radically increased recycling and downcycling, compared to the 2017 level (Figure 6).

## DISCUSSION

### Historical stock dynamics in the context of the socio-metabolic transition (RQ 1 and 2)

**FIGURE 7:**
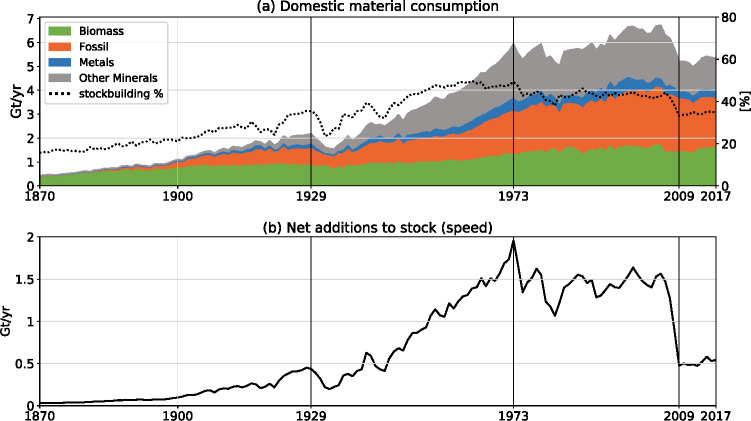
(a) Domestic material consumption (DMC) by four material groups and the share of primary stock-building materials (including net import of materials in raw, semi-finished, and final products, see Supporting Information S1) in DMC and (b) total net additions to stock (NAS) which mirror the year-to-year change of stocks and can be understood as measuring the “speed” of stock growth (Fishman et al., 2016). Stock-building materials (identifier 5 in Figure 1) include all (raw) materials which are used to manufacture material stocks which remain in the socio-economic system for longer than 1 year on average. The remainder are materials used to provide food, feed, and energy (most of biomass and fossil energy carriers) and for other dissipative uses (see Krausmann et al., 2017). Sources: data on DMC for biomass and fossil energy carriers for 1870–2005 is from Gierlinger and Krausmann (2012) and for 2006–2017 from UNEP (2019a); data for metallic and non-metallic minerals from own calculations (see Supporting Information S1). Underlying data used to create this figure can be found in Supporting Information S2

From 1870 to 2017, the USA underwent a socio-metabolic transition from a largely biomass-based economy to a fossil-fuelled society with a much higher level of material and energy use, dominated by minerals, metals, and fossil fuels (Figure 7). Gierlinger and Krausmann (2012) distinguished between coal-based (1870–1929) and oil-based (1932–1973) phases in this transition, followed by a relative stabilization of the industrial metabolic regime in the decades thereafter. Using the annual NAS as indicator of the “speed” of stock growth (Fishman et al., 2016), we find that the coal-based phase of the metabolic transition (1870–1929) can be characterized as the take-off phase into sustained stock growth in the USA. These decades were characterized by a first wave of industrialization (Merchant, 2007), electrification (Hughes, 1983; Jones, 2014), and urbanization, with urban population growing from 10 to 70 million between 1870 and 1929^1^, the opening-up of the continent by the expansion of the railroad network (which grew from 85,000 to 400,000 km in the same period, Mitchell, 2007) and the development of a large heavy industry sector (Gierlinger & Krausmann, 2012). These and other factors contributed to an accelerated growth of stocks, which increased 14-fold in this period. As a consequence, the share of stock-building materials in domestic material consumption increased markedly, from 16% in 1870 to 35% in 1929.

The Great Depression following the stock market crash in 1929 interrupted this take-off (Figure 7), and was only overcome in the late 1930s through the economic recovery shaped through the New Deal. Part of the New Deal program was a nationwide surge of infrastructure projects, heralding a new phase of accelerated stock growth and accumulating the physical underpinning of the oil-based phase of the metabolic transition. While WWII led to a short but strong reduction of the speed of stock growth, stock expansion again increased steeply until its peak during the first oil price shock in 1973. During this time, speed of stock growth (NAS) increased from 440 Mt/year in 1929 to 1,960 Mt/year in 1973 and total domestic material consumption grew substantially due to the rapid increase of the share of stock-building materials to 49% of DMC in 1973 (Figure 7a). The fast build-up of stocks was partially triggered by public spending, for example, through the Public Works Program (1935–1944) and the Federal Aid Highway Act of 1956. Both initiated large infrastructure projects, ranging from building hydropower dams, the establishment of a national electricity grid, to the expansion and upgrade of the road network, including the establishment of the US interstate highway system (Adams, 2007; Miatto, Schandl, Wiedenhofer et al., 2017; Sullivan, 2006; Weingroff, 1996; Wells & Cronon, 2012). During this period a new pattern of mass production and consumption, the “American Way of Life” emerged (Gierlinger & Krausmann, 2012) and materialized into suburbanization, mass-motorization and car-related infrastructure buildout. The number of residential housing, for example, increased from 37 million in 1940 to 69 million in 1970 (Bureau of the Census, 2012) and the number of motorized vehicles from 32 million in 1940 to 126 million in 1973 (Federal Highway Administration, 2018).

The rapid growth of NAS came to an abrupt halt in 1973, coinciding with the so-called first oil price shock and falling in a period of crisis in US energy policy and of the Fordist development model. NAS dropped and remained rather stable in the following decades, fluctuating around 1.4 Gt/year (Figure 7b). Also, the share of stock-building materials stabilized at around 43%. In this period, for which Gierlinger and Krausmann (2012) discuss a consolidation of the industrial socio-economic metabolism, the size of material stock, both in absolute terms and also per capita, continued to expand, but in much “steadier,” linear fashion. While quantitative expansion of stocks of buildings and infrastructures slowed down, qualitative changes still occurred and in combination resulted in steady growth of the stock mass. For example, while the road system was hardly expanded in terms of mileage after the 1970s, roads were upgraded to heavier and wider road types to cope with rising speed and loads of vehicles (Miatto, Schandl, Wiedenhofer et al., 2017). Also the size of buildings increased: The average newly constructed single-family house grew from 154 m² in 1973 to 222 m² in 2010 (calculated based on Bureau of the Census, 2019a); commercial buildings increased from an average of 1094 m² in the 1950s to 1755 m² for constructions around 2010 (calculated based on EIA, 2012).

The largest reductions in the speed of stock growth in the observed period occurred, when the US housing bubble burst, investments into housing collapsed and the subprime mortgage crisis kick-started the (ultimately global) financial crisis of 2007/2008 (Felton & Reinhart, 2008, 2011). Between 2006 and 2009, NAS plummeted by 68% to the level observed last in 1943 (0.5 Gt/year) and remained at that low level until 2017 (Figure 7b). Inputs to stock decreased particularly for construction materials such as concrete, bricks, solid wood, and iron and steel which dropped by 43–55% and only rose slowly to 2017 (Figure 3a). This stands in contrast to development reported for the United Kingdom, where NAS and inputs to stock bounced back and almost reached pre-crisis levels around 2014 (Streeck et al., 2020). For the USA, we find stable but low speed of stock growth from 2007 onward. In this phase, the rate of stock expansion fell below the growth rate of the population and per capita stock for the first time showed a declining trend. It has to be noted, however, that we may underestimate NAS in our modeling post 2007/2008, because demolition and lifetimes of stocks might be affected by the economic crisis, an effect we could not capture in our approach due to data constraints. If aging stocks were kept in use beyond the assumed average lifetimes (e.g., due to postponed investments), this would reduce EoL outflows and consequently result in higher NAS. Indeed, many aging infrastructures in the USA suffer from a chronic lack of investment (BTS, 2019; Choate & Walter, 1983; Petroski, 2016). While such an effect may have played a certain role, we do not expect it to fundamentally change the pattern of relative stabilization that we find for the last decade, because the reported annual inputs to stock also dropped substantially, showing much lower stock-building and maintenance activity (see Figure 3a).

### How can the USA reduce resource demand of material stocks in the future? (RQ4)

Our calculations show that at the beginning of the 21st century the USA is characterized by a high level of per capita material stocks (2017: 317 t/cap), also when compared to other high income countries (e.g., Austria, 2009: 244 t/cap, Daxbeck et al., 2009; United Kingdom, 2017: 272 t/cap, Streeck et al., 2020). This is reflected, for example, in a high level of available floor area per person (with 68 m²/capita double compared to United Kingdom), an extensive road network and a heavy vehicle fleet with >50% of vehicles being SUVs and light trucks in 2019 (Miatto, Schandl, Wiedenhofer et al., 2017; UNEP-IRP, 2019). Our results further underline the close relation between stocks and flows of materials. Whether stocks continue to grow or rather stabilize will have significant impact on the future demand for stock-building materials, waste flows, and the potential for material loop closing (Haas et al., 2020).

The prospective scenarios show that, if the low-level stock-building activity observed since the 2007/2008 financial crisis can be continued, this might lead to a stabilization of absolute material stocks close to the current level (see Section 3.4, Figure 6). In contrast, a rebound to the pre-2007 level of inputs to stock makes stocks grow from 103 Gt in 2017 to 148 Gt in 2100. Inversely, this relationship suggests that stabilizing absolute stock levels could reduce cumulative material demand by 56 Gt in the next 83 years. A further reduction of inputs similar to that observed in 2007, could save even more resources (116 Gt to 2100) but would also lead to decreasing stock levels, stabilizing at ∼83 Gt from 2070 onward—unless lifetimes of stocks can be increased (“additional reduction” scenario, see Section 4.3.1 in Supporting Information S1).

Stabilizing stock levels, or at least curbing further expansion, could be supported through measures such as more intensive use (e.g., via product sharing; Pauliuk et al., 2020), more material efficient design or downsizing (e.g., multi-family instead of single-family houses and smaller cars; Berrill et al., 2021; UNEP, 2019a). Larger-scale potentials also exist from maintaining appropriately dense, functionally mixed, and well-connected infrastructure and settlements, while re-densifying and transforming low-density car-dependent suburbs (Creutzig et al., 2015; Seto et al., 2016). Achieving such changes, however, will require targeted policies and stringent standards.

Importantly, our scenarios show that even when stocks stabilize at the current level, required material inputs for maintenance and replacements to 2100 (cumulative 188 Gt) will still be larger than historical inputs (cumulative 166 Gt since 1900; Section 3.4). A strategy to further reduce stock-related material demand while maintaining stock levels is extending stock lifetimes via targeted renovation, repair and long-lasting design instead of replacement (Pauliuk et al., 2020). Buildings in the USA, for example, have a much shorter lifetime than in Europe, which might indicate room for improvement (Hertwich et al., 2019). However, resource savings of lifetime extension have to be weighed against the operational efficiency of new versus old stocks (Hertwich et al., 2019).

Sooner or later, stocks inevitably reach the end of their lifetime, resulting in high volume EoL outflows and waste. Recycling these outflows and closing material loops can reduce primary resource extraction and related impacts. Our results suggest theoretical potential to cover from 60% up to >100% of required inputs to stock for many materials from 2030 to 2100 (see Section 3.5). Even though thermodynamic, technical, and logistical limitations impede perfect recycling (Allwood et al., 2011; Ayres, 1999; Cullen, 2017) and even though recycling and circularity rebounds might not result in a 1:1 replacement of primary production with secondary materials (Zink et al., 2018; Zink & Geyer, 2017), these potentials are considerable and warrant targeted efforts in closing loops.

An important issue in this context is the geographical scale at which recycling and loop closing are attempted. We find that in the past, recycling increasingly shifted from domestic recycling within the USA to the global economy. The resource impact of this shift is not well understood and needs to be monitored and assessed (Graedel et al., 2019; Wiedenhofer et al., 2020). The recent restrictions on scrap imports of China (e.g., plastics, copper; Di et al., 2021; Gorman & Dzombak, 2020), and the need to deal with large flows of bulk construction and demolition waste, might point toward domestic recycling becoming more important for managing EoL outflows in the future. In that regard, the ongoing de-industrialization of the USA raises the question whether the country has the capacity to deal with the rising amounts of EoL outflows domestically. We find, that for some materials, EoL outflows already now are much larger than the domestic production of these materials (e.g., aluminum and copper with 6.3 and 2.2 Mt/year EoL outflows and a production of 4.4 and 1.2 Mt/year, respectively, in 2017; Gorman & Dzombak, 2020; Kelly & Matos, 2014). Therefore, if EoL outflows should be increasingly used as secondary materials to cover inputs to stock, the capacities for domestic recycling and materials management needs to increase rapidly.

Currently, efforts toward a more sustainable materials management system in the USA are ongoing (U.S. EPA, 2020b). The specific pathway of US resource use will at least in parts be decided by the measures of the post COVID-19 recovery stimulus and a potential Green New Deal (Andrijevic et al., 2020). At the moment, any of the trajectories presented in the prospective scenarios is imaginable, ranging from material intensive large-scale investment programs like in the past, to more targeted, sustainability-oriented and circular economy efforts.

## CONCLUSIONS

The emergence of modern industrial society in the USA was underpinned by an accelerated accumulation of material stocks which provide the physical basis of production and consumption and ultimately the American Way of Life. Although the speed of stock growth slowed down since the 1970s and even more after the financial crisis of 2007/2008, stocks continue to increase in the USA. In the last one and a half centuries, 40% of the domestic material consumption in the USA were used to build and maintain stocks. In 2017, 103 Gt or 317 t/cap of material stocks were accumulated, constituting a socio-metabolic legacy for the future.

Our prospective scenarios show that a stabilization of stocks close to current levels could mitigate future resource demand, but will still require large amounts of materials for stock maintenance. Furthermore, stock dynamics not only are a major driver of material demand; with a considerable time lag due to their long lifetime, they also drive waste flows and impact the potentials for material loop closing. In addition to the 35 Gt of EoL waste from discarded stocks which accrued in the last one and a half centuries, large outflows of materials from stocks can be expected in the coming decades, predominantly from already existing stocks. While outflows could be reduced to some extent by extending stock lifetimes, they are also a source for recovering secondary materials. Our scenarios show, that when a stabilization of stocks close to the current level is achieved, the resulting outflows match or even exceed the required material inputs to maintain stocks of many materials and that there are large potentials for loop closing. To achieve this, major efforts are necessary to improve recycling. Currently, however, the de-industrializing USA has only limited capacity to recycle domestically and increasingly EoL material is exported for recycling or final treatment.

To tackle unsustainable resource use in the USA, the efforts for logistical and technical improvements via recycling and reuse must be combined with strategies to limit demand for additional material stock buildup, both of which need to be incentivized via immediate and effective policies.

## Supplementary Information

**Supporting Information S1**: This supporting information S1 provides details regarding modelling steps and data sources (Sections 1 and 2), as well as additional information on results and their uncertainty, sensitivity, and comparison to literature (Sections 3 and 4). (PDF 2.13 MB)

**Supporting Information S2**: This supporting information S2 provides data for the figures in the main article as well as results from the sensitivity analysis for main results. (XLSX 596 KB)

## Footnotes

1 Urban pop grew sevenfold in this period, calculated from data on urbanization rate and population (Our World in Data, 2020a, 2020b).
