# Supplementary material for: The role of socio-economic material stocks for natural resource use in the United States of America from 1870 to 2100
Source: J Ind Ecol. 2021 Jul 1;25(6):1486–502. doi: 10.1111/jiec.13166 (PMC13070052; doi:10.1111/jiec.13166)
Supplement: Supplementary file 1 — Supporting Information S1: This supporting information S1 provides details regarding modelling steps and data sources (Sections 1 and 2), as well as additional information on results and their uncertainty, sensitivity, and comparison to literature (Sections 3 and 4). (PDF 2.13 MB) [file 44498_2021_2506008_MOESM1_ESM.pdf]

SUPPORTING INFORMATION FOR:

Streeck, J., Q. Dammerer, D. Wiedenhofer, and F. Krausmann. 2021. The role of socio-economic material stocks for natural resource use in the United States of America from 1870 to 2100. *Journal of Industrial Ecology.*

Summary

This supporting information S1 provides details regarding modelling steps and data sources (Sections 1 and 2), as well as additional information on results and their uncertainty, sensitivity, and comparison to literature (Sections 3 and 4).

1 General Notes on Use of the MISO-model 4
2 Data for Material Flows and Parameters 7
2.1 Biomass 7
2.2 Metals 9
2.3 Non-Metallic Minerals 15
2.4 Fossil Energy Carriers 22
2.5 Additional details on trade data 23
3 Comparison with Other Studies, Uncertainty & Sensitivity 24
3.1 Biomass Comparison 25
3.2 Metals Comparison 25
3.3 Non-Metallic Minerals Comparison 28
3.4 Fossil Energy Carriers Comparison 29
4 Figures and Tables 30
4.1 Data for Material Flows and Parameters 30
4.2 Comparison with Results of other Studies 40
4.3 Additional Figures, Tables and Results 55
References 61

List of Figures

Figure S1.1: Lifetimes used in the MISO-model, 1800-2017 (for sources see Table S1.5) 35
Figure S1.2: End-of-Life recycling rates used in the MISO-model, 1800-2017 35
Figure S1.3: End-of-Life recycling flows used in the MISO-model, 1800-2017 (for sources Table S1.4) 36
Figure S1.4: End-of-Life downcycling rates used in the MISO-model, 1800-2017 (for sources Table S1.4) 36
Figure S1.5: Total road kilometres for asphaltic road types in the USA, 1905-2015 37
Figure S1.6: Shares of road types in total new asphaltic road construction in the USA, 1905-1940 38
Figure S1.7: Shares of road types in total new asphaltic road construction in the USA, 1941-1980 38
Figure S1.8: Calculated average MultiplierTotal for asphaltic roads, 1870-2017 39
Figure S1.9: Assumed share of asphalt used for new road construction in the USA, 1870-2017 39
Figure S1.10: Multipliers for Sub-Base and Base-Course Layers 1800-2017 40
Figure S1.11: Comparison of biomass stock estimates for USA for 1930-2005 40
Figure S1.12: Development of per capita stocks for iron/steel in the USA, 1900-2005 according our results 41
Figure S1.13: Comparison of per capita stock estimates for iron/steel in the USA for 1900-2008 41
Figure S1.14: Comparison of stock estimates for iron/steel in the USA for 1930-2005 42
Figure S1.15: Comparison of per capita stock estimates for aluminum in the USA for 1950-2008 42
Figure S1.16: Own results for aluminum stocks in the USA, 1900-2010 43
Figure S1.17: Comparison of estimates of stocks of non-ferrous metals in the USA for 1930-2005 43
Figure S1.18: Comparison of estimates of concrete stocks in the USA for 1900-2005 44
Figure S1.19: Comparison of estimates of per capita stocks of concrete in the USA for 1950-2014 44
Figure S1.20: Comparison of estimates of stocks of aggregates in sub-base and base-course layers in the USA for 1905-2015 (please watch out for different axis). 45
Figure S1.21: Comparison of estimates of stock of asphalt in the USA for 1905-2015 (please watch out for different axis). 45
Figure S1.22: Comparison of estimates of stocks of non-metallic minerals in the USA for 1905-2015 46
Figure S1.23: Comparison of estimates of End-of-Life outflows of paper and paperboard in the USA for 1905-2015 47
Figure S1.24: Comparison of estimates of End-of-Life outflows of solidwood in the USA for 2012-2015 48
Figure S1.25: Own results for End-of-Life outflows of aluminum in the USA, 1900-2009 48
Figure S1.26: Comparison of estimates of End-of-Life outflows of concrete in the USA for 2012-2015 49
Figure S1.27: Comparison of estimates of End-of-Life outflows of bricks and stones in the USA for 2012-2015 49
Figure S1.28: Comparison of estimates of End-of-Life outflows of glass in the USA for 1960-2015 50
Figure S1.29: Comparison of estimates of End-of-Life outflows of plastics in the USA for 1960-2015 50
Figure S1.30: Comparison of estimates of final waste flows of biomass in the USA for 1930-2005 51
Figure S1.31: Comparison of estimates of final waste flows of iron/steel in the USA for 1930-2005 .. 51
Figure S1.32: Comparison of estimates of final waste flows of non-ferrous metals in the USA for 1930-2005 52
Figure S1.33: Comparison of estimates of final waste flows of non-metallic minerals in the USA for 1930-2005 52
Figure S1.34: Comparison of estimates of paper and paperboard recycling rates in the USA for 1960-2005 53

Figure S1.35: Comparison of estimates of glass recycling flows in the USA for 1960-2005 53
Figure S1.36: Comparison of estimates of plastics recycling flows in the USA for 1980-2015 54
Figure S1.37: Comparison of sand and gravel consumption in the USA 1870-2005 reported by Gierlinger and Krausmann 2012 and replicated figures.. 55
Figure S1.38: Difference of net additions to stock for non-metallic minerals between Fishman et al. 2014 and own results in the USA for 1930-2005 55
Figure S1.39: Sensitivity analysis - material stock level results at different lifetime assumptions. (a) Mean lifetimes reduced by 50%, (b) mean lifetimes (see main paper Table 2) and (c) mean lifetimes extended by 50%. 56
Figure S1.40: Sensitivity analysis – End-of-life outflows from stocks at different lifetime assumptions. (a) Mean lifetimes reduced by 50%, (b) mean lifetimes (see main paper Table 2) and (c) mean lifetimes extended by 50%. 56
Figure S1.41: Sensitivity analysis - End-of-Life (EoL) outflows from stocks by treatment type for stock-building materials in the USA 1962-2017 when lifetimes are decreased by 50 %.............................. 57
Figure S1.42: Sensitivity analysis - End-of-Life (EoL) outflows from stocks by treatment type for stock-building materials in the USA 1962-2017 when lifetimes are increased by 50 %............................... 57
Figure S1.43: Sensitivity analysis - total material stock with conservative baseline multiplier (used in main results) and with modified multiplier based on Wiedenhofer et al., 2021. 58
Figure S1.44: EoL outflows for scenarios, 'low growth continuation' (a), 'high growth return' (b) and 'additional reduction (c). 59
Figure S1.45: Prospective development of material stocks to 2100 in scenario 3: 'additional reduction'. 59
Figure S1.46: Historical inputs to stock and end-of-life (EoL) outflows from 1970-2017 and modelled inputs for 2018-2100 under the scenario conditions for scenario 1: 'low growth continuation' and scenario 3: 'additional reduction' 60

List of Tables

Table S1.1: Data sources used to compile time series of stock-building materials and material inputs to stock. 30
Table S1.2: Data sources for processing and manufacturing losses assumed in our model. 32
Table S1.3: Data sources of downcycling rates. 32
Table S1.4: Data sources of recycling flows and rates. 33
Table S1.5: End-use shares, lifetimes for end-uses and weighted lifetimes in 2017 and their data sources for material inputs to stock used in our model. 34
Table S1.6: Material multipliers for asphaltic road types 37
Table S1.7: Comparison of estimates of stocks of iron/steel in the USA for various years 46
Table S1.8: Comparison of estimates of stocks of aluminum in the USA for various years................. 46
Table S1.9: Comparison of stock estimates for copper in the USA for various years 47
Table S1.10: Comparison of End-of-Life recycling rates for iron/steel, aluminum and copper in the USA for various years 54
Table S1.11: Commodity codes used to estimate net trade in addition to Streeck et al. (2020). 60

1 General Notes on Use of the MISO-model

The MISO-model (Wiedenhofer et al., 2019) requires data for the following exogenous input parameters: input of stock-building materials *or* inputs to stock, processing loss rates, manufacturing and construction loss rates, lifetimes (mean and standard deviation), recycling rates *or* flows, downcycling rates *or* flows and trade in end-of-life (EoL) materials to estimate values for endogenous variables: input of stock-building materials *or* inputs to stock, material stocks, EoL outflows from stock, recycling flows *or* rates, downcycling flows *or* rates and final waste. For the mentioned items that are distinguished by '*or*', one of the two data inputs is derived from reported data sources (exogenous) and the remaining item is endogenously calculated, depending on data availability for each material (for specific data sources see Supporting Information S2).

In our study, we distinguish **4 material groups** and *13 stock types*. Each stock type is produced from one or more stock-building material. Solidwood, for example, is produced from industrial roundwood, and flat glass from limestone, soda ash and silica sands (see Table S1.*1*).

- **Biomass**: *Solidwood, Paper and Paperboard*
- **Metals**: *Iron/Steel, Aluminum, Copper, Other Metals*
- **Non-metallic Minerals**: *Concrete, Asphalt, Bricks (incl. Stones), Sand and Gravel in sub-base and base-course layers (primary and downcycled), Container Glass, Flat Glass*
- **Fossil Energy Carriers**: *Plastics*

As explained in section 2.1 and Figure 1 in the main text, the inflow-driven MISO-model follows several steps to estimate material stocks and EoL outflows from stocks. For most materials, information on inputs to stock was available for the USA and calculations to compute material stocks and EoL outflows were done as explained in section 2.1 in the main text. However, for glass, asphalt and for periods of time also for bricks, the input of primary stock-building materials (*sbm_prim*; identifier 5 in Figure 1 in main text; e.g. metal ore) was used as model input and required further calculation steps to derive inputs to stock. Primary stock-building material use was derived from economy-wide material flow accounting (ew-MFA) datasets or calculated from a combination of statistics on extraction and trade, as shown in Equation A (t = year, m = material):

$$\text{(A)} \quad sbm\_prim_m(t) = extraction_m(t) + import\_prim_m(t) - export\_prim_m(t)$$

From primary stock-building materials, processing wastes (e.g. loss of bark, tailings) are deducted to obtain domestic primary production (Eq. B-C). Domestic primary production is part of inputs to stock and was used as described in Eq. 1 in the main text.

$$\text{(B)} \quad processing\_waste\ (t) = \ sbm\_prim_m(t) * processing\_wastage\_rate_m(t)$$

$$\text{(C)} \quad prod\_prim_m(t) = \ sbm\_prim_m(t) - processing\_waste\ (t)$$

A claim of the input driven MISO approach is to maintain full consistency with ew-MFA, i.e. to consistently link primary inputs to stocks with extraction, trade and use of stock-building materials as reported in ew-MFA (see Figure 1 in main text). If primary production and subsequently inputs to stock (e.g. copper, solidwood) is not calculated from ew-MFA consistent data on stock-building materials as described above (see Eq. B-C), but sourced directly from statistical databases, we extrapolated ew-MFA consistent material flows by adding processing waste where appropriate (Eq. D): Primary copper production (metal content of ore), for example, is a primary input to stock and available from the database of the United States Geological Survey (USGS; Kelly & Matos, 2014; USGS, 2018, 2019). In ew-MFA accounts, copper extraction (ew-MFA code: A.2.3.1, Table S1.*1*) is reported as gross ore, i.e. including waste rock, which is not reported in the USGS database. To estimate gross ore extraction, we use information on the average ore grade of mined copper in the USA and add waste rock (Eq. D; i.e. processing waste) to reported copper metal primary production:

$$\text{(D)} \quad sbm\_prim_m(t) = \frac{prod\_prim_m\ (t)}{[1 - processing\_wastage\_rate_m\ (t)]}$$

To calculate the stock-building share of domestic material consumption (DMC; for Figure 7 in main text), we added net trade in raw, semi-finished and final products (*product_imports/exports*; e.g. copper metal, copper cable and copper contained in a car) to primary stock-building material use (Eq. E, (Krausmann et al., 2018)):

$$\text{(E)} \quad stock\ building\ DMC_m = \ sbm\_prim_m \ + \ product\_imports_m - product\_exports_m$$

An overview of sources for model inputs (stock-building materials, primary inputs to stock, processing and manufacturing and construction losses, recycling and downcycling, lifetimes) is shown in Table S1.*1*-Table S1.*5* and sources and calculations are described in detail in section 2 of the SI.

Following the calculation of inputs to stock described above, gross additions to stock (GAS), material stocks and EoL outflows can be calculated with the help of lifetime distributions (see main text Eq. 2-4). Mean material lifetimes for the use in lifetime normal distributions were taken from literature (see Table S1.*5*). Whenever possible, time-dynamic lifetimes were calculated by multiplying yearly data for material use in different end-uses (e.g. concrete use in buildings versus roads) by the lifetimes of the

respective end-uses. Concrete for example has data for three end-use types: buildings, roads/bridges and other uses (lifetimes of 75, 32 and 33, respectively; Table S1.*5*). In 2002, the end-use shares were 46.4% of concrete used for buildings, 33.3% for roads/bridges and 20.3% for other uses. With the lifetimes of end-uses and the respective shares we calculated a weighted average lifetime, which for concrete is 52 years (75*0.464+32*0.333+33*0.203). For the materials glass, sand and gravel, other metals, asphalt and bricks no time-dynamic end-use shares were available. Thus, they were treated as a single end-use category. For these materials, lifetimes were either kept constant from 1870-2017 (if only a single lifetime datapoint was available) or interpolated between lifetime datapoints for different years. For an overview of the assumed mean lifetimes from 1870 to 2017 please see Table S1.*5* and Figure S1.*1*. For further information on the chosen lifetime distribution, see Wiedenhofer et al., 2019.

EoL outflows in turn, can be recycled or downcycled and re-enter material stocks as secondary inputs to stock. The MISO-model either uses reported EoL recycling flows to calculate recycling rates, or reported EoL recycling rates to estimate flows, depending on the available data. Recycling rates for materials are calculated from model-endogenous EoL outflows, and the reported amount of (exogenous) EoL recycling flows. EoL recycling flows are calculated by multiplying EoL outflows by their corresponding (exogenous) EoL recycling rates from the literature. Downcycling flows are calculated via downcycling rates applied to the supply of EoL outflows (after recycling) of asphalt, concrete and bricks and stones. Aggregates demand which cannot be met by recycled and downcycled materials is fulfilled by primary material (newly extracted). Downcycling of materials other than asphalt, concrete and bricks and stones is not considered in this study. For an overview of the development of re- and downcycling flows and rates see Figure S1.*2*–Figure S1.*4*.

The MISO-model estimates the endogenous variables for the predefined spatial and temporal system boundaries. The temporal boundaries are based on a spin-up period which is necessary to derive robust initial values for stocks, outflows, waste and re-/downcycling flows in the starting year of the actual study period. The length of the spin-up period is based on the material with the longest mean lifetime estimated within the model (Krausmann et al., 2017). In this study, aggregates have the longest lifetime (80 years). Here we begin the spin-up period in 1800 and analyse results for the period 1870 to 2017.

In Section 2 of the SI we describe all data, data sources, assumptions and calculation procedures used for quantifying all parameters necessary to estimate stocks, EoL outflows, re-/downcycling and final waste for the USA for 1870-2017. In Section 3 we compare and validate results against literature studies. Section 4 contains all figures and tables as well as additional results.

2 Data for Material Flows and Parameters

2.1 Biomass

Paper and Paperboard

Stock-Building Materials, Processing Losses and Primary Inputs to Stock

Paper and paperboard (P&B) primary inputs to stock were calculated as P&B primary production plus imports minus exports. Data for P&B total production (primary and secondary), imports and exports is available in the online database of the United States Geological Survey (USGS; Kelly & Matos, 2014) for 1900-2014 and from FAO, 2019 for 1961-2017. There is a good fit between both sources in the overlapping period, hence we use USGS data for 1900-2014 and expand the series to 2017 with data from FAO, 2019. Because data on primary production was only available for 1965-2014, we subtracted assumed recycling flows from total P&B production to obtain primary inputs before 1965 and after 2014 (for recycling calculations see below). For data prior to 1900 we followed the approach suggested by Krausmann et al., 2017: SI-10 and extrapolated total inflows by multiplying the per capita consumption from 1900 with population data for 1800-1899 from Bolt et al., 2018.

To establish ew-MFA consistency over the entire period, we added processing losses (e.g. changes in moisture, debarking, losses in pulp-production) to primary production of paper. The corresponding stock-building material for P&B in ew-MFA is industrial roundwood (Table S1.*1*) which was available for 1961-2017 from FAO, 2019. The USGS online database (USGS, 2014) on the other hand reports data for processed primary (excluding secondary P&B production) wood products (forestry production) for 1965-2014. Wood production is reported in ew-MFA including bark, which is added to underbark production data from (FAO, 2019; Krausmann et al., 2018). Based on information from Krausmann et al. (2018: 31) for loss of bark (10 % of industrial roundwood) and data from USGS and FAO we estimated processing losses for every year for the period 1961-2014 according to equation (1) and added them to primary P&B production. For 2015-2017 we kept processing losses constant at the level of 2014. For the period 1800-1965 we estimated the average processing losses for 1965-2014 (54 %) and added them to primary P&B production.

$$(1)\ Processing\ Losses\ (\%)_{IndustrialRoundwood} = 1 - \frac{Primary\ Forestry\ Production}{Industrial\ Roundwood\ Production * 1.1}$$

Manufacturing Losses

Since the online databases of USGS (Kelly & Matos, 2014) and FAO, 2019 report finished paper production, imports and exports, we assumed that manufacturing losses were already included in processing losses.

Lifetimes

To calculate average lifetimes, we calculated the shares of "printing and writing paper" and "other (short-lived) paper types" (e.g. newsprint, wrapping papers, household and sanitary papers) consumption based on FAO, 2019 data for 1961-2017. Prior to 1961, we kept shares constant at the level of 1961. End-use shares for P&B were multiplied with lifetimes for paper products (9 years for "printing and writing paper", 1 year for "other (short-lived) paper types") by Skog and Nicholson as reported in Penman, 2003. Weighted average lifetimes were 3±1 years (one standard deviation) in 2017 (Table S1.*5*).

Recycling

EoL recycling flows for P&B ("secondary production") are available from the USGS database from 1965 to 2014 (Kelly & Matos, 2014). According to Wernick et al., 1996, the share of recycled P&B production in total P&B production was approximately constant from 1900 to 1965. Before 1965 we thus kept the share of secondary production in total P&B production constant at the level of 1965 (26 %). For 2015 to 2017, we kept the share of secondary production constant at the 2014 level (64 %).

Solidwood

Stock-Building Materials, Processing Losses and Primary Inputs to Stock

Because solidwood recycling was assumed to be negligible (see below), solidwood primary inputs to stock are calculated as solidwood total production plus imports minus exports. Data for solidwood production and trade was available from the USGS database (Kelly & Matos, 2014) for the period of 1900 to 2014. "Lumber", "Plywood and veneer", "Wood panel products" and "Other industrial wood products" were identified as solidwood primary inputs to stock. For the period of 1962-2014 we also added net trade of cork and wood manufactures in the form of final goods (see main text and Streeck et al., 2020). For the period of 1800 to 1899, lumber production was taken from the Historical Statistics of the United States[1] (HSUS; Bureau of the Census, 1975; L98-112).[2] Additionally, since "Other industrial wood products"[3] were already a substantial input to stock in 1900 (50 % of lumber consumption) we kept this ratio constant before 1900 and added it to the reported lumber consumption. Values for primary inputs to stock from 2015 to 2017 were based on growth rates for

[1] We linearly interpolated lumber consumption between available data points from 1799 to 1899.

[2] The unit for lumber production in HSUS is board feet. Conversions between board feet and metric tonnes depend on the distribution of wood types, due to specific densities of coniferous and non-coniferous wood (Krausmann et al. (2018)). Based on estimations with Bureau of the Census (1975); Kelly and Matos (2014) data, we assume a conversion factor of 0.0011 metric tonnes for 1 board foot for 1800-1900.

[3] Other industrial wood products include "cooperage logs, poles and piling, fence posts, hewn ties, round mine timbers, box bolts, excelsior bolts, chemical wood, shingle bolts and miscellaneous items" (USGS (2014)).

industrial roundwood production derived from data reported in FAO, 2019. To obtain stock-building materials we added processing losses to solidwood production. The corresponding stock-building material for solidwood in ew-MFA is industrial roundwood. Processing losses are thus calculated in the same way as for paper and paperboard production (see calculations above).

Manufacturing Losses

Manufacturing losses were assumed at 5 %, based on scrapped wood products during construction (e.g. due to over-purchasing, customizing materials) as assumed in Cochran & Townsend, 2010.

Lifetimes

Average lifetimes were calculated based on end-use shares for solidwood (lumber, plywood/veneer, wood panel products and other industrial wood products) from the USGS database (Kelly & Matos, 2014) and the HSUS (Bureau of the Census, 1975). Lifetimes (75 years for lumber and plywood/veneer and 25 for wood panel products and other industrial wood products) were taken from Cochran & Townsend, 2010. Lumber is used for construction purposes with a long lifetime (75 years) and non-construction purposes with a shorter lifetime (e.g. consumer goods such as furniture, U.S. EPA, 2015). However, no information on the exact distribution of lumber end-uses over time could be found. Based on U.S. EPA, 2015 we assumed that 20 % of lumber consumption is used for non-construction applications (assumed lifetime: 25 years) and 80 % is used for construction purposes (lifetime: 75 years) for 1800-2017. Weighted average lifetimes were calculated at 63±7 years (one standard deviation) for 2017 (Table S1.5).

Recycling

Solidwood recycling was assumed negligible and recycling rates set to 0 % (Krausmann et al., 2017).

2.2 Metals

Iron/Steel

Stock-Building Materials, Processing Losses and Primary Inputs to Stock

Iron/steel primary inputs to stock were calculated as steel primary production plus net trade (imports minus exports) plus changes in inventories. Total (primary and secondary) production data was taken from the USGS database for 1900-2015 (Kelly & Matos, 2014) and the Mineral Commodity Summary of 2018 for 2016-2017 (USGS, 2018) and cross-checked with data from the World Steel Association (World Steel Association, 2019).[4] Overall there is a very good fit between both databases. For 1864-

[4] We compare crude steel production between the two sources but use steel product shipments from Kelly and Matos (2014) as primary inputs to stock.

1899 we used data from the HSUS (Bureau of the Census, 1975): P231-300. Because total production always includes both primary and recycled inputs to stock, we subtracted recycling flows from total production to obtain primary production (for the calculation of recycling flows see below). Prior to 1864 we followed the approach suggested by Krausmann et al., 2017: SI-10 and extrapolated total inflows by multiplying per capita consumption from 1864 with population data for 1800-1863 from Bolt et al., 2018. For iron/steel net trade, data from the USGS database (Kelly & Matos, 2014) was cross-checked with statistics from UNSD, 2019a. Trade in both databases includes semi-finished products but USGS excludes final end-use products (e.g. vehicles, machinery). Therefore, we used net trade data from UNSD, 2019a for the period of 1962 to 2017 (see main paper and Streeck et al., 2020). For 1913 to 1961 we used data from USGS (Kelly & Matos, 2014) for iron/steel trade. Trade data for iron/steel was not available prior to 1913, thus we assumed that production equals consumption before 1913. Net trade was 6 % of steel production annually on average for 1914-1950 (Kelly & Matos, 2014), the impact of neglecting net trade in the 19th century on results should thus be small.

Additionally, pig iron, besides being used as input for steel production, can be directly cast into cast iron stocks. The pig iron going to cast iron stocks was added to steel inputs described above. To calculate cast iron, we used pig iron casting shares from different data sources. Until 1946 we used the medium casting shares assumed by Pauliuk et al., 2013. For 1984-1998 and 2010-2017 we derived casting shares from data of American Foundry Society, 2011-2020; World Steel Association, 2019. Years in between were linearly interpolated.

Iron/steel is produced from iron ore, which is reported as gross ore (i.e. including waste rock) in the USGS database Kelly & Matos, 2014 and USGS, 2018 for the period of 1900-2017. Ew-MFA consistent apparent consumption of iron was calculated as iron ore production plus iron ore imports minus iron ore exports plus steel imports minus steel exports (using data from Kelly & Matos, 2014; UNSD, 2019a). Prior to 1900, we assumed iron ore grades of 50 % (Krausmann et al., 2018), while losses occurring during other processing steps of steel production (steelmaking, casting, rolling/forming) were assumed at 15 % (Cullen et al., 2012). To ensure ew-MFA consistency before 1900 we added these processing losses to primary production of iron/steel. Total processing losses from iron ore to iron/steel are thus assumed at 58 % from 1800 to 1899.

Manufacturing Losses

According to Cullen et al., 2012, fabrication losses occurring during the global production of steel end-use goods are 15 %. Fabrication losses are almost entirely recycled by manufacturers, but 15 % are lost during the recycling process of preconsumer scrap. Manufacturing losses are thus assumed as the share of fabrication losses that is lost, i.e. at 2.2 %.

Lifetimes

Weighted average lifetimes were calculated based on end-use shares (containers, transportation, construction, steel service centres and other) from the USGS database for 1979-2003 (Kelly & Matos, 2014) and Mineral Commodity Summaries for 2004-2017 (USGS, 2019). Prior to 1979, we kept end-use shares constant at the level of 1979. Lifetimes for different end-uses were taken from Müller et al., 2011.[5] Weighted average lifetimes were calculated at 34±4 years (one standard deviation) for 2017 (Table S1.*5*).

Recycling

The USGS database (Kelly & Matos, 2014) reports iron and steel scrap consumption for 1939-2015. However, the database does not specify end-of-life recycling flows (“old scrap”). Old scrap recycling flows for 1998-2017 were therefore obtained by multiplying shares of post-consumer (old) scrap from the Mineral Commodity Summaries of 1998 to 2017 (USGS, 2019) with total scrap consumption sourced from Kelly & Matos, 2014. Prior to 1996 we did not have information on shares of old scrap in total scrap consumption and thus kept the share of 1996 constant. Since steel recycling started in the 1900s (Wernick et al., 1996) and Kelly & Matos, 2014 report scrap consumption from 1939, we linearly interpolated scrap consumption for 1900-1939, starting from zero in 1900. While data for the period of 1998-2017 is therefore of good quality, recycling inputs prior to 1998 are rough estimates.

Aluminum

Stock-Building Materials, Processing Losses and Primary Inputs to Stock

Aluminum primary inputs to stock were calculated as aluminum primary production plus imports minus exports plus changes in inventories. Data for aluminum primary production was taken from the USGS database for 1900-2015 (Kelly & Matos, 2014), the Mineral Commodity Summary of 2018 (USGS, 2018) for 2016-2017 and the HSUS (Bureau of the Census, 1949): 152 for 1886-1900. Prior to 1886, aluminum production was non-existent (Aluminum Leader, 2018). Concerning aluminum trade, data from Kelly & Matos, 2014 was cross-checked with statistics from UNSD, 2019a. Trade in both databases includes imports and exports of crude aluminum and semi-manufactures but Kelly & Matos, 2014 exclude final end-use products. Therefore, we use net trade data from UNSD, 2019a for the period of 1962 to 2017 (see main text and Streeck et al., 2020). Prior to 1911, no trade data is reported in Bureau of the Census, 1949; Kelly & Matos, 2014, and we assumed that aluminum production is equal to

[5] Life times for the category “other” were assumed to be the average of “machinery” and “others” in Müller et al. (2011). The life time of the end-use category “Service centers and distributors” was assumed to be the average of the life times of the other four categories (construction, transportation, containers and other).

consumption. The USA became a major net importer of aluminum only from the 1980s onwards, impacts on results should thus be negligible (Chen & Graedel, 2012).

Aluminum is either produced from bauxite or from alumina, for which data is available from 1900 to 2017 in Kelly & Matos, 2014; USGS, 2018. Stock-building materials for aluminum are equal to bauxite production plus bauxite imports minus bauxite exports plus alumina imports minus alumina exports plus aluminum imports minus aluminum exports. Prior to 1900 we estimate bauxite consumption based on USGS, 2018: 31 which states that "As a general rule, 4 tons of dried bauxite is required to produce 2 tons of alumina, which, in turn, produces 1 ton of aluminium". Based on this information we assume that processing losses are 75 % from 1886 to 1900 and estimate bauxite consumption by multiplying aluminum primary production by the factor 4.

Manufacturing Losses

Manufacturing losses were derived from Chen & Graedel, 2012. Losses during the production of semi-finished and final products are 3 and 1 % respectively. Combined manufacturing losses were thus assumed at 3.7 %.

Lifetimes

Weighted average lifetimes were calculated based on end-use shares (construction, consumer durables, containers and packaging, electrical, machinery and equipment, transportation, other) from Chen & Graedel, 2012 for 1960-1975, the USGS database for 1975-2003 (Kelly & Matos, 2014) and Mineral Commodity Summaries for 2004-2017 (USGS, 2019). Prior to 1960, shares were held constant. Lifetimes were taken from Chen, 2013. We used their mid-values for mean lifetimes and standard deviations. Weighted average lifetimes were calculated at 22±3 years (one standard deviation) for 2017 (Table S1.*5*).

Recycling

End-of-Life recycling flows for aluminum ("old scrap secondary production") were taken from the USGS database for the period of 1939 to 2015 (Kelly & Matos, 2014) and from the USGS, 2018 for 2016-2017. Prior to 1939 (the last year for which old scrap secondary production is reported in Kelly & Matos, 2014) we set recycling of old scrap to zero.

Copper

Stock-Building Materials, Processing Losses and Primary Inputs to Stock

Copper primary inputs to stock were calculated as copper primary production plus imports minus exports plus changes in inventories. Data for copper primary production was taken from the USGS

database for 1900-2015 (Kelly & Matos, 2014), the Mineral Commodity Summary of 2018 for 2016-2017 (USGS, 2018) and Bureau of the Census, 1949: 150-151 for 1845-1899. Prior to 1845, copper production was small (below 100 t/yr) and thus set to zero. Concerning copper trade, data from Kelly & Matos, 2014 was cross-checked with trade statistics from UNSD, 2019a. However, exports and imports were substantially lower in USGS which only include refined copper, since UNSD, 2019a also reports several other semi-finished copper products and final goods. Therefore, we used trade data from UNSD, 2019a for the period of 1962-2017 to estimate total copper consumption (see main text and Streeck et al., 2020). For 1900-1961 we used data from Kelly & Matos, 2014 and for 1870-1899 we used copper trade data from Gierlinger & Krausmann, 2012. Prior to 1870, no trade data is available, and we assume that production equals consumption.

Since copper use is reported as metal content and copper extraction must be reported in gross ore in ew-MFA, we assume declining ore grades from 2.5 % in 1845 to 0.5 % in 2017 (Gierlinger & Krausmann, 2012; Wang et al., 2015) to extrapolate gross ore from metal content. Apart from separating copper from waste rock, an additional amount of copper is lost during smelting and other processes (Graedel et al., 2002). These losses were omitted, due to their comparatively small size. Processing losses were thus assumed at 97.5–99.5 %.

Manufacturing Losses

Manufacturing losses were assumed at 1 %, based on Glöser et al., 2013, and include losses during the fabrication of semi-finished products and final goods (Figure S15 in the SI of Glöser et al., 2013).

Lifetimes

Weighted average lifetimes for 1845-1999 are based on end-use shares reported by Spatari et al., 2005 and for 2000-2017 on end-use shares sourced from the USGS database and its Mineral Commodity Summaries (Kelly & Matos, 2014; USGS, 2019). Lifetimes for different end-uses were taken from Spatari et al., 2005.[6] Weighted average lifetimes were calculated at 34±7 years (one standard deviation) for 2017 (Table S1.5).

Recycling

End-of-Life recycling flows for copper ("old scrap secondary production") were taken from Kelly & Matos, 2014 for the period of 1906-2015 and from USGS, 2018 for 2016-2017. Recycling flows were very low in 1906 (2 % of total production). We therefore assumed that recycling was insignificant before 1906 and set recycling to zero.

[6] Since we use normal lifetime distributions, mean lifetimes by Spatari et al. (2005) were recalculated to be symmetric.

Other Metals

Stock-Building Materials, Processing Losses and Primary Inputs to Stock

Primary inputs to stock for other metals were calculated as other metals primary production plus imports minus exports. Other metals include nickel, lead, zinc, tin, gold, silver, platinum-group metals, thorium, arsenic, chromium, lithium, magnesium metal, manganese, mercury, strontium, tungsten, antimony, bismuth, cadmium, cobalt, gallium, indium, molybdenum, niobium (columbium), rare earths, selenium, tantalum, titanium, vanadium and zirconium. Primary production data for these metals was taken from Kelly & Matos, 2014 for 1900-2015 and the USGS, 2018 for 2016-2017. Trade data from Kelly & Matos, 2014 was cross-checked with data from UNSD, 2019a for nickel, lead, zinc and tin, while trade data for all other metals was only fragmentary. Overall there is a good fit between both sources. Therefore, we used trade data from Kelly & Matos, 2014 to estimate other metals consumption. Since no production and trade data was available prior to 1900 for most of the metals included, we used data from Bureau of the Census, 1975: M221-255 for manganese, lead and zinc for the period of 1801 to 1900, as they made up about 90 % of other metals consumption in 1900. To obtain gross ore for ew-MFA consistency, we used the average global ore grade of other metals (6 %; Krausmann et al., 2017). Similar to copper, we excluded processing losses other than waste rock as these losses are quite diverse for the various kinds of metals and no data were available; overall these losses are comparatively small. Processing losses for other metals were thus assumed at 94 %.

Lifetimes/Manufacturing Losses

Since by weight the largest amount of metals in this group is used in alloys with metals such as iron/steel, aluminum and copper (Gerst & Graedel, 2008; Kelly & Matos, 2014; Krausmann et al., 2017; UNEP, 2011), we used the average of the lifetimes and manufacturing losses assumed for iron/steel, aluminum and copper (Krausmann et al., 2017). Manufacturing losses for other metals were thus assumed at 2.3 % and average lifetimes at 30±5 years (one standard deviation) for 2017 (Table S1.5).

Recycling

End-of-Life recycling flows for other metals (“old scrap secondary production”) were taken from Kelly & Matos, 2014 for the period of 1906 to 2015 and from the USGS, 2018 for 2016-2017. Prior to 1906, reported secondary production levels of other metals in Kelly & Matos, 2014 is small (below 100 t/yr) and thus set to zero.

2.3 Non-Metallic Minerals

Bricks and Stones

Stock-Building Materials, Processing Losses and Primary Inputs to Stock

Data for brick production from 1869 to 1970 was taken from Bureau of the Census, 1975: P231-300.[7] We assumed that 550 bricks are equal to 1 tonne of clay bricks (Cochran & Townsend, 2010). For 1975-2017 data for miscellaneous clay (used for brick production) from the USGS database (Kelly & Matos, 2014) was used as a proxy for brick production. For bricks we used net trade data from UNSD, 2019a for the period of 1962-2017. Trade was not available for 1869-1961 and thus omitted. Dimension stone production and net trade for the years 1900-2015 was taken from USGS 2019. For 2016-2017, the mass of stones is assumed constant at the level of 2015. For data prior to 1869 we followed the approach suggested by Krausmann et al., 2017: SI-10 and extrapolated total inflows for bricks by multiplying per capita consumption from 1869 with population data for 1800 to 1868 from Bolt et al., 2018. Processing losses for clays (loss of moisture content during brick production) were assumed at 26 % and added to primary inputs for 1869-1970 because we used data for bricks production for this period. For 1975-2017, clay stock-building materials are equal to primary inputs to stock. Processing losses for stones were assumed at 0 %, primary inputs for stones are thus equal to stock-building materials (Krausmann et al., 2017).

Manufacturing Losses

Manufacturing losses for bricks and stones were assumed at 4 %, based on scrapped bricks and other clay products during construction processes (e.g. due to over-purchasing or customizing materials to fit specific applications) reported in Cochran & Townsend, 2010.

Lifetimes

Lifetimes are based on Cochran & Townsend, 2010. Bricks and stones were assumed to have the same lifetime. Lifetimes were assumed at 75±8 years (one standard deviation) in 2017 (Table S1.5).

Recycling and Downcycling

Brick recycling is insignificant at least since 1998 (USGS, 2018, 2019). We assumed that recycling rates for bricks and stones decrease from 15 % in 1800 to 0 % in 1960. Furthermore, we assumed a downcycling rate of 35 % for 1800-1920, which decreases to 10 % in 1970 and then grows to 47 % in 2017. Assumptions for re- and downcycling are very rough and based on Krausmann et al., 2017.

[7] We linearly interpolated bricks consumption for 1869-1879, 1879-1889, 1889-1895 and 1940-1943 and 1970-1974.

Concrete

Stock-Building Materials, Processing Losses and Primary Inputs to Stock

Concrete primary inputs to stocks are equal to concrete production, as concrete is hardly traded. Concrete production can be estimated based on cement consumption (Cochran & Townsend, 2010). To do so, we assumed that concrete contains 11 % (volume) of cement and that cement has a density of 3150 kg/m³ and concrete has a density of 2300 kg/m³ (Cochran & Townsend, 2010). Based on these assumptions we then estimated concrete production according to equation (2). Cement consumption (cement production plus imports minus exports plus stock changes) is available in Kelly & Matos, 2014 for 1900-2015 and in USGS, 2018 for 2016-2017. The data on cement consumption was cross-checked with data from Cembureau, 1998, 2005, 2017. Overall there is a good fit between both sources, hence we use USGS data for our estimates.[8] For 1818-1900, cement production was taken from Bureau of the Census, 1975: M188-204. Prior to 1818, cement consumption was small (below 5000 t/year) and we set concrete inputs to zero.

$$(2)\ Concrete\ Production\ (t) = \frac{Cement\ Consumption\ (t)}{0.11} * \frac{2300}{3150}$$

Raw materials for cement production (80 % limestone, 20 % clay and other raw materials) as reported in ew-MFA accounts were estimated based on equation (3) (Kapur et al., 2009). Data for sand and gravel (S&G) in concrete production was estimated by using equation (4) from Krausmann et al., 2018: 51. Processing losses for cement production were assumed at 42 % (mainly emissions of CO_2) and 0 % for sand and gravel (Krausmann et al., 2017).

$$(3)\ Raw\ materials\ for\ Cement\ Production\ (t) = Cement\ Production\ (t) * 1.7$$

$$(4)\ Sand\ and\ Gravel\ for\ Concrete\ Production\ (t) = Cement\ Consumption\ (t) * 6.1$$

Manufacturing Losses

Manufacturing losses were assumed at 3 %, based on scrapped concrete during construction (e.g. due to over-purchasing, customizing materials) reported in Cochran & Townsend, 2010.

Lifetimes

Average lifetimes were calculated based on end-use shares (buildings, roads/bridges, other) and lifetimes sourced from Cochran & Townsend, 2010.[9] We linearly interpolated shares between data

[8] We include both portland and masonry cement in our estimations. Concrete production from masonry cement was calculated in the same way as concrete production from portland cement.

[9] Since we use normal lifetime distributions, mean lifetimes for roads/bridges and other structures were recalculated to be symmetric.

points (1902, 1927, 1952, 1962, 1977, 1979, 1982, 2002) available from Cochran and Townsend 2010 and kept shares constant at the level of 2010 for 2011-2017. For 1818-1900 we kept shares constant at the level of 1900. Weighted average lifetimes were calculated at 52±6 years (one standard deviation) for 2017 (Table S1.*5*).

Recycling and Downcycling

We assumed that concrete recycling started in 1970 (similar to asphalt) and increased to 3 % in 1996, after which we assumed that it kept on increasing linearly to 5 % in 2017 (Kelly, 1998; Kelly & Matos, 2014; Krausmann et al., 2017; Wilburn & Goonan, 1998). Regarding downcycling, Wilburn & Goonan, 1998 report that in 1996, 50 % of concrete EoL waste was landfilled. The rest was used to replace natural aggregates in cement concrete (3 %), asphaltic concrete (4 %) or road base and others (43 %; Kelly, 1998). Sandler, 2003 states that estimates have placed re- and downcycling rates for concrete between 50 and 57 %.[10] Based on these studies we assumed a constant downcycling rate of 10 % before 1970, which linearly increases to 55 % in 2017.

Container Glass and Flat Glass

Stock-Building Materials, Processing Losses and Primary Inputs to Stock

Container and flat glass production was estimated based on data for soda ash consumption and coefficients derived from Ruth & Dell'Anno, 1997 who report that for the production of 1 kg of container (flat) glass, 0.22 (0.23) kg of soda ash, 0.65 (0.73) kg industrial sand and 0.19 (0.24) kg of limestone are needed. For the production of container glass, additionally 0.11 tons of Feldspar are used. We estimated glass production based on soda ash consumption. Soda ash is not only used for glass, but also for other products, such as chemicals and soaps. End-use shares for soda ash consumption are available for 1980-2003 in Kelly & Matos, 2014. End-use shares of soda ash consumption for container and flat glass production were held constant before 1980 and after 2003. To perform a cross-check, total glass production was also estimated based on industrial S&G consumption (silica sands). End-use shares for glass production from industrial S&G are available for 1975-2015 in USGS, 2019. For 1900-1975 shares of silica sands for glass production were held constant at the level of 1975.

Overall there is a good fit between estimates from soda ash and silica sands consumption. Glass production estimated from silica sands also includes fibre and other types of glass. For this reason (among others), estimates from silica sands generally tend to be higher than the estimates from soda

[10] Sometimes the terms recycling and downcycling are used interchangeably. Here we assumed that Sandler refers to combined re- and downcycling rates.

ash. Both estimations also agree well with the values for glass production shown in Ruth & Dell'Anno, 1997. We use glass estimations from soda ash, since we can explicitly distinguish between container and flat glass. Glass consumption was calculated by adding net trade sourced from UNSD, 2019a to production (SITC1 664, 665). Since we were not able to separate different kinds of glass net trade from UNSD, 2019a, we add total glass net trade to flat glass in 1962 to 2016 (see main paper and Streeck et al., 2020). For data prior to 1900 we followed the approach suggested by Krausmann et al., 2017: SI-10 and extrapolated total inflows by multiplying per capita consumption from 1900 with population data for 1800 to 1899 from Bolt et al., 2018.

Stock-building materials for flat and container glass (soda ash, silica sands, limestone and feldspar) were estimated based on soda ash consumption and production coefficients. For 1 kg of container and flat glass, 1.17 kg and 1.2 kg raw materials are needed, respectively. Processing losses were assumed at 17 % for container glass and 20 % for flat glass (Ruth & Dell'Anno, 1997).

Manufacturing Losses

Since most losses occurring during the manufacturing of glass are internally recycled, manufacturing losses were assumed at 0 % (Butler & Hooper, 2011).

Lifetimes

Lifetimes for container and flat glass were based on Wiedenhofer et al., 2019. Container glass lifetimes decrease from 5 years in 1900 to 1.5 years in 1970 and then increase to 3 years in 2010. Flat glass lifetimes decrease from 50 years in 1900 to 30 years in 1970 and then remain constant.

Recycling

Recycling rates for container glass were based on data for glass municipal solid waste and recycling from U.S. EPA, 2020. We linearly interpolated recycling rates between available data points (1960 1970, 1980, 1990, 2000, 2005, 2010-2017). Recycling rates increased from 2 % in 1960 to 31 % in 2017. Prior to 1960 we assumed that recycling was negligible and set recycling rates to 0 %. We assumed that flat glass was generally not recycled (Wiedenhofer et al., 2019).

Aggregates (Primary)

Primary Inputs to Stock

Aggregates (sand, gravel, crushed stone) for sub-base and base-course layers in built infrastructures were estimated in the MISO-model. For buildings, we assumed that "an average of 70 kg of aggregate […] per Mg of concrete and 45 kg per Mg of bricks" (Krausmann et al., 2017): SI-9 is used for sub-base

layers (Figure S1.*10*). For sub-base and base-course layers in roads, we estimated aggregates demand according to equation (5).

$$(5)\ Aggregate\_base\_course = Asphaltcons * base\_course\_multiplier$$

Information on asphalt consumption (*Asphaltcons)* was derived from USGS (Kelly & Matos, 2014) and IEA, 2019 (see section on Asphalt below). To calculate the term *base_course_multiplier*, two kinds of coefficients were needed: 1) the overall ratio of asphalt to sand and gravel stocks in roads and 2) the proportion of asphalt used each year for new roads. These coefficients were estimated as follows.

1) Overall proportion of stocks of asphalt to sand and gravel in roads:

Information on road width, length and depth for different road types in the USA are given by (Miatto, Schandl, Wiedenhofer et al., 2017). They distinguish between 7 road types of which 4 are asphaltic. These 4 types are "low type pavement" (LTP), "intermediate pavement" (IP), "high flexible pavement" (HFP) and "high composite pavement" (HCP). Based on this information and specific densities for asphalt and sand and gravel (Krausmann et al., 2018) we calculated the amount of asphalt and sand and gravel contained in each road type (Table S1.*6*). We calculated multipliers for each road type, according to equation (6).

$$(6)\ Multiplier_{roadtypex} = \frac{Sand\ and\ Gravel_{roadtypex}}{Asphalt_{roadtypex}}$$

To estimate the weighted multiplier for combined road types, information on the development of the total road kilometres for asphaltic road types is needed. Miatto, Schandl, Wiedenhofer et al., 2017 report road kilometres for all road types for 1905-2015 (Figure S1.*5*). Four time periods of the development of the road network in the USA can broadly be distinguished: 1905-1940, 1940-1980, 1980-2008 and 2008-2015. In the first period, growth for mileage of all road types was almost the same. From 1940 on, growth of high composite pavement roads ceased while the three remaining road types experienced strong growth. For 1980-2008, low type and intermediate pavement kilometres first stopped to increase and then grew slightly until 2008, while the high flexible pavement road type continued to grow until 2008. From 2008, expansion of asphaltic roads almost ceased.

For 1905 to 1940, we calculated the share of each road type (LTP, IP, HFP, HCP) in total new road construction (Figure S1.*6*). We then estimated the weighted multiplier for each year according to equation (7). Prior to 1905 we kept the multiplier constant at the level of 1905.

$$(7)\ MultiplierTotal_{yearx} = ShareNewRoadConstructionLTP_{yearx} * MultiplierLTP + ShareNewRoadConstructionIP_{yearx} * MultiplierIP + ShareNewRoadConstructionHFP_{yearx} * MultiplierHFP + ShareNewRoadConstructionHCP_{yearx} * MultiplierHCP$$

For 1941 to 1980, we calculated the share of the road types LTP, IP and HFP in total new road construction (Figure S1.*7*). We then estimated the weighted multiplier according to equation (8).

$$(8)\ MultiplierTotal_{yearx} = ShareNewRoadConstructionLTP_{yearx} * MultiplierLTP + ShareNewRoadConstructionIP_{yearx} * MultiplierIP + ShareNewRoadConstructionHFP_{yearx} * MultiplierHFP$$

For 1981-2017 we assumed that the multiplier was equal to the multiplier of the high flexible pavement type, since the growth of kilometres of this road type was the main driver of asphaltic road construction. The development of the total multiplier for asphaltic roads (*MultiplierTotal*) is shown in Figure S1.*8*.

2) The proportion of asphalt used each year for new roads

To obtain coefficients for the proportion of asphalt used each year in new roads, we used information on the usage of materials for new road construction by Miatto, Schandl, Wiedenhofer et al., 2017. They state that "the share of material requirements has progressively shifted from new construction to maintenance. In the early years, we expect that about 70% of the yearly material inflows would have been required for expanding the network. This share progressively diminished, dropping on average below 50% in 1966, and arriving […] at an average share of 20% in 2015" (SI. 11). Based on this information we assumed that in 1870 (when use of asphalt started, NAPA, 2019a) asphalt consumption was used entirely for new roads, while in the years 1905/1966/2015, 70/50/20% of asphalt was used for new road construction, respectively. Between these years, we linearly interpolated shares of asphalt for new roads (Figure S1.*9*). For 2016-2017 we kept the share of asphalt for new roads constant at the level of 2015.

Finally, we multiplied the *MultiplierTotal* by the share of asphalt used for new roads (equation 9) to obtain the *base_course_multiplier*, which we use to calculate aggregates consumption for sub-base and base-course layers of roads (equation 5). For the development of the multiplier see Figure S1.*10*.

$$(9)\ Base\_course\_multiplier_{yearx} = MultiplierTotal_{yearx} * Share\ of\ Asphalt\ for\ new\ road\ construction_{yearx}$$

Stock-Building Materials, Processing and Manufacturing Losses, Lifetimes and Recycling

Processing and manufacturing losses for aggregates in sub-base and base-course layers were assumed at 0%, average lifetimes were assumed at 80±24 years (one standard deviation) and recycling rates were assumed constant at 77% for the entire period (1800-2017) based on Krausmann et al., 2017; Wiedenhofer et al., 2019.

Asphalt

Stock-Building Materials, Processing Losses and Primary Inputs to Stock

Asphalt primary inputs to stock are equal to asphalt production, as asphalt is usually not traded. Asphalt concrete is typically composed of 5% bitumen and 95% sand and gravel (S&G, excluding air and other small components) and can thus be estimated based on data available for the use of these materials (Cochran & Townsend, 2010; Miatto, Schandl, Wiedenhofer et al., 2017). Data for S&G consumption for asphalt production and other applications is available in Kelly & Matos, 2014; USGS, 2019 for 1975-2017. However, about 50% of total S&G consumption is reported as unspecified use. Estimating asphalt from S&G consumption would thus likely result in an underestimation of total asphalt consumption. Data for bitumen production/consumption is available since the early 20th century from different sources (IEA, 2019; Kelly & Matos, 2014; UNSD, 2019b, 2020). Miatto, Schandl, Wiedenhofer et al., 2017 also estimated bitumen consumption for road construction in the USA, using a bottom-up approach and distinguishing between virgin and recycled asphalt production. Apart from Miatto, Schandl, Wiedenhofer et al., 2017, all sources of bitumen consumption agree well with each other. The estimates of Miatto, Schandl, Wiedenhofer et al., 2017 are significantly smaller because exact construction standards (thickness of layers, width) for certain road types in the USA are unknown and this distorts bitumen consumption estimates by the bottom-up approach of Miatto, Schandl, Wiedenhofer et al., 2017. Therefore, we decided to use statistics on bitumen consumption from USGS ("Asphalt and Road Oil" in Kelly and Matos, 2014)[11] for 1905-2013 and data from IEA, 2019 for 2014-2015. For 2016-2017 we kept asphalt consumption constant at the level of 2015. Since asphalt usage began in the 1870s (NAPA, 2019a), we assumed that asphalt production linearly increased from 0 tonnes in 1869 to 807,500 tonnes in 1905. We assumed that 85 % of all bitumen is used for road construction, the rest being used for roofing (asphalt shingles) and other uses (Pyshyev et al., 2016). We excluded bitumen used in these (comparatively small) applications from our stock estimate and focus on bitumen in asphalt. Asphalt consumption was estimated based on equation (10):

$$(10)\ Asphalt\ Production\ (t) = \frac{Bitumen\ consumption\ (t) * 0.85}{0.05}$$

Processing losses for bitumen and sand and gravel were assumed at 0 %, based on Krausmann et al., 2017. Stock-building materials are therefore equal to primary material inputs to stock for asphalt.

Manufacturing Losses

Manufacturing losses were assumed at 0 % based on (Cochran & Townsend, 2010).

[11] Asphalt and Road Oil is included in the "Organics (nonrenewable)" section of USGS.

Lifetimes

Average lifetimes were based on estimated lifetimes for industrial countries from Wiedenhofer et al., 2021 and set at 35±4 years (one standard deviation) in from 1870 to 2017.

Recycling and Downcycling

Data on asphalt recycling flows was available for the years 1993 (USDT, 1993), 1996 (Wilburn & Goonan, 1998) and 2009 to 2017 (NAPA, 2019b). Since the first sustained recycling efforts only began in the 1970s (FHA, 2016), we calculated the share of recycled to new asphalt for available data points and linearly interpolated between them, starting from 0% in 1970. Regarding downcycling, Wilburn & Goonan, 1998 report that 8% of asphalt pavement debris was downcycled in 1996. According to NAPA, 2019b, 4-12 % of reclaimed asphalt pavement (RAP) from roads and parking lots was downcycled annually from 2009 to 2017. Based on this information we assume a downcycling rate of 10% in 1970, which linearly decreases to 5% in 2017. Prior to 1970, asphalt downcycling rates were assumed constant at 10 %.

2.4 Fossil Energy Carriers

Plastics

Stock-Building Materials, Processing Losses and Primary Inputs to Stock

Data for plastics production is published on an annual basis by the American Chemistry Council (ACC, 2019) and currently covers the period from 1973 to 2018. However, this data was not accessible for this study. Therefore, we used a different approach to estimate plastics production in the USA: Global plastics (polymer resin and fiber) production for the period of 1950 to 2015 is reported in Geyer et al., 2017. Plastics Europe, 2019 additionally shows production shares by different regions and countries for 2006-2016. Although Plastics Europe does not explicitly show production shares for the USA, they show production shares for the NAFTA region. We held shares of plastics production prior to 2006 for the NAFTA region constant at the level of 2006[12] and multiplied the relative share of US GDP in the NAFTA region with the amount of plastics produced by the whole NAFTA region to obtain total plastics production values for the USA. Relative shares of GDP of the USA, Canada and Mexico were calculated based on data from Bolt et al., 2018. Prior to 1950 we assumed that plastic production was negligible and thus set the primary inputs to stock to zero. Values for 2016 and 2017 were based on the average annual growth rate of 2010-2015. Since data for plastics net trade was not available, we assumed that production equals consumption for 1950-2017. Since information on processing losses in the

[12] For 2017, we keep the share constant at the level of 2016.

petrochemical industry was not available, we assumed that stock-building materials are equal to primary inputs to stock (Krausmann et al., 2017; Wiedenhofer et al., 2019). This probably underestimates the mass of stock-building materials.

Manufacturing Losses

Manufacturing losses were estimated based von a substance flow analysis study by van Eygen et al., 2017 for Austria. According to van Eygen et al., 2017, 10% of plastics production is lost during the manufacturing process. No information on the internal recycling rates of manufacturing losses for the USA could be found. Based on EoL recycling rates for 2015 from U.S. EPA, 2020, we assumed that 9 % of manufacturing losses are recycled internally. Manufacturing losses were thus assumed constant at 9%.

Lifetimes

Since end-use shares for plastics for the USA were not accessible, we calculated lifetimes based on end-use shares for Europe (Plastics Europe, 2019), because end-use shares are relatively similar for Europe and the USA (Geyer et al., 2017). Since Plastics Europe production shares do not include fibre, we assumed a constant share of 11.5% for textiles based on Geyer et al., 2017. All other plastic shares were decreased by 2.3% to take this into account. Lifetimes for different end-uses were taken from Bento et al., 2016; Geyer et al., 2017. Weighted average lifetimes were calculated at 10±2 years (one standard deviation) for 2017 (Table S1.*5*).

Recycling

Recycling rates for plastics were based on U.S. EPA, 2020 and were calculated as recycled plastics divided by total plastics municipal solid waste. We linearly interpolated recycling rates between available data points (1980, 1990, 2000, 2005, 2010-2017). Plastics recycling started in 1980 (0.3 %) and increased to 8 % in 2017.

Bitumen

See section 2.3 (asphalt).

2.5 Additional details on trade data

As reported in the Supporting Information S2 2.1-2.4, the commodity codes reported in Streeck et al., 2020 were partially used to capture trade in semi-manufactures and final products from (UNSD, 2019a). In addition to the commodity codes listed in Streeck et al., 2020, codes in Table S1.*11* were also taken into account.

3 Comparison with Other Studies, Uncertainty & Sensitivity

We generally find good agreement of our results with stock estimates from previous studies. Fishman et al. (2014) reported 107.5 Gt of total material stocks in 2005, whereas herein we find 95 ±4.3 Gt (2 standard deviations of MCS), which is between 8-15% lower. Differences arise from a more elaborate and detailed calculation of inputs to stock from raw materials in this study, the explicit consideration of secondary material inputs and differences in lifetime assumptions (see SI 3.1-3.4 & SI 4 for further explanations). We also find a relatively good agreement of our results with stock estimates for other industrialized countries (UK, Streeck et al., 2020; Japan, Fishman et al., 2014), materials specific studies on US stocks of concrete and asphalt (Kapur et al., 2008; Miatto, Schandl, Wiedenhofer et al., 2017), iron & steel, aluminium and copper (Chen & Graedel, 2012; Liu et al., 2011; Müller et al., 2006; Sullivan, 2005) as well as end-of-life estimates for concrete, bricks, glass, paper and paperboard, solidwood and plastics (U.S. EPA, 2015) and recycling flows for paper and paperboard, glass and plastics (U.S. EPA, 2020) and recycling rates for iron & steel, aluminium and copper (Chen, 2013; Fenton, 2004; Goonan, 2009; Graedel et al., 2004; Plunkert, 2006; Wang et al., 2007)

We further evaluated uncertainties via Monte-Carlo Simulations and conducted sensitivity analyses. We find that uncertainty is relatively low for total stocks (±5%) and ranges between ±3% and ±13% for individual stock types, the only exception being container glass with ±26%. Uncertainty is higher for EoL outflows (±17% for total flows) and ranges from ±11% to ±60% for individual materials. Since results of inflow-driven models strongly depend on the assumed mean lifetimes (Miatto, Schandl, & Tanikawa, 2017; Wiedenhofer et al., 2019) we tested the effects of a ±50% variation of mean lifetimes and find that total stock values are either 21% lower or 19% higher in 2017 (see Figure S1.*39*). Total EoL outflows were 30% lower or 42% higher in 2017 and also variation on the level of individual materials was moderate (see Figure S1.*40*, Figure S1.*41*, Figure S1.42). Another parameter with high uncertainty is the large flow of aggregates into stocks, which is notoriously underreported and hard to estimate (Miatto, Schandl, Fishman et al., 2017). Herein, we utilized US specific information from Miatto, Schandl, Wiedenhofer et al. (2017), which gives a conservative, relatively low estimate, but is based on empirical data. Using broader assumptions developed in previous work on the global level by some of the authors (Krausmann et al., 2017; Wiedenhofer et al., 2021) we arrive at a considerably higher stock of 141 Gt (+37%) in 2017 (see Figure S1.43). We note that this topic requires further research but herein use the more conservative estimates.

3.1 Biomass Comparison

Paper and Paperboard/Solidwood

The estimates for biomass stocks presented here are on average 47% smaller than the stock estimates by Fishman et al., 2014 but exhibit a very similar development over time (Figure S1.*11*). Fishman et al., 2014 start from industrial roundwood as reported in ew-MFA because they use ew-MFA data from Gierlinger & Krausmann, 2012 to estimate stocks. Fishman et al., 2014 deduct only 10 % losses from industrial roundwood to obtain inputs to stock. This means they only take loss of bark into account but ignore other losses during wood processing industries (e.g. in saw mills), including changes in moisture content (Krausmann et al., 2018). Here we use data on primary inputs of paper and paperboard and solidwood (lumber, plywood and veneer, wood panel products and other industrial wood products) from Kelly & Matos, 2014 and UNSD, 2019b. We estimate that the processing losses from industrial roundwood to primary inputs to stock are significantly larger (54 % on average from 1964-2014) than the losses assumed by Fishman et al., 2014. For these reasons, we arrive at a lower biomass stock. Because of the smaller stock, final waste flows (defined as End-of-Life outflows minus re- and downcycling flows) calculated here are also smaller than the estimates of Fishman et al., 2014 (Figure S1.*30*). Additionally, final waste dynamics are different between the two estimates, as we assumed dynamic lifetimes over time, while Fishman et al., 2014 kept lifetimes constant and also omitted recycling in their calculations of biomass stocks (we included recycling for paper and paperboard).

Concerning EoL outflows and recycling rates, our estimates for paper and paperboard (P&B) and solidwood agree well with estimates by EPA (U.S. EPA, 2015, 2016, 2018, 2020). Values for P&B EoL outflows and recycling rates show a similar trend over time but MISO-estimates are generally higher (Figure S1.*23*, Figure S1.*24*). P&B results of the MISO-model are higher most likely because EPA uses data from the American Forest & Paper Association (EPA, 2014) to estimate outflows and recycling, while we use data from Kelly & Matos, 2014. MISO-results for solidwood EoL outflows are slightly higher because we include lumber, plywood and veneer, wood panel and industrial wood products in our estimations (Figure S1.*24*) while EPA uses a material flow approach to estimate demolition debris of buildings, roads and bridges in the USA and therefore does not include industrial wood products (U.S. EPA, 2015).

3.2 Metals Comparison

Iron/Steel

Table S1.*7* shows that estimates for iron and steel stocks by Müller et al., 2006; Müller et al., 2011; Rauch, 2009; Sullivan, 2005 agree very well with our estimate. Only the estimate by Brown, 1954 for

1950 is much lower than our estimate (-39%); since we had no access to the original study but refer to a reference by Gerst & Graedel, 2008, we have no information available to explain this difference.

Time-series data for iron/steel stocks were published by Fishman et al., 2014; Müller et al., 2011; Pauliuk et al., 2013. Per capita stocks from Müller et al., 2011; Pauliuk et al., 2013 agree quite well with our results (see Figure S1.*12* and Figure 4 in Müller et al., 2011 and Figure S1.*13* and Figure 4 in Pauliuk et al., 2013).

Concerning the results of Fishman et al., 2014, the iron/steel stock estimates in our study are substantially larger (Figure S1.*14*). Fishman et al., 2014 start from iron ore as reported in the ew-MFA data from Gierlinger & Krausmann, 2012 to estimate iron/steel stocks. Fishman et al., 2014 deduct 80% losses from iron ore to obtain inputs to stock. In this study we use data on steel consumption from Kelly & Matos, 2014 to estimate iron/steel stocks. Since our results agree well with the results of other studies which specifically analyse iron and steel stocks, we assume that Fishman et al., 2014 have underestimated iron stocks, because their assumed loss factor was too high. Since stocks are substantially larger in our study, this also explains the larger final waste flows (defined as EoL outflows minus re- and downcycling flows, Figure S1.*31*) compared to the results of Fishman et al., 2014. Additionally, final waste dynamics over time differ, as the lifetimes assumed in this study (29-35 years) are smaller than those used by Fishman et al. (2014; 50 years) and we also included recycling in our calculations of iron/steel stocks. The estimated EoL recycling rates for iron/steel in this study agree quite well with recycling rates in the literature (Table S1.*10*).

Aluminum

For aluminum our estimates agree reasonably well with results from most other studies (range of ±30%, Table S1.8). Several studies have found that aluminum stocks are not very sensitive to different lifetime distribution models but are sensitive to variations in mean lifetimes, which can differ between studies (Chen & Graedel, 2012; Liu & Müller, 2013). Therefore, we consider variations of ±30 % as an acceptable range (Liu & Müller, 2013). McMillan et al., 2010 arrive at a significantly lower stock (-88%), which may be a result of longer lifetimes assumed by our study and the inclusion of more comprehensive trade data as is the case of results for (Chen & Graedel, 2012). Time-series data for stocks and EoL outflows by Chen & Graedel, 2012; Liu et al., 2011; Liu & Müller, 2013 agrees quite well with our results for the twentieth century (for stocks see Figure S1.16 and Figure 3 in Liu et al., 2011/Fig. 10a in Chen & Graedel, 2012 and Figure S1.15 for a comparison between stocks from Liu & Müller, 2013 and this study; for EoL outflows see Figure S1.25 and Fig. 6b in Chen & Graedel, 2012). Differences in values for the 20th century may arise from variations in lifetimes. The estimated EoL recycling rates agree quite well with EoL recycling rates in the literature (Table S1.10).

Copper

Our estimate of copper stocks agrees well with results of most other studies (range of ±30 % for later years, Table S1.*9*). The estimates of Ingalls, 1935; Merrill, 1949, 1959 were obtained from Gerst & Graedel, 2008, the original studies were not accessible. Additionally, the calculation method used by Gerst & Graedel, 2008 to determine material stocks for Nathan Associates, 2004 could not be identified. As a result, we cannot explain the larger differences between these results and our estimates. The estimated EoL recycling rates of this study agree quite well with EoL recycling rates in the literature (Table S1.*10*).

Other Metals/Aluminum/Copper

Estimates of stocks for the aggregate of other metals as defined in this study (i.e. including all metals other than iron, aluminum and copper) are not available in the literature.[13] However, Fishman et al., 2014 have estimated stocks for aluminum, copper and other metals as a group (which they define as "other metals"). Figure S1.*17* shows that the development of non-ferrous (i.e. excluding iron) metals stock estimates by Fishman et al., 2014 is very similar to the development of non-ferrous metals in this study. However, the results of Fishman et al., 2014 are on average 70 % larger than our results. This also impacts final waste flows, which are substantially bigger than the estimates in this study (Figure S1.*32*).

Fishman et al., 2014 start from "other metals" ores (i.e. excluding iron) as reported in the ew-MFA data from Gierlinger & Krausmann, 2012. Fishman et al., 2014 deduct 90 % processing and manufacturing losses from gross ore to obtain actual inputs to stock. In this study we used data on primary inputs for aluminum, copper and other metals to estimate stocks. We estimate processing losses of stock-building materials of 75% for aluminum, 97.5-99.5% for copper and 94% for other metals and deducted manufacturing losses of 3.7%, 1% and 2.3% from aluminum, copper and other metals primary inputs respectively. Additionally, lifetimes for other metals assumed by Fishman et al., 2014; (50 years) are substantially higher than the lifetimes assumed in this study (20-40 years). Given that we use more detailed information on primary inputs to stock for aluminum, copper and other metals and our results for aluminum and copper agree well with the results of other studies, we assume that Fishman et al., 2014 overestimated non-ferrous metals stocks.

[13] In-use stock estimates are available for gold, lead, tungsten, zinc at the national level and stocks for chromium and nickel are available at the state level (Gerst and Graedel, 2008).

3.3 Non-Metallic Minerals Comparison

Concrete

To compare concrete stocks, we use estimates of cement stocks by Kapur et al., 2008 and Cao et al., 2017 and extrapolate concrete stocks by applying equation 2 (see section 2.3). Overall, the estimates of these studies agree well with our results in terms of stock development Figure S1.*18*, Figure S1.*19*). For the period of 1950 to 1975, our estimates are higher than the results by Cao et al., 2017, most likely because the starting year of their concrete stock estimations is 1931, while we traced cement consumption further back and started stock modelling in 1818. Values reported in Kapur et al., 2008 are almost identical to our estimate for the period 1900 to 1950. However, estimates from Cao et al., 2017 and Kapur et al., 2008 start to overtake the our results from 1970 and 1950 onwards, respectively. A possible explanation for this result is an increasing amount of downcycled cement/concrete EoL outflows. Kapur et al., 2008 and Cao et al., 2017 include those downcycled material flows as inflows of secondary material in their estimate of cement stocks, while we assume that actual recycling of concrete was low and that downcycled concrete is rather used as aggregates in sub-base and base-course layers. Other discrepancies are likely the result of variations in assumed recycling rates and lifetimes. Concrete EoL outflows also agree well with estimates by EPA (U.S. EPA, 2015, 2016, 2018; Figure S1.*26*) in terms of size and development over time. Our results are slightly higher, likely because we include both portland and masonry cement in our estimations of concrete stocks.

Bricks and Stones and Container Glass/Flat Glass

Stock data is not available for bricks and container and flat glass, but U.S. EPA, 2015, 2016, 2020 have estimated EoL outflows and recycling flows for bricks and total glass. Overall our results agree well with these estimates in terms of size and development over time (Figure S1.*27*, Figure S1.*28*, Figure S1.*35*), which gives us confidence that also our stock results are robust.

Aggregates and Asphalt

To compare asphalt stocks with those reported in Miatto, Schandl, Wiedenhofer et al., 2017, we have transformed their results for bitumen stocks to asphalt by assuming that asphalt contains 95 % sand and gravel (S&G) and 5 % bitumen.[14] Additionally, we have calculated the stock of aggregates in sub-base and base-course layers in Miatto, Schandl, Wiedenhofer et al., 2017 by subtracting S&G needed for asphalt and concrete from total S&G stock per road km, multiplying them with total road length of the different road types. Overall, the development of asphalt and aggregates stocks is similar over time, but the results of Miatto, Schandl, Wiedenhofer et al., 2017 are substantially lower than our

[14] NAPA (2019c) has also estimated the asphalt in-use stock in roads and arrives at a total stock of 16.3 Gigatonnes (18 billion short tons). However, as they do not describe the methodology according to which this number is calculated (and the year for this value), we cannot explain the differences between their estimates and our results.

estimates (Figure S1.*20*-Figure S1.*21*). According to Miatto, Schandl, Wiedenhofer et al., 2017, the actual depth/width of road layers for many road types is unknown. At the same time, these are likely the roads with the highest material intensities and road length. Therefore, we assume that the bottom-up approach of Miatto, Schandl, Wiedenhofer et al., 2017 is prone to underestimate the stock of asphalt and aggregates.

Non-Metallic Minerals

Overall, non-metallic minerals stocks and final waste flows agree with the results in Fishman et al., 2014 for the period of 1930 to 1970 (Figure S1.*22*, Figure S1.*33*). However, from 1970 to 2005, their results start to diverge from our estimate. Fishman et al., 2014 use ew-MFA data by Gierlinger & Krausmann, 2012 to estimate stocks. Gierlinger & Krausmann, 2012 assume that for every tonne of asphalt consumed annually, demand for aggregates as filling material in roads is 0.5 tonnes. In this study we have assumed a higher (but over time declining) demand for aggregates per tonne of asphalt. Therefore, we tried to replicate the figures of Gierlinger & Krausmann, 2012 for the domestic material consumption (extraction plus imports minus exports) of sand and gravel for concrete and asphalt production and for aggregates used as filling material. To obtain sand and gravel for concrete, cement consumption was multiplied by the factor 6.1. To obtain sand and gravel for asphalt, bitumen consumption was multiplied by the factor 20. The asphalt estimate was further increased by 50 % to account for sand and gravel as filling material. Figure S1.*37* shows a comparison between estimated and reported figures for sand and gravel consumption provided by Gierlinger & Krausmann, 2012.We find that the estimated figures for sand and gravel consumption are substantially lower than the reported figures by Gierlinger & Krausmann, 2012. We cannot explain these differences. However, since Gierlinger & Krausmann, 2012 estimate a much higher consumption for sand and gravel than we do in this study and this gap is also widening from 1980 onwards, we assume that the differences in S&G between our study and Gierlinger and Krausmann 2012 is a major factor in explaining the overall difference in non-metallic mineral stocks (see also Figure S1.*38*) between Fishman et al., 2014 and our results from 1970 to 2005.

3.4 Fossil Energy Carriers Comparison

Plastics

Data on the stock of plastics in the USA is not available. However, U.S. EPA, 2020 have estimated plastics EoL outflows and EoL recycling flows, which agree quite well with our results in terms of size and development of flows over time (Figure S1.*29*, Figure S1.*36*). It appears that our methodology for estimating inputs to stock and other parameters (e.g. lifetimes, manufacturing losses) for plastics gives reasonable results for EoL outflows, which also gives us confidence in the robustness of our material stock estimates for plastics.

4 Figures and Tables

4.1 Data for Material Flows and Parameters

4.1.1 Material Flows

Table S1.1: Data sources used to compile time series of stock-building materials and material inputs to stock.

Stock-Building Materials (ew-MFA)	ew-MFA Code (Extraction)	Material (Primary Input to Stock)	Source (Production)	Source (Trade)	Notes
Industrial Roundwood	A.1.4.1	Solidwood	1800-1899: Bureau of the Census (1975; L98-112)<br>1900-2014: Kelly and Matos (2014)	1900-2014: Kelly and Matos (2014) (lumber, plywood and veneer, wood panel products, other industrial wood products)<br>1962-2014: UNSD (2019a) (Cork and Wood Manufactures, final goods)	Consumption data for 2015-2017 was estimated based on growth rates for industrial roundwood production from FAO (2019)
Industrial Roundwood	A.1.4.1	Paper and Paperboard	1800-1899: Extrapolation<br>1900-2014: Kelly and Matos (2014)<br>2015-2017: FAO (2019)	1800-1964: no data<br>1965-2014: Kelly and Matos (2014)<br>2015-2017: FAO (2019)	Recycled paper production was subtracted from total paper production from 1900-1964; Extrapolation of inflows before 1900 was performed by multiplying per capita consumption in 1900 with population data from Bolt et al. (2018)
Iron Ore	A.2.1	Iron/Steel	1800-1863: Extrapolation<br>1864-1899: Bureau of the Census (1975; P231-300)<br>1900-2015: Kelly and Matos (2014)<br>2016-2017: USGS (2018)	1800-1912: no data, set to zero<br>1913-1961: Kelly and Matos (2014)<br>1962-2017: UNSD (2019a)	Secondary iron/steel production was subtracted from total iron/steel production from 1900-2017; Extrapolation of inflows before 1864 was performed by multiplying per capita consumption in 1900 with population data from Bolt et al. (2018)
Bauxite	A.2.2	Aluminum	1800-1885: no production<br>1886-1899: Bureau of the Census (1949; 152)<br>1900-2015: Kelly and Matos (2014)<br>2016-2017: USGS (2018)	1800-1910: no data/no trade<br>1911-1961: Kelly and Matos (2014)<br>1962-2017: UNSD (2019a)	Before 1886 aluminum production and trade was non-existent (Aluminum Leader, 2018)
Copper Ore	A.2.3.1	Copper	1800-1844: no data/set to zero<br>1845-1899: Bureau of<br>1900-2015: Kelly and Matos (2014)<br>2016-2017: USGS (2018)	1800-1869: no data/set to zero<br>1870-1899: Gierlinger and Krausmann (2012)<br>1900-1961: Kelly and Matos (2014)<br>1962-2017: UNSD (2019a)	Before 1844 copper production was very small (below 100 t/yr) and thus set to zero.
Other Metal Ores	A.2.3.2-A.2.3.8	Other Metals	1800-1899: Bureau of the Census (1974; M221-255)<br>1900-2015: Kelly and Matos (2014)<br>2016-2017: USGS (2018)	1800-1899: Bureau of the Census (1974; M221-255)<br>1900-2015: Kelly and Matos (2014)<br>2016-2017: USGS (2018)	Data for 1800-1899 includes manganese, lead and zinc.

Clays/Stones	A.3.1/A.3.5	Bricks and Stones	1800-1868: Extrapolation<br>1869-1970: Bureau of the Census (1975; P231-300)<br>1900-2015: Kelly and Matos (2014)<br>2016-2017: USGS (2019)	1800-1899: no data<br>1900-2015: Kelly and Matos (2014) (Stones)<br>1962-2017: UNSD (2019a) (Bricks)	Extrapolation of inflows before 1900 was performed by multiplying per capita consumption in 1900 with population data from Bolt et al. (2018)
Limestone, Clays (for Cement) + Sand and Gravel	A.3.1 + A.3.2 + A.3.4	Concrete	1800-1817: set to 0<br>1818-1899: Bureau of the Census (1975; M188-204)<br>1900-2015: Kelly and Matos (2014)<br>2016-2017: USGS (2018)	1800-1899: no data<br>1900-2015: Kelly and Matos (2014)<br>2016-2017: USGS (2018)	Data is for cement production and trade; concrete was estimated based on cement consumption and figures on share of cement in concrete from Cochran and Townsend (2010)
Limestone + Silica Sands + Soda Ash	A.3.2 + A.3.4 + A.3.8	Flat Glass	1800-1899: Extrapolation<br>1900-2015: Kelly and Matos (2014)<br>2016-2017: USGS (2018)	1900-2015: Kelly and Matos (2014) (soda ash)<br>2016-2017: USGS (2018) (soda ash)<br>1962-2017: UNSD (2019a) (flat glass)	All data except trade data for 1962-2017 (intermediate and final glass trade) is for soda ash production and trade, flat glass consumption was estimated based on soda ash consumption and coefficients from Ruth and Dell'Anno (1997); Extrapolation of inflows before 1900 was performed by multiplying per capita consumption in 1900 with population data from Bolt et al. (2018)
Limestone + Silica Sands + Soda Ash + Feldspar	A.3.2 + A.3.4 + A.3.8. + A.3.8	Container Glass	1800-1899: Extrapolation<br>1900-2015: Kelly and Matos (2014)<br>2016-2017: USGS (2018)	1900-2015: Kelly and Matos (2014)<br>2016-2017: USGS (2018)	Data is for soda ash production and trade, container glass consumption was estimated based on soda ash consumption and coefficients from Ruth and Dell'Anno (1997); Extrapolation of inflows before 1900 was performed by multiplying per capita consumption in 1900 with population data from Bolt et al. (2018)
Sand and Gravel	A.3.4	Aggregates for sub-base and base-course layers	Estimated with multiplier	Estimated with multiplier	Multiplier is based on Miatto et al. (2017a,c), see main text and SI.2.3 for explanation
Sand and Gravel + Crude Oil (Bitumen)	A.3.4 + A.4.3	Asphalt	1800-1869: set to 0<br>1870-1899: linear interpolation<br>1900-2013: Kelly and Matos (2014)<br>2014-2015: IEA (2019)<br>2016-2017: kept constant at 2015 level	1800-2013: no data<br>2014-2015: IEA (2019)<br>2016-2017: kept constant at 2015 level	Data is for bitumen production and trade, asphalt was estimated based on the assumption that asphalt contains 5% bitumen; no trade data for 1900-2013 because USGS only gives consumption
Crude Oil	A.4.3	Plastics	1800-1949: no data/no production<br>1950-2015: Geyer et al. (2017), Plastics Europe (2019), Bolt et al. (2018)<br>2016-2017: Extrapolation	1800-2017: no data	Consumption was estimated based on the displayed data sources; Extrapolation for 2016-2017 is based on average annual growth rate from 2010 to 2015

Notes: Aluminum/Concrete/Copper/Other Metals/Plastics consumption was very small/non-existent before 1886/1818/1845/1801/1950, respectively. For all other materials, we assumed constant per capita use prior to the earliest available year and multiplied per capita material flows with population data from Bolt et al., 2018 , except for Asphalt where we assume that production linearly increased from 0 tonnes in 1869 to 807,500 tonnes in 1905. For solidwood, lumber consumption data was available from 1800-2014 (2014-2017 estimated based on growth rates for Industrial Roundwood) while Other Industrial Wood Products were estimated before 1900. For Bricks/Stones, data was available for 1869-2017/1900-2015. Plastics Production in the USA was estimated for all years, based on global plastics production data by Geyer et al., 2017, production shares of the NAFTA region by Plastics Europe, 2019 and GDP data from Bolt et al., 2018. For details see SI.2.

4.1.2 Processing, Manufacturing and Construction Losses, Recycling and Downcycling

Table S1.2: Data sources for processing and manufacturing losses assumed in our model.

Material Inputs to Stock	Processing Losses	Manufacturing Losses	Sources (Processing Losses/Manufacturing Losses)
Solidwood	54 %	5 %	Cochran & Townsend, 2010; FAO, 2019; Kelly & Matos, 2014; Krausmann et al., 2018
Paper and Paperboard	54 %	0 %	Cochran & Townsend, 2010; FAO, 2019; Kelly & Matos, 2014; Krausmann et al., 2018
Iron (Steel)	58 %	2.2 %	Cullen et al., 2012; Krausmann et al., 2018
Aluminum	75 %	3.7 %	Chen & Graedel, 2012; Kelly & Matos, 2014; USGS, 2018
Copper	97.5-99.5 %	1 %	Gierlinger and Krausmann 2012; Wang et al. 2015; Glöser et al. 2013
Other Metals	94 %	2.3 %	Krausmann et al. 2017; Gerst and Graedel 2008; Kelly and Matos 2014
Bricks/Stones	26/0 %	4 %	Krausmann et al. 2017; Cochran and Townsend 2010
Concrete	42/0 %	3 %	Kapur et al. 2009; Krausmann et al. 2017; Cochran and Townsend 2010
Container Glass	15 %	0 %	Ruth and Dell´Anno 1997; Butler and Hooper 2011
Flat Glass	20 %	0 %	Ruth and Dell´Anno 1997; Butler and Hooper 2011
Sand and Gravel for sub-base and base-course Layers	0 %	0 %	Krausmann et al. 2017
Asphalt	0 %	0 %	Krausmann et al. 2017; Cochran and Townsend 2010
Plastics	0 %	9 %	Krausmann et al. 2017; U.S. EPA, 2020; Van Eygen et al. 2017

Notes: Processing losses for Solidwood and Paper and Paperboard are average processing losses for the period 1965-2014, processing losses for Aluminum are for Aluminum production from Bauxite and processing losses for Concrete are 42 % for Cement production and 0 % for Sand and Gravel.

Table S1.3: Data sources of downcycling rates.

Material	Downcycling rates	Sources	Comment
Bricks and Stones	1800-1920: 35 %<br><br>1931-1970: linearly decreasing from 35 % in 1921 to 10 % in 1970<br><br>1971-2017: linearly increasing from 10 % in 1970 to 47 % in 2017	Krausmann et al. (2017), USGS (2018, 2019)	Assumptions based on sources
Concrete	1800-1970: 10 %<br><br>1971-2017: linearly increasing from 10 % in 1970 to 55 % in 2017	Wilburn and Goonan (1998), Kelly (1998), Sandler (2003)	Assumptions based on sources
Asphalt	1800-1970: 10 %<br><br>1971-2017: linearly decreasing from 10 % in 1970 to 5 % in 2017	Wilburn and Goonan (1998), NAPA (2019b)	Assumptions based on sources

Table S1.4: Data sources of recycling flows and rates.

Material	Recycling Flows or Rates	Source and Information
Solidwood	No Reycling	Assumption based on Krausmann et al. (2017)
Paper and Paperboard	Flows	1800-1964: Share of recycled production in total production kept constant at level of 1965<br>1965-2014: Kelly and Matos (2014)<br>2015-2017: constant at 2014 level
Iron/Steel	Flows	1900-1938: linear interpolation from zero in 1900<br>1939-1997: Kelly and Matos (2014)<br>1998-2017: USGS (2019)
Aluminum	Flows	Before 1939: set to zero<br>1939-2015: Kelly and Matos (2014)<br>2016-2017: USGS (2018)
Copper	Flows	Before 1906: set to zero<br>1906-2015: Kelly and Matos (2014)<br>2016-2017: USGS (2018)
Other Metals	Flows	Before 1906: set to zero<br>1906-2015: Kelly and Matos (2014)<br>2016-2017: USGS (2018)
Bricks and Stones	Rates	From 15 % in 1800 linearly decreased to 0 % in 1960 (Krausmann et al., 2017)
Concrete	Rates	1970: 0 %, 1996: 3 %, 2017: 5 %; linear interpolation between these years (recycling rate set to 0 % before 1970); assumptions based on Krausmann et al. (2017), Kelly and Matos (2014), Kelly (1998), Wilburn and Goonan (1998)
Flat Glass	No Recycling	Assumption based on Wiedenhofer et al. (2019)
Container Glass	Rates	Data for the years 1960, 1970, 1980, 2990, 2005 and 2010-2017 from U.S. EPA (2020), linearly interpolated in between these years, recycling was set to 0 % before 1960.
Aggregates for sub-base and base-course layers	Rates	Constant at 60 %; assumption based on Wiedenhofer et al. (2019) and Krausmann et al. (2017)
Asphalt	Flows	Data for 1993 from USDT (1993), for 1996 from Wilburn and Goonan (1998) and 2009-2017 from NAPA (2019b), shares of recycled to new asphalt were calculated for available data points and linearly interpolated between them, starting from 0 % in 1970 to calculate recycling flows for all non-available years from 1970-2017.
Plastics	Rates	Data for the years 1980, 2990, 2005 and 2010-2017 from U.S. EPA (2020), linearly interpolated in between these years, recycling was set to 0 % before 1980.

4.1.3 Lifetimes

Table S1.5: End-use shares, lifetimes for end-uses and weighted lifetimes in 2017 and their data sources for material inputs to stock used in our model.

Material Inputs to Stock	Application (mean lifetime in years)	Lifetimes 1870 (years)	Lifetimes 2017 (years)	Sources (End-Use Shares/Lifetimes)
Solidwood	Construction: Lumber and Plywood/Veneer (75), Other: Lumber, Wood Panel Products and Other Industrial Wood Products (25)	50±5	63±7	Bureau of the Census 1975; Kelly and Matos 2014; U.S. EPA, 2015; Cochran and Townsend 2010
Paper and Paperboard	Printing and Writing Papers (9), Other: e.g. Newspapers, Household Sanitary Papers (1)	3±0.3	3±1	FAO, 2019; Penman, 2003
Iron/Steel	Construction (75), Containers (1), Other (23), Service Center and Distributors (30), Transportation (23)	29±3	34±4	Kelly and Matos 2014; USGS 2019; Müller et al. 2011
Aluminum	Construction (55), Consumer Durables (15), Containers and Packaging (1), Electrical (40), Machinery and Equipment (25), Transportation (20), Other (12)	31±4	22±3	Chen and Graedel 2012; Kelly and Matos 2014; USGS, 2019; Chen 2013
Copper	On-Site Waste (1), Plumbing (55), Wiring (45), Built-in Appliances (20), Industrial Electric and Electronic Products (20), Consumer Electric and Electronic Products (13), Infrastructure (65), Motor Vehicles (13), Other Transport (30)	39±8	34±7	Spatari et al. 2005; Kelly and Matos 2014; USGS 2019
Other Metals	Various Applications (Alloys, Batteries, ...): Other Metals are treated as one category where lifetimes are the average of the lifetimes of iron/steel, aluminum and copper	33±5	30±5	Single Use-Category/Gerst and Graedel 2008; Kelly and Matos 2014; Wiedenhofer et al. 2019
Bricks, Stones	Bricks and Stones for Construction (75)	75±8	75±8	Bureau of the Census 1975; Kelly and Matos 2014; Cochran and Townsend 2010
Concrete	Buildings (75), Roads/Bridges (32), Other (35)	46±5	52±6	Cochran and Townsend 2010
Flat Glass	Flat Glass applications (e.g. Windows, Glass Doors, …) (30-50)	50±5	30±3	Single Use-Category/Wiedenhofer et al. 2019
Container Glass	Container Glass applications (e.g. Bottles, Jars, …) (1.5-5)	5±1	3±0.3	Single Use-Category/Wiedenhofer et al. 2019
Sand and Gravel for sub-base and base-course layers	Buildings, Roads, Infrastructures (80)	80±24	80±24	Single Use-Category/Wiedenhofer et al. 2019
Asphalt	Roads (23)	35±4	35±4	Single Use-Category/Cochran and Townsend 2010
Plastics	Packaging (1), Building and Construction (35), Automotive (14-17), Electrical and Electronical (8), Textiles (5), Other (8)	11±2	10±2	Geyer et al. 2017; Plastics Europe 2019; Bento et al. 2016

Notes: Lifetimes are shown with a mean (left value) and tone standard deviation (right value). For 2000-2017, lifetimes of Spatari et al. 2005 were allocated to end-use shares by Kelly and Matos 2014/USGS 2019 as follows: Building Construction: On-Site Waste, Plumbing, Wiring, Built-in Appliances (average lifetimes, assuming constant shares of 1999); Infrastructure: Electrical and Electronic Products; Industrial Electric and Electronic Products: Industrial Machinery and Equipment; Motor Vehicles and other Transport: Transportation Equipment (average lifetimes, assuming constant shares of 1999); Consumer Electric and Electronic Products: Consumer and general Products.

4.1.4 Lifetimes

Figure S1.1: Lifetimes used in the MISO-model, 1800-2017 (for sources see Table S1.5)

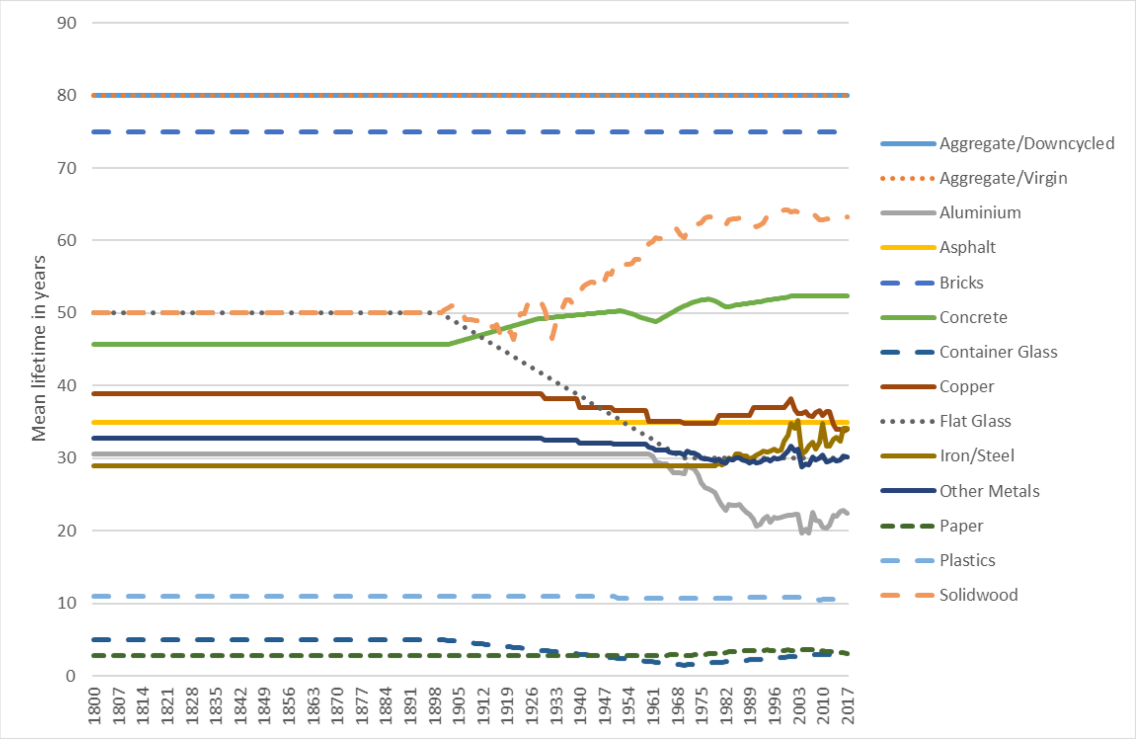

4.1.4 Recycling and Downcycling

Figure S1.2: End-of-Life recycling rates used in the MISO-model, 1800-2017

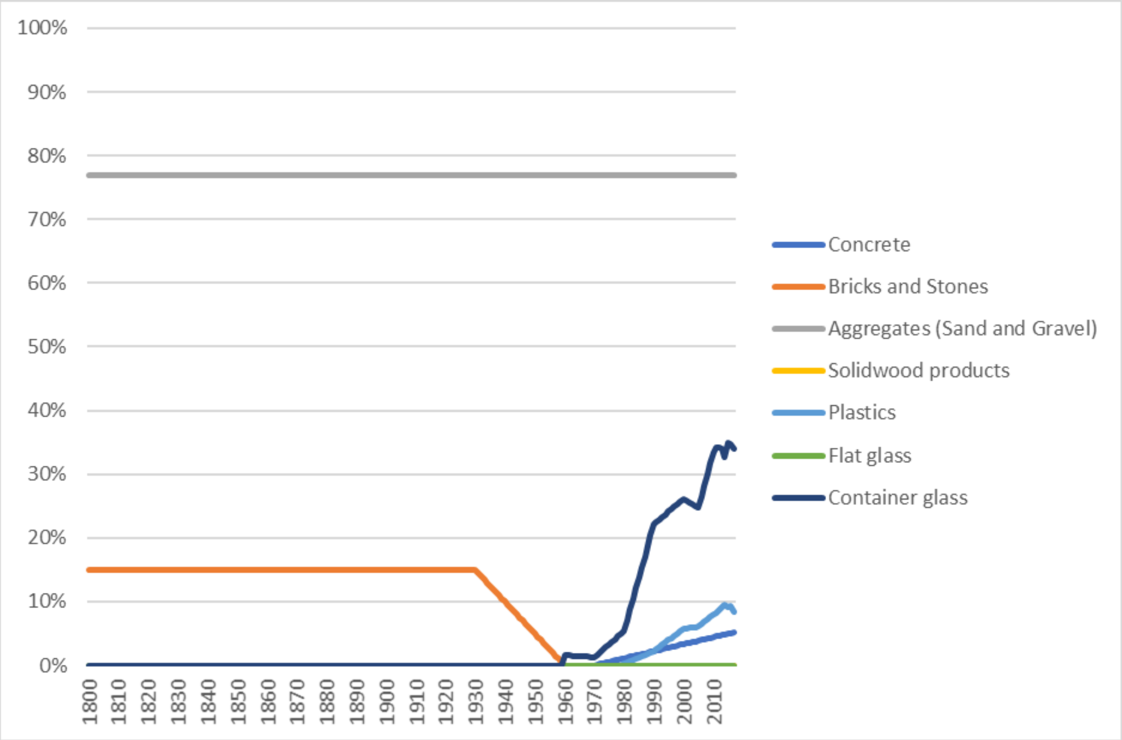

Sources: Wilburn and Goonan 1998, Kelly 1998, Sandler 2003, Krausmann et al. 2017, U.S. EPA, 2018, Wiedenhofer et al. 2019, own calculations (see Supporting Information S2).

Figure S1.3: End-of-Life recycling flows used in the MISO-model, 1800-2017 (for sources Table S1.4)

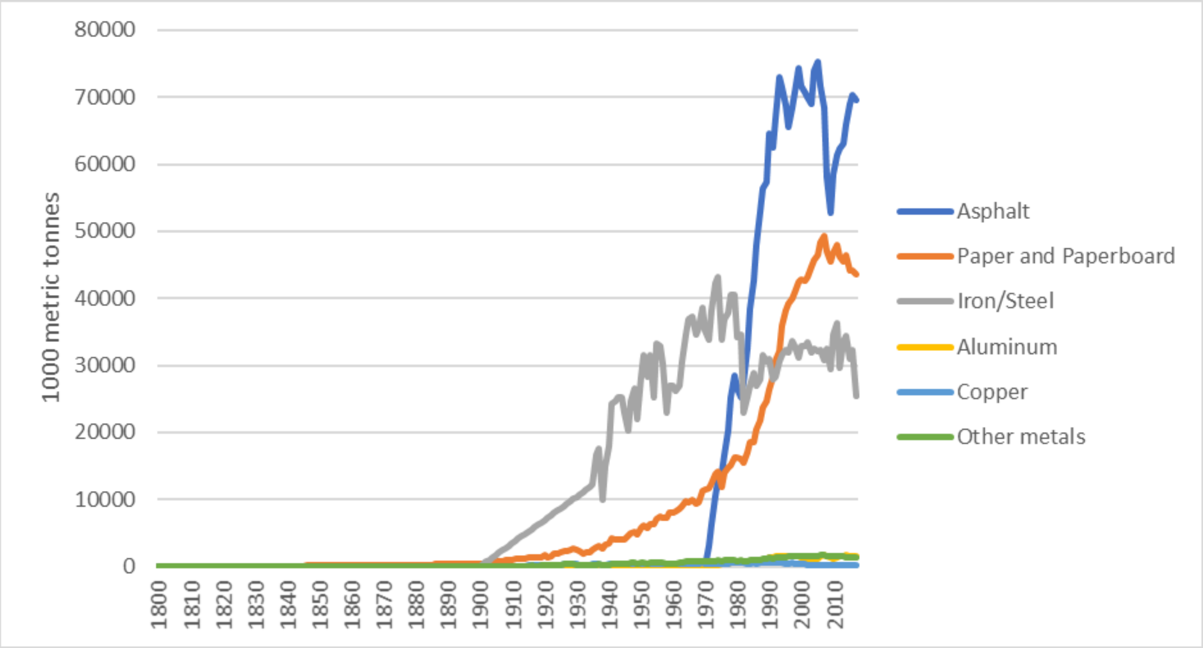

Sources: USDT 1993, Wernick et al. 1996, Wilburn and Goonan 1998, Brown 2013, Kelly and Matos 2014, FHA 2016, NAPA, 2019a, own calculations (see Supporting Information S2).

Figure S1.4: End-of-Life downcycling rates used in the MISO-model, 1800-2017 (for sources Table S1.4)

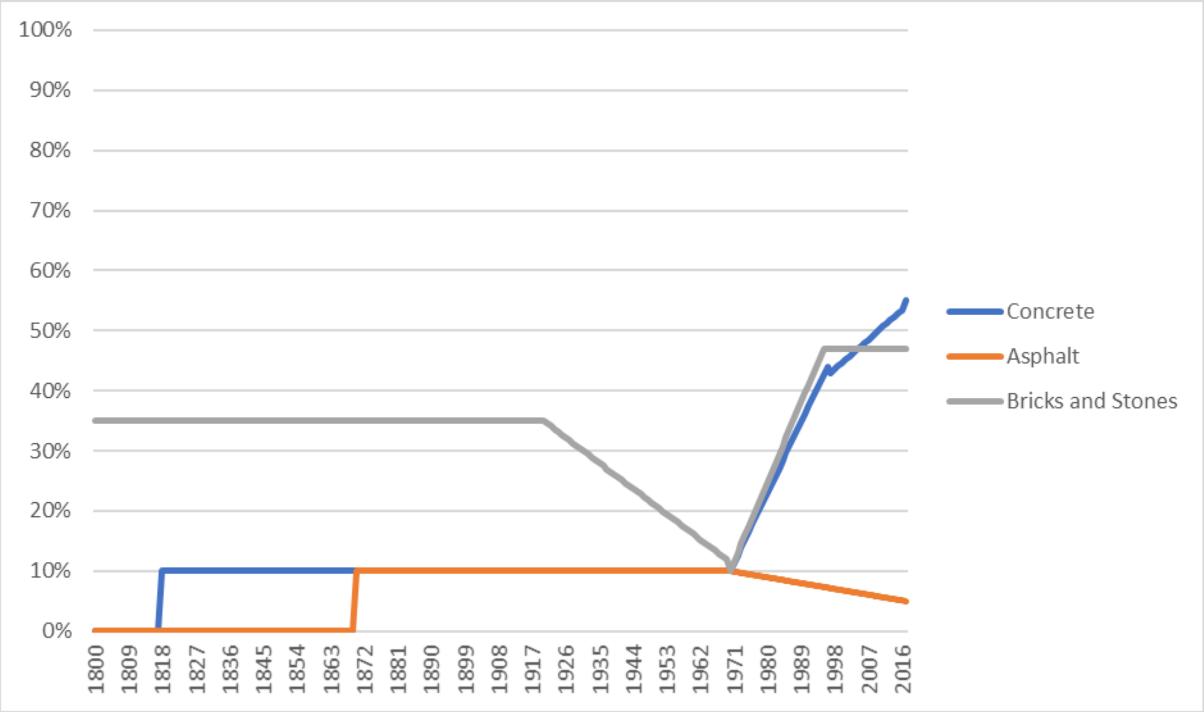

Sources: Wilburn and Goonan 1998, Sandler 2003, Krausmann et al. 2017, NAPA, 2019a, Wiedenhofer et al. 2019, own calculations

4.1.6 Multipliers

Table S1.6: Material multipliers for asphaltic road types

	Depth (m)	Length (m)	Width (m)	Density (t per m^3)	Asphalt (t)	Sand and Gravel (t)	Multiplier
Low Type pavement	0.02/0.05	1000	3.75	2.24/2.19	168	410	2.4
Intermediate pavement	0.035/0.135	1000	6	2.24/2.19	470.4	1770	3.8
High flexible pavement	0.05/0.21	1000	10.5	2.24/2.19	1176	4818	4.1
High composite pavement	0.05/0.03	1000	12	2.24/2.19	1344	787	0.6

Notes: Depth/length/width of roads is based on (Miatto, Schandl, Wiedenhofer et al., 2017), densities are based on Krausmann et al. (2018). Width for low type pavement/high flexible pavement are the averages for combined rural and urban roads in (Miatto, Schandl, Wiedenhofer et al., 2017). The left value in the depth column is for asphalt, the right value in the depth column is for sand and gravel. The amount of asphalt (in tonnes) and sand and gravel (in tonnes) needed per road kilometre is equal to depth*length*width*density. The multiplier is equal to sand and gravel in asphaltic road types divided by asphalt needed in asphaltic road types.

Figure S1.5: Total road kilometres for asphaltic road types in the USA, 1905-2015

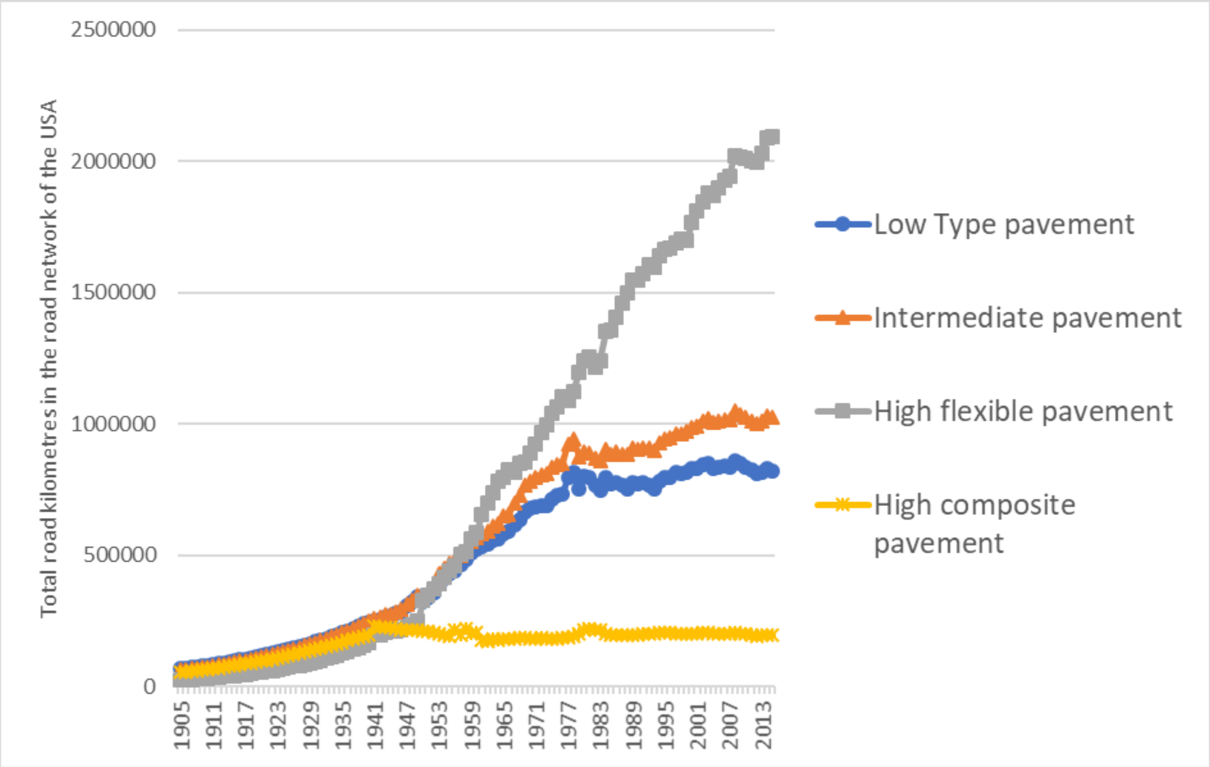

Source: Miatto, Schandl, Wiedenhofer et al., 2017, own calculations

Figure S1.6: Shares of road types in total new asphaltic road construction in the USA, 1905-1940

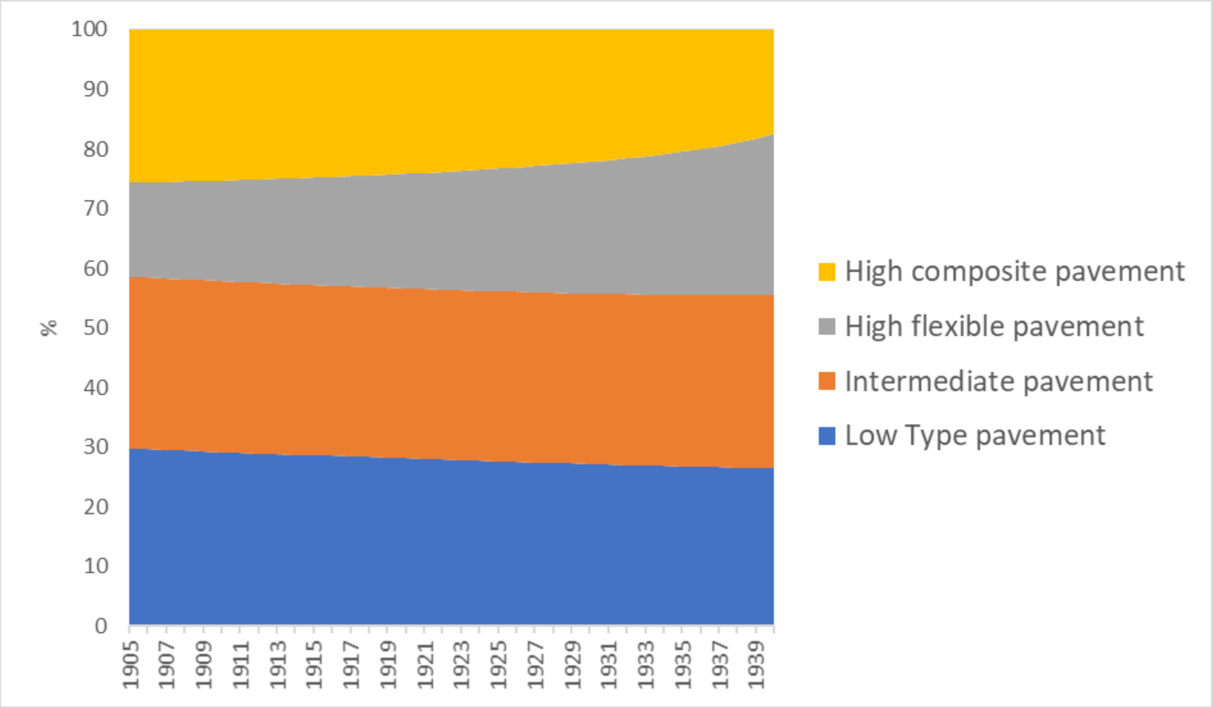

Source: Miatto, Schandl, Wiedenhofer et al., 2017, own calculations

Figure S1.7: Shares of road types in total new asphaltic road construction in the USA, 1941-1980

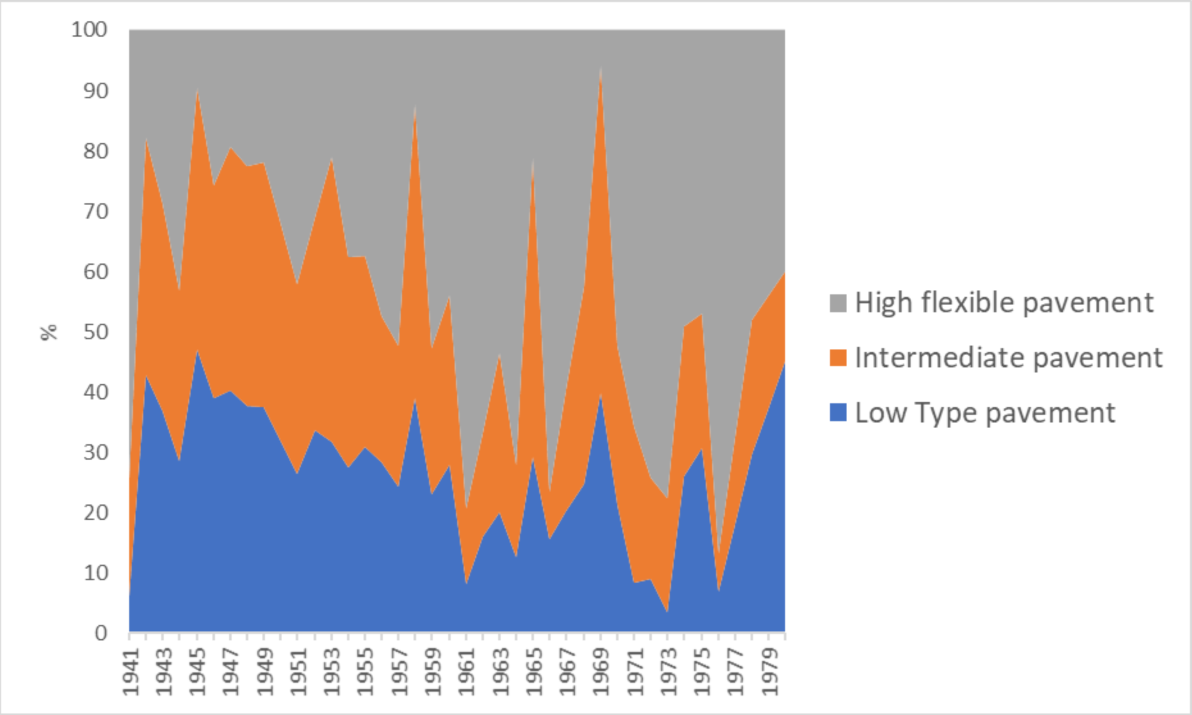

Source: Miatto, Schandl, Wiedenhofer et al., 2017, own calculations

Figure S1.8: Calculated average MultiplierTotal for asphaltic roads, 1870-2017

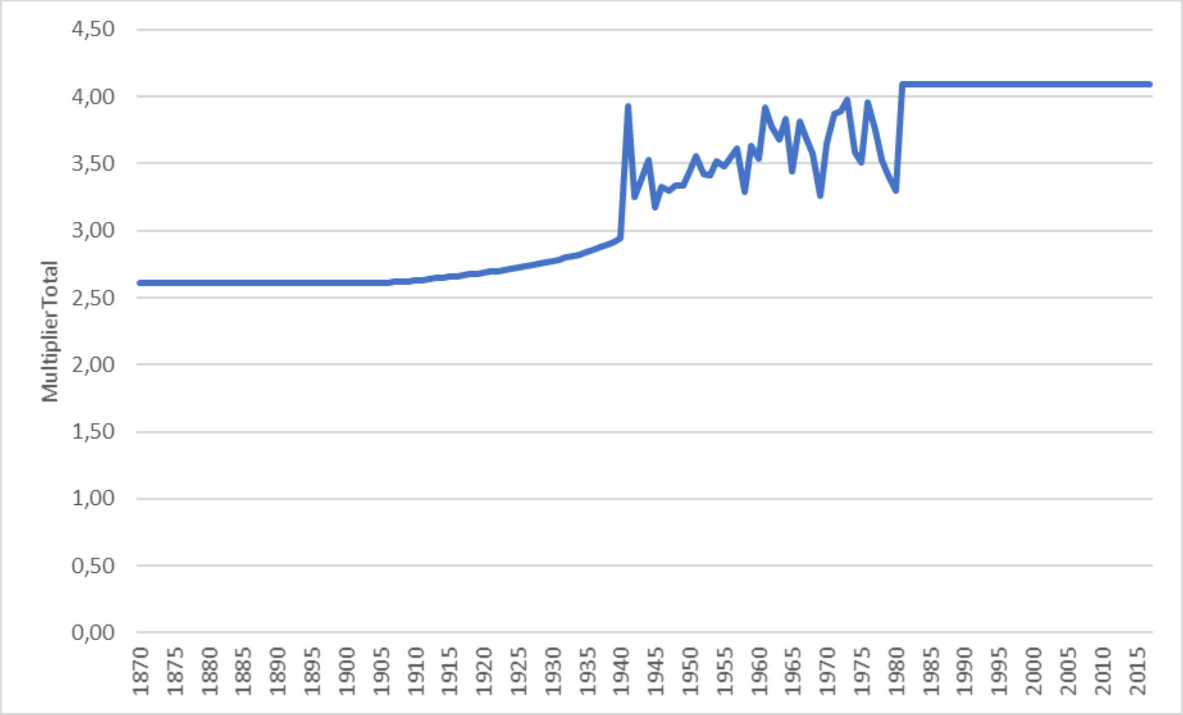

Source: Miatto, Schandl, Wiedenhofer et al., 2017, own calculations

Figure S1.9: Assumed share of asphalt used for new road construction in the USA, 1870-2017

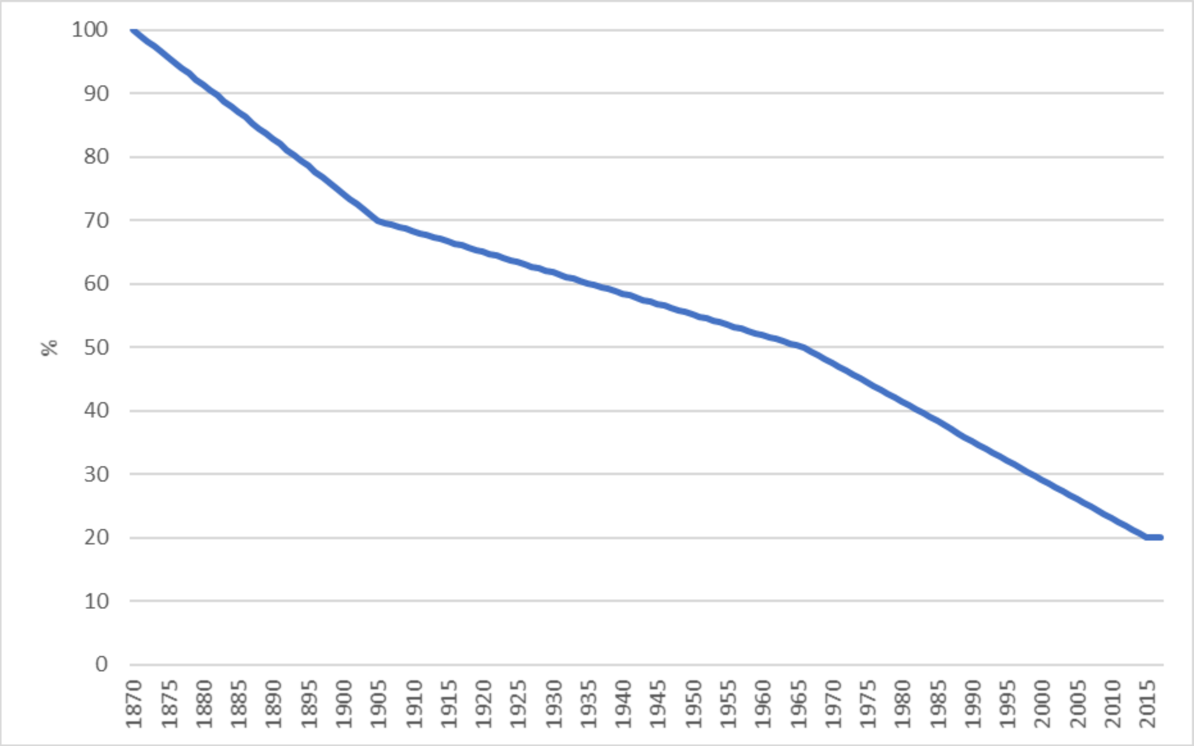

Source: Miatto et al. 2017b, own calculations

Figure S1.10: Multipliers for Sub-Base and Base-Course Layers 1800-2017

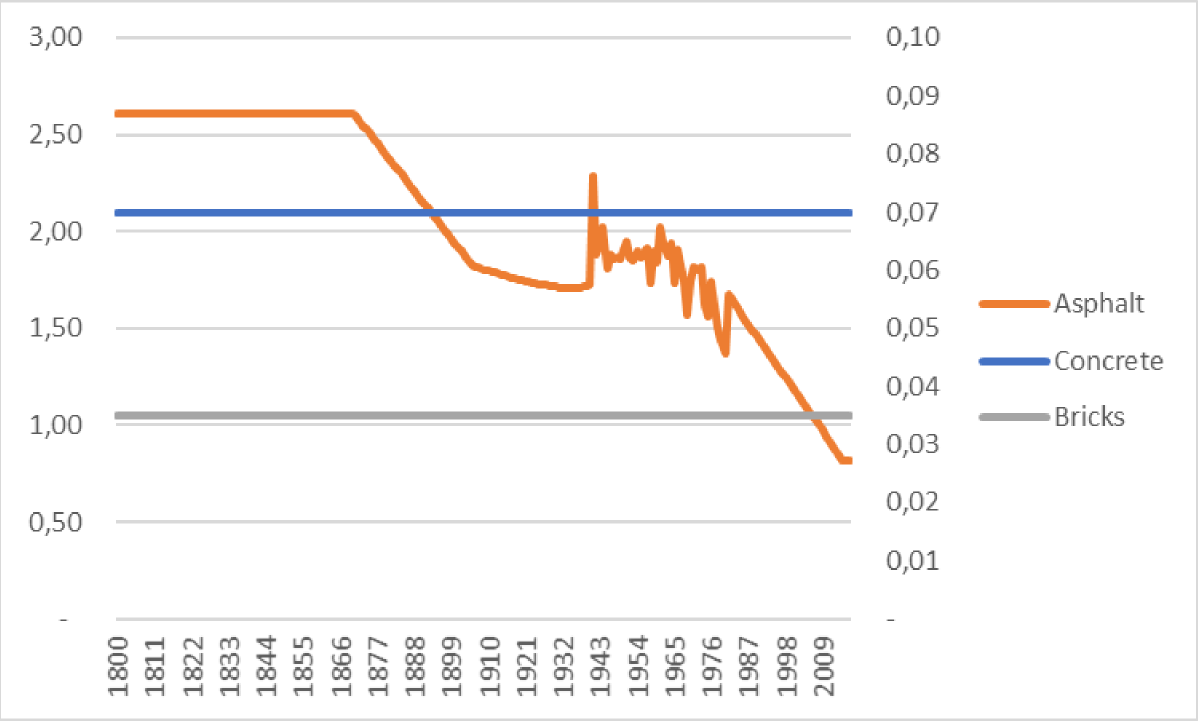

Source: Miatto, Schandl, Wiedenhofer et al., 2017, own calculations

4.2 Comparison with Results of other Studies

4.2.1 Material Stocks

Figure S1.11: Comparison of biomass stock estimates for USA for 1930-2005

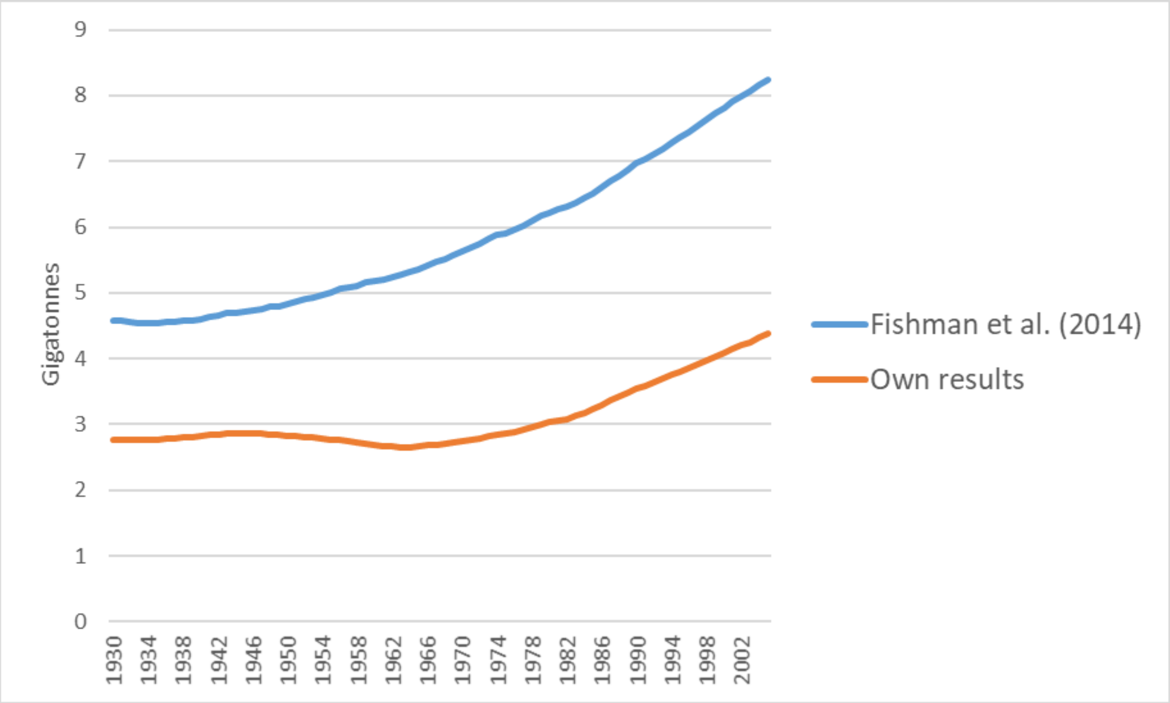

Notes: Own results include stocks for solidwood and paper and paperboard. Stocks estimates from Fishman et al. 2014 include timber.

Figure S1.12: Development of per capita stocks for iron/steel in the USA, 1900-2005 according our results

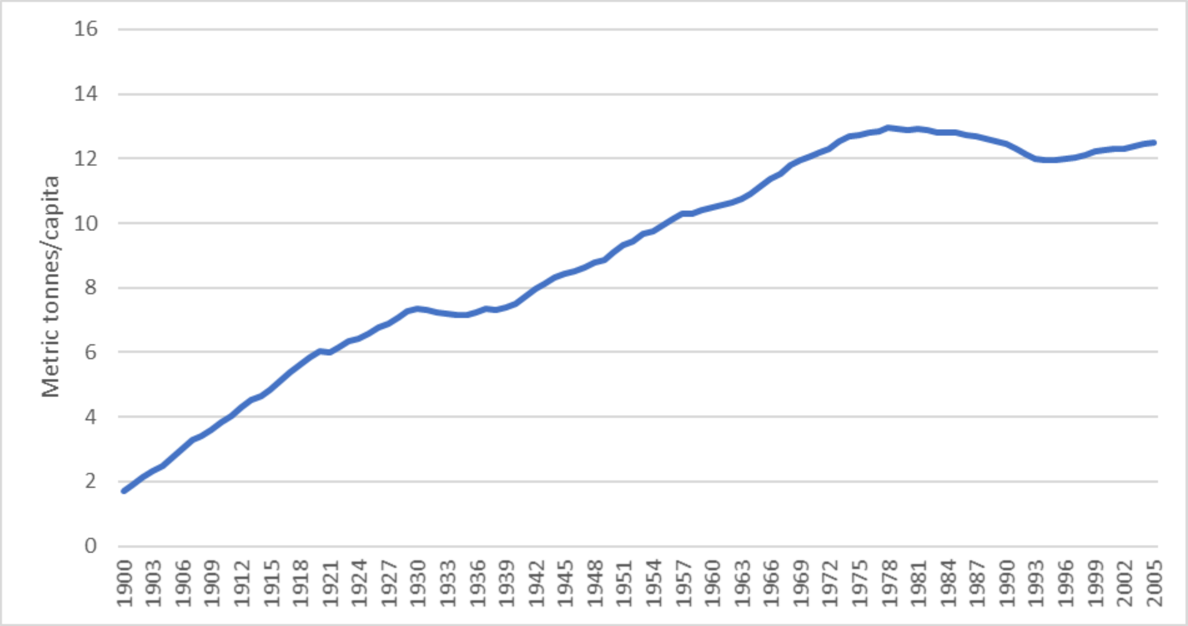

Notes: Absolute stock results are from own calculations and were divided by population data from Bolt et al., 2018 to obtain per capita stocks.

Figure S1.13: Comparison of per capita stock estimates for iron/steel in the USA for 1900-2008

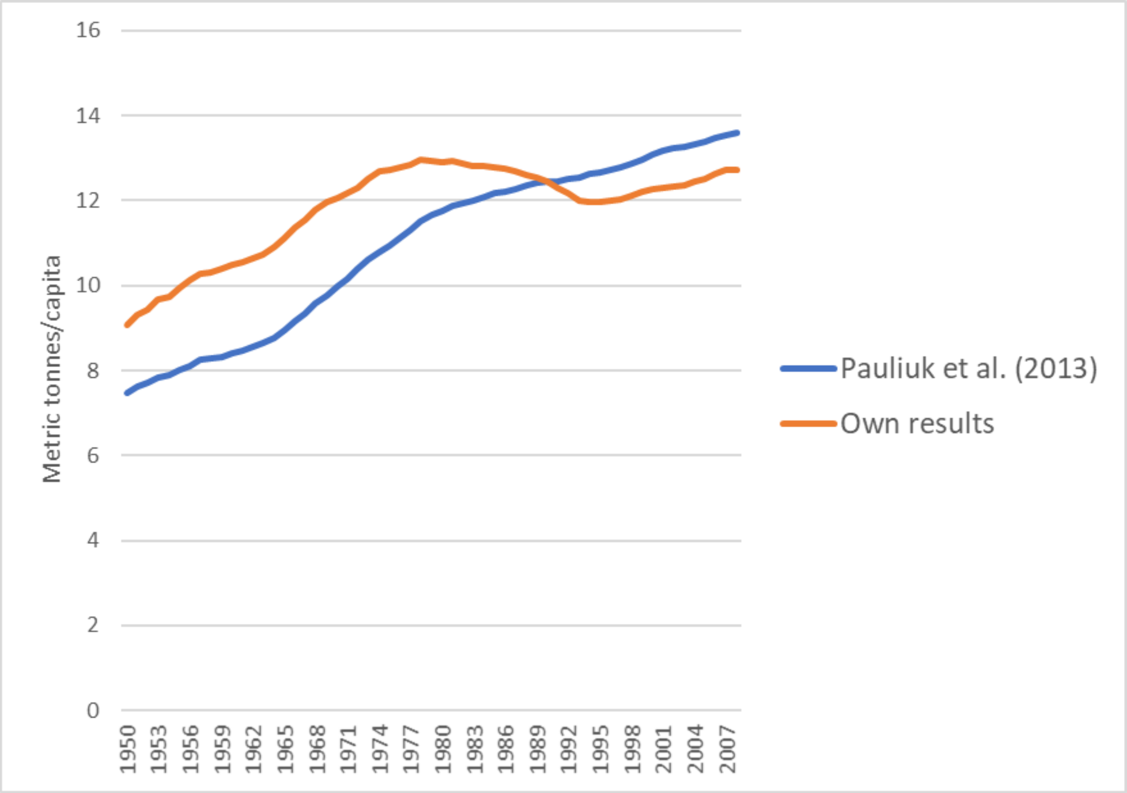

Notes: Absolute stock results are from own calculations and divided by population data from Bolt et al., 2018 to obtain per capita stocks.

Figure S1.14: Comparison of stock estimates for iron/steel in the USA for 1930-2005

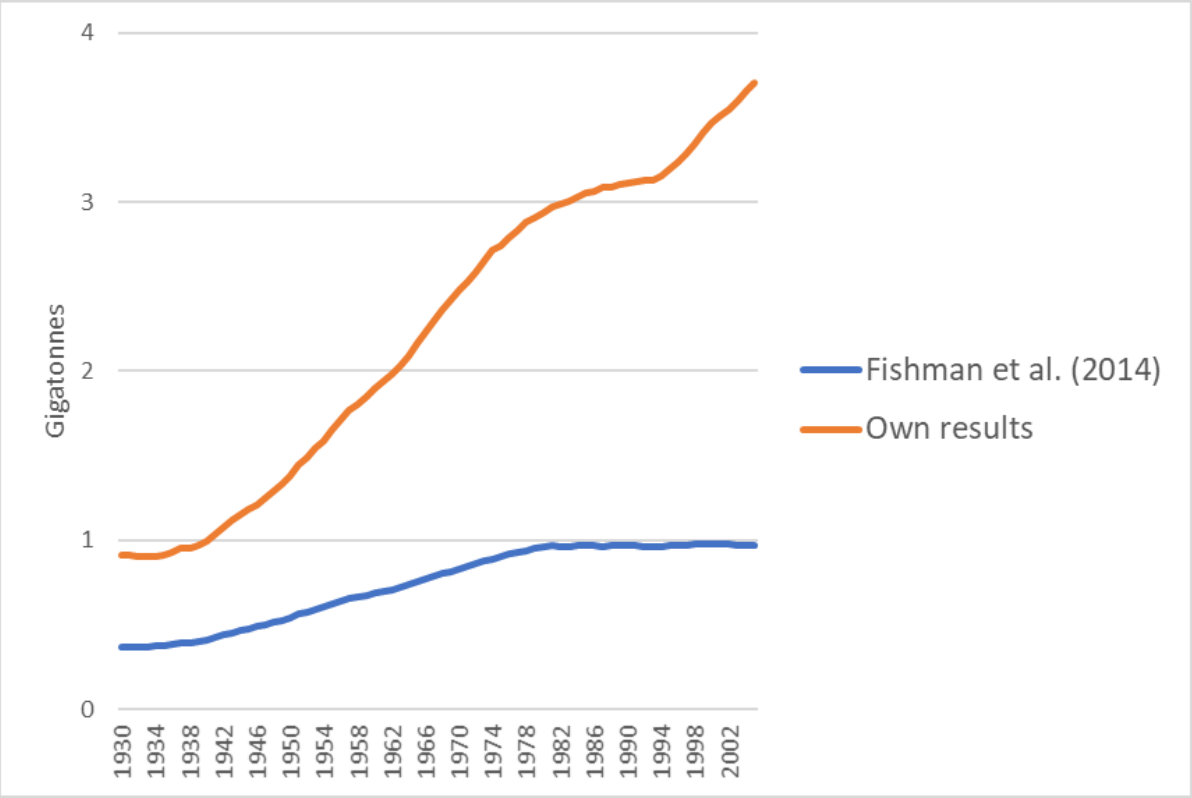

Notes: Own results include stocks for iron/steel. Stock estimates from Fishman et al. 2014 include iron.

Figure S1.15: Comparison of per capita stock estimates for aluminum in the USA for 1950-2008

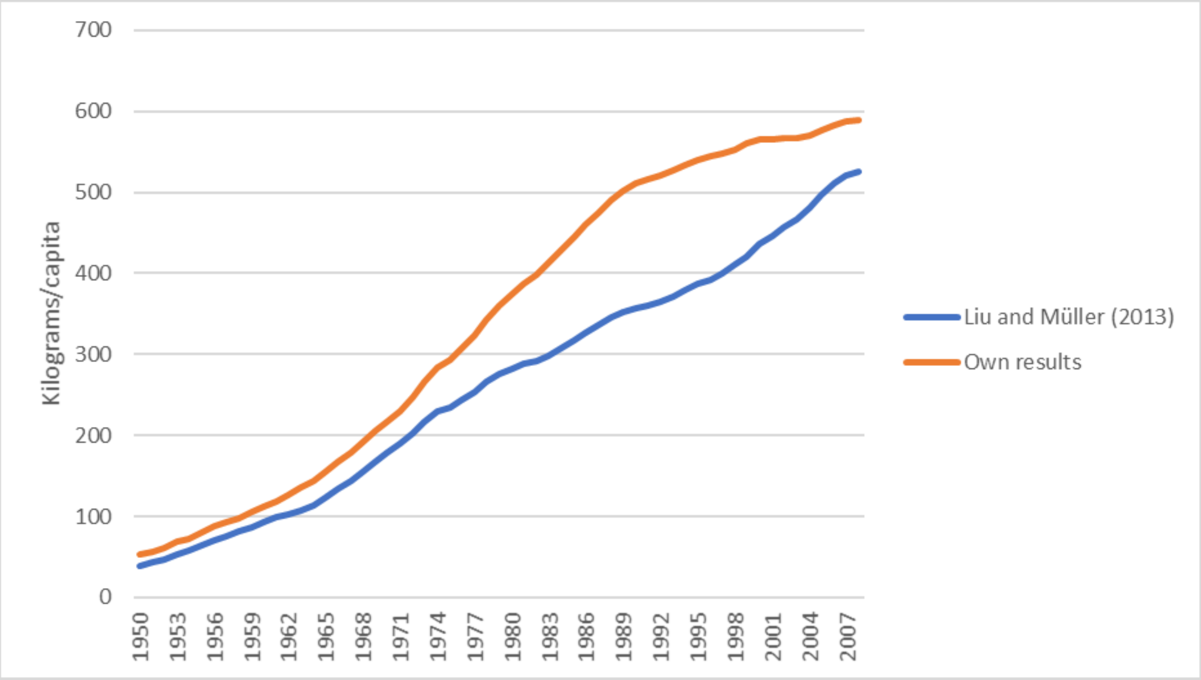

Note: Absolute stock results are from own calculations and divided by population data from Bolt et al., 2018 to obtain per capita stocks.

Figure S1.16: Own results for aluminum stocks in the USA, 1900-2010

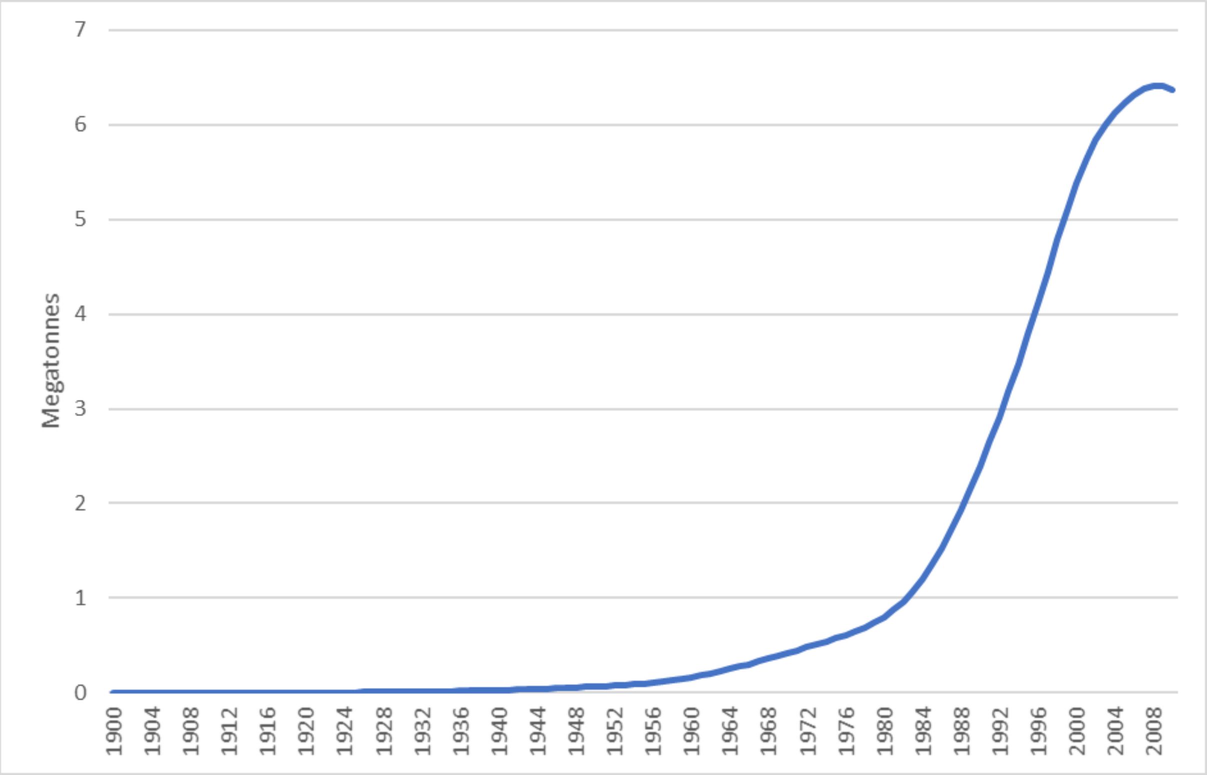

Notes: Estimates are own calculations

Figure S1.17: Comparison of estimates of stocks of non-ferrous metals in the USA for 1930-2005

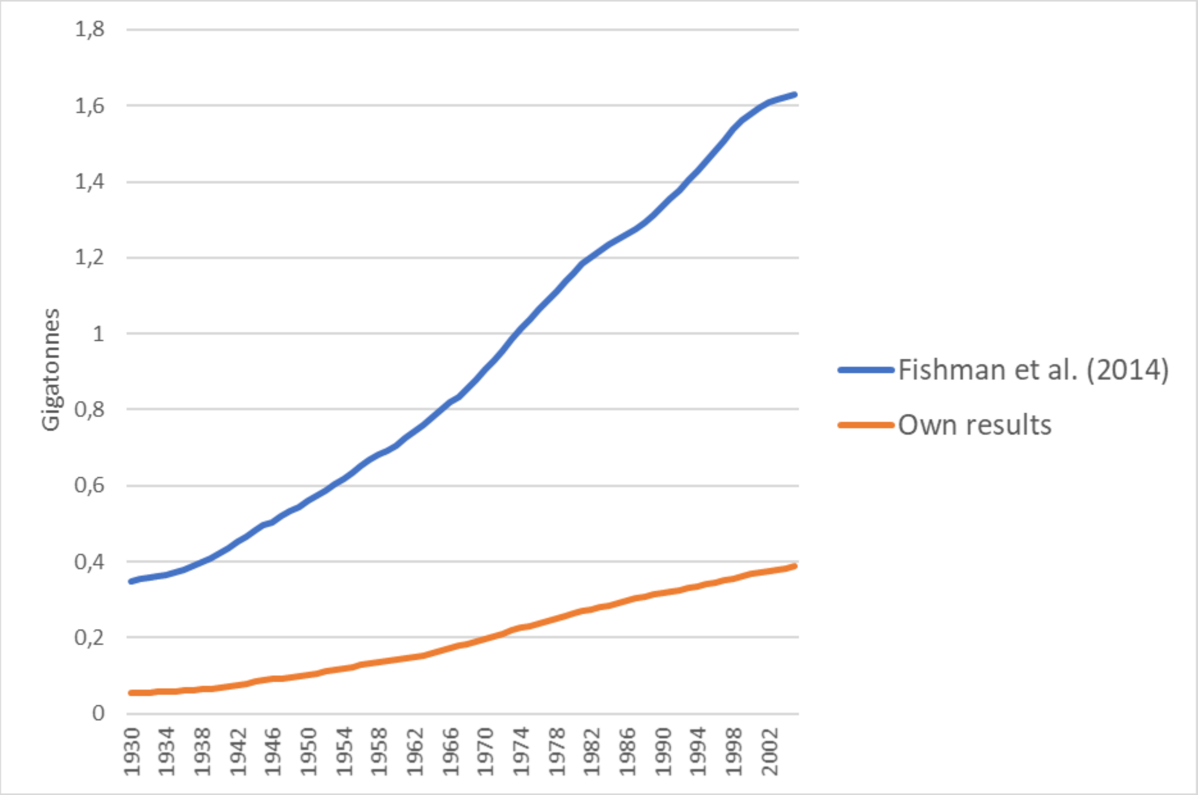

Notes: Own results include stocks for aluminum, copper and other metals. Stock estimates from Fishman et al. 2014 include all metals other than iron.

Figure S1.18: Comparison of estimates of concrete stocks in the USA for 1900-2005

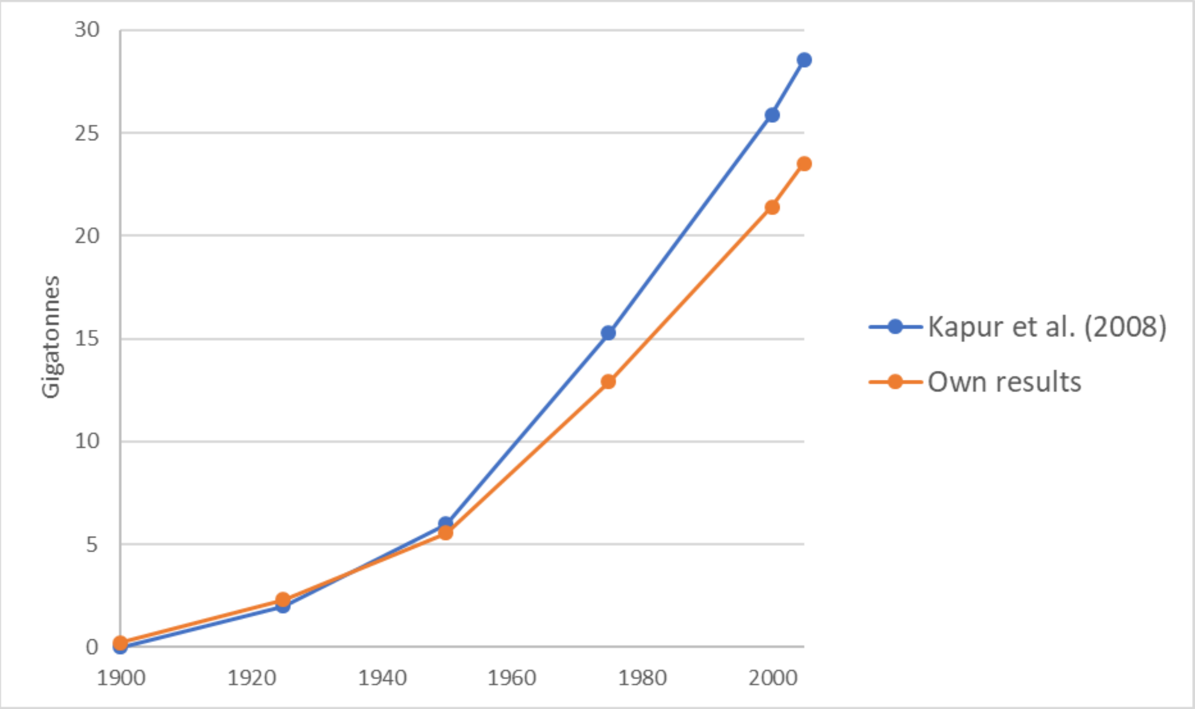

Notes: Values for Kapur et al. 2008 were calculated based on their results for cement in-use-stocks and coefficients from Cochran and Townsend 2010 which we also used to calculate concrete production values for own results (see SI.2.3).

Figure S1.19: Comparison of estimates of per capita stocks of concrete in the USA for 1950-2014

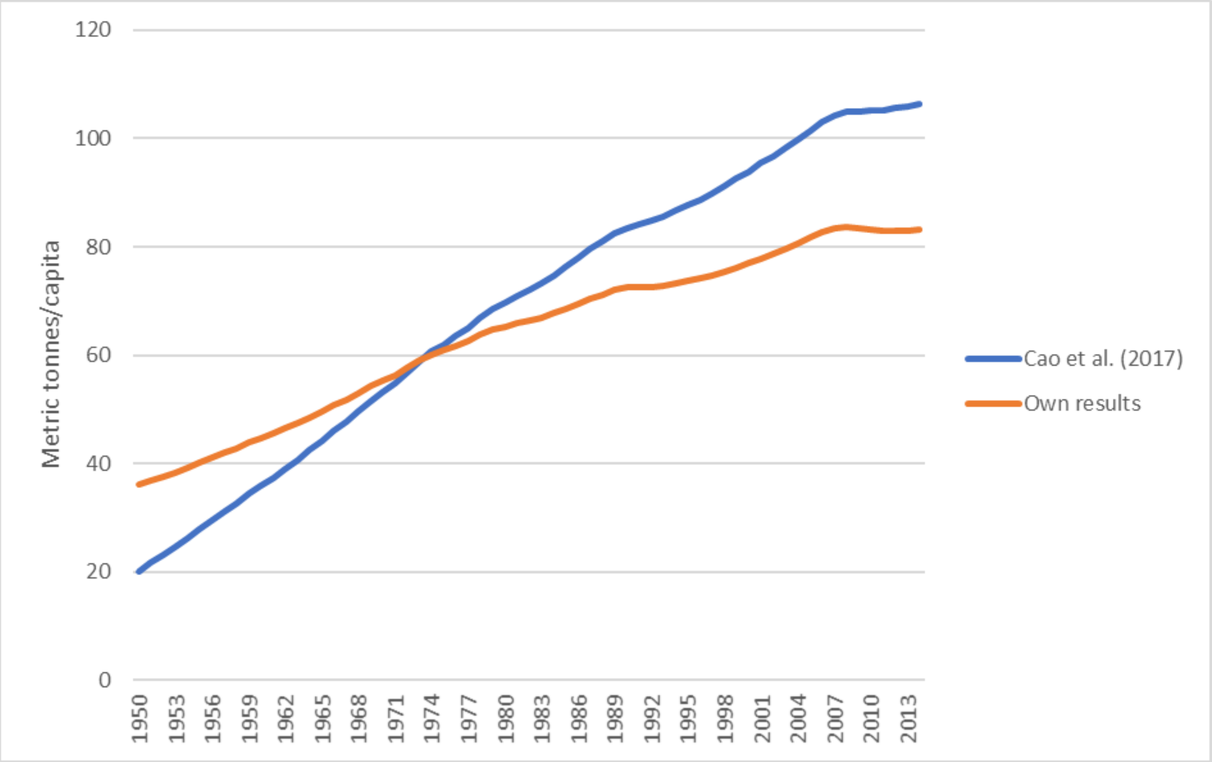

Notes: Values for Cao et al. 2017 were calculated based on their results for cement in-use-stocks and coefficients from Cochran and Townsend 2010 which we also used to calculate concrete production values used for own results (see SI.2.3). Own results were divided by population data from Bolt et al., 2018 to obtain per capita stocks.

Figure S1.20: Comparison of estimates of stocks of aggregates in sub-base and base-course layers in the USA for 1905-2015 (please watch out for different axis).

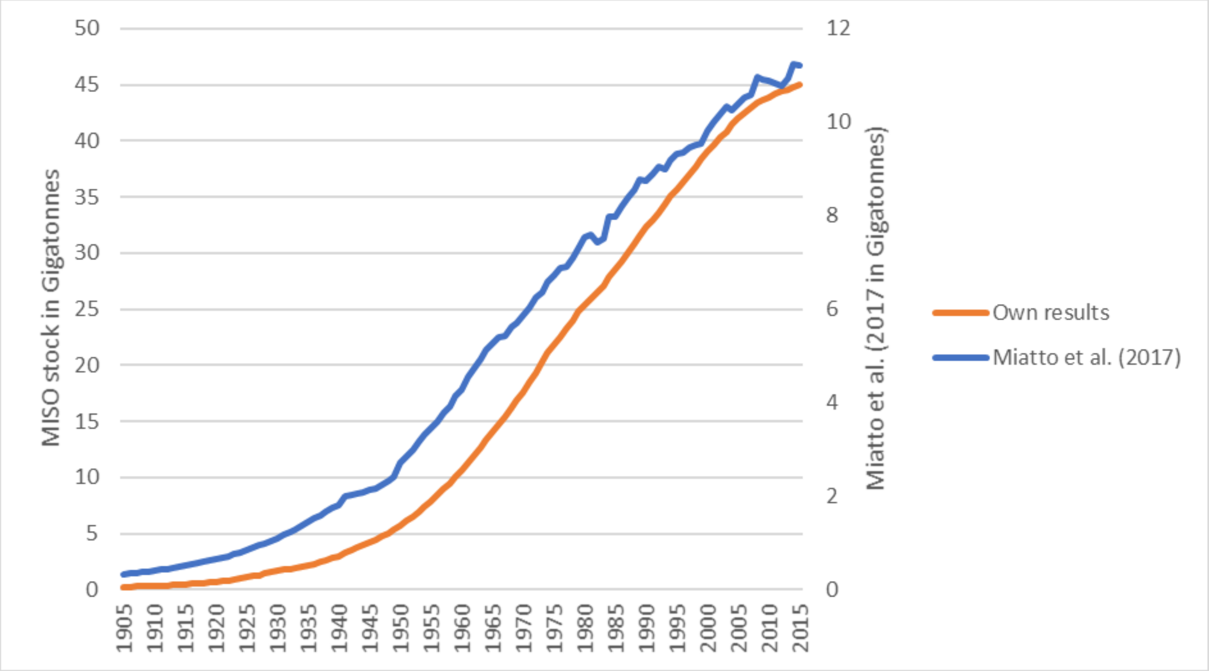

Note: Values for Miatto, Schandl, Wiedenhofer et al., 2017 include aggregates in sub-base and base-course layers of roads, Own results also include a small fraction of aggregates in sub-base and base-course layers of other infrastructure such as buildings. Own results are depicted on the left axis, the results of Miatto, Schandl, Wiedenhofer et al., 2017 are depicted on the right axis.

Figure S1.21: Comparison of estimates of stock of asphalt in the USA for 1905-2015 (please watch out for different axis).

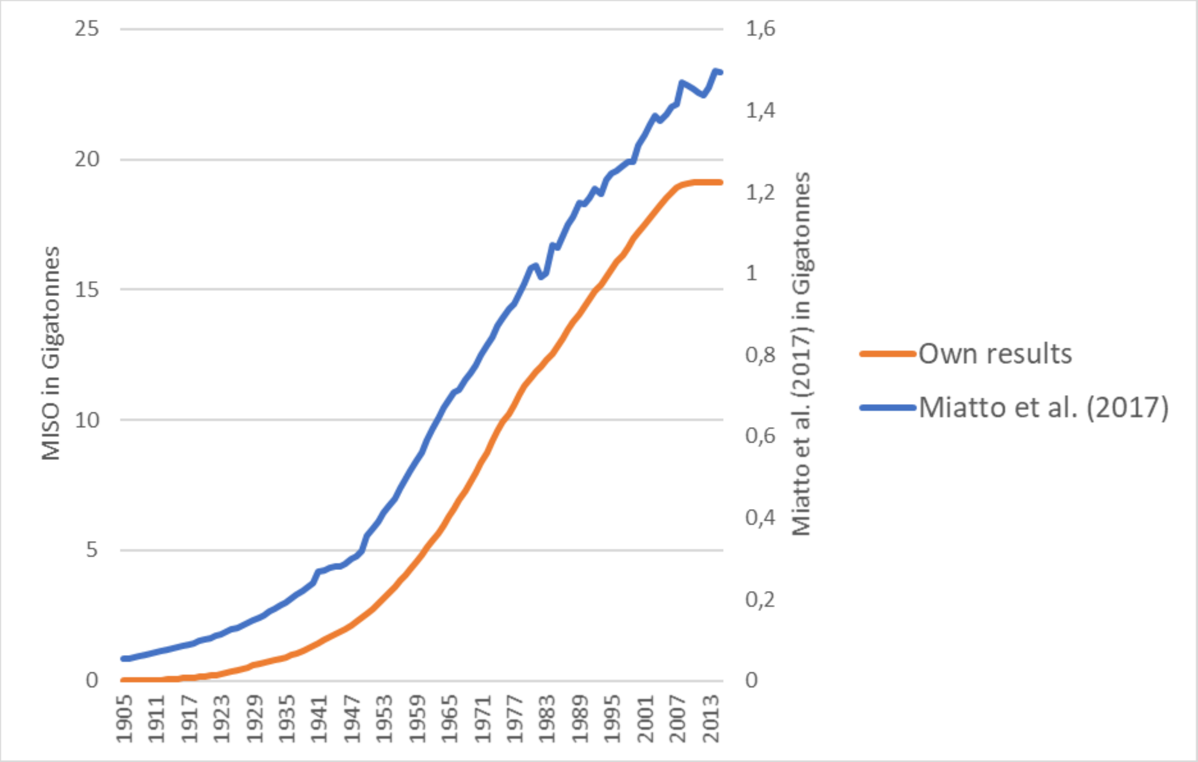

Note: Values for Miatto, Schandl, Wiedenhofer et al., 2017 were calculated based on their results for bitumen stocks and the assumption that asphalt consists of 95 % sand and gravel and 5 % bitumen. Own results are depicted on the left axis, the results of Miatto, Schandl, Wiedenhofer et al., 2017 are depicted on the right axis.

Figure S1.22: Comparison of estimates of stocks of non-metallic minerals in the USA for 1905-2015

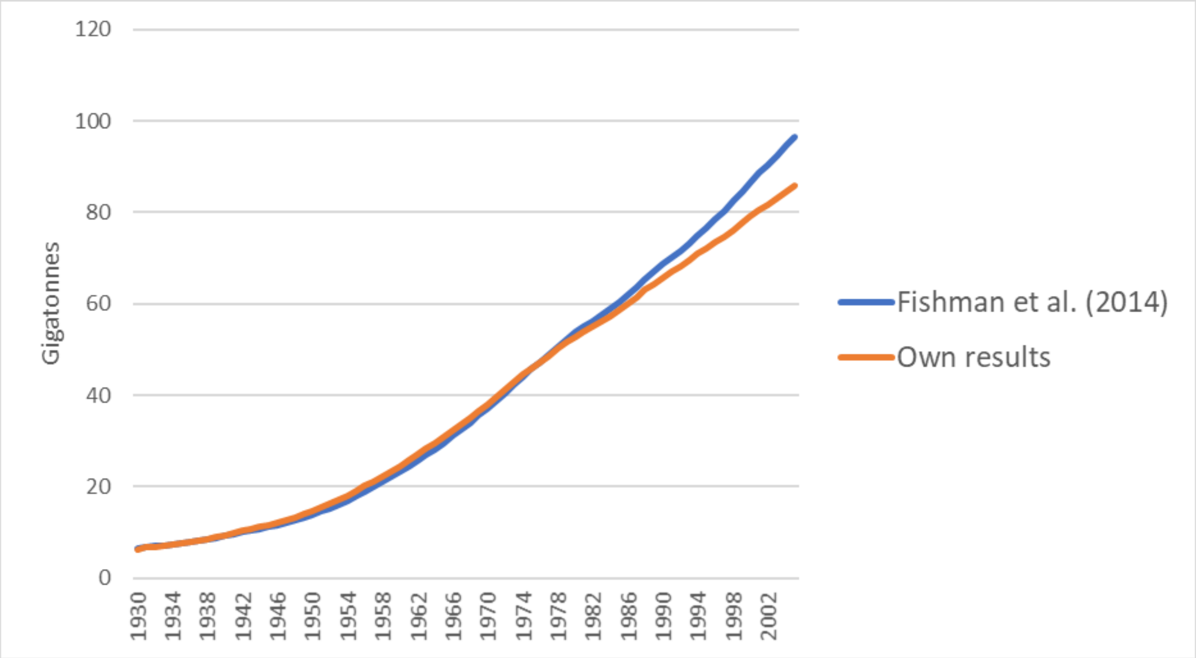

Notes: Own results include stocks for concrete, asphalt, aggregates in sub-base and base-course layers, bricks and stones, container glass and flat glass. Stock estimates from Fishman et al. 2014 include all non-metallic minerals.

Table S1.7: Comparison of estimates of stocks of iron/steel in the USA for various years

Author	Year	Estimate (Gt)	Own results (Gt)	% Difference
Brown, 1954*	1950	2,3	1.4	-39%
Sullivan, 2003*	2000	4,0	3.5	-14%
Rauch, 2009	2000	3,2	3.5	8%
Sullivan, 2005	2002	4,1	3.6	-14%
Müller et al., 2006	2004	3,1	3.7	19%
Müller et al., 2011	2005	3,2	3.7	16%

Notes: *Values were taken from Gerst and Graedel 2008; whenever per-capita stocks were depicted in the literature, they were multiplied with population data from Bolt et al., 2018 to obtain total stocks.

Table S1.8: Comparison of estimates of stocks of aluminum in the USA for various years

Author	Year	Estimate (Gt)	Own results (Gt)	% Difference
Liu & Müller, 2013	2000	0,12	0.16	29%
Sullivan, 2003**	2000	0,14	0.16	17%
Rauch, 2009	2000	0,11	0.16	47%
Sullivan, 2005*	2002	0,14	0.16	15%
Sullivan, 2005	2002	0,14	0.16	15%
Hatayama et al. 2009*	2003	0,12	0.17	38%
Liu & Müller, 2013	2005	0,15	0.17	16%
Liu et al., 2011*	2006	0,15	0.17	19%
McMillan et al., 2010***	2007	0,09	0.18	88%
Chen & Graedel, 2012	2009	0,15	0.18	20%
Liu & Müller, 2013	2010	0,16	0.18	11%

Notes: *Values were taken from Liu and Müller 2013 **Values were taken from Gerst and Graedel 2011 ***Average value of 0.911-0.976 was taken for McMillan et al. 2010; whenever per-capita stocks were depicted in the literature, they were multiplied with population data from the Bolt et al., 2018 to obtain total stocks.

Table S1.9: Comparison of stock estimates for copper in the USA for various years

Author	Year	Estimate (Gt)	Own results (Gt)	% Difference
Ingalls, 1935*	1932	0,01	0.02	110%
Merrill, 1949*	1948	0,02	0.03	64%
Merrill, 1949*	1957	0,03	0.04	48%
McMahon, 1965*	1960	0,04	0.04	10%
Merrill, 1949*	1961	0,05	0.04	-12%
Sousa, 1981*	1979	0,07	0.06	-6%
Jolly, 1993*	1990	0,07	0.07	6%
Zeltner et al., 1999*	1990	0,07	0.07	-2%
Ayres et al., 2002*	1998	0,07	0.08	15%
Gordon et al., 2006*	1999	0,07	0.08	23%
Sullivan, 2003*	2000	0,11	0.08	-24%
Rauch, 2009	2000	0,07	0.08	25%
Sullivan, 2005	2002	0,12	0.09	-26%
Nathan Associates, 2004*	2003	0,05	0.09	72%

Notes: *Values were taken from Gerst and Graedel 2008; whenever per-capita stocks were depicted in the literature, they were multiplied with population data from Bolt et al., 2018 to obtain total stocks.

4.2.2 End-of-Life Outflows

Figure S1.23: Comparison of estimates of End-of-Life outflows of paper and paperboard in the USA for 1905-2015

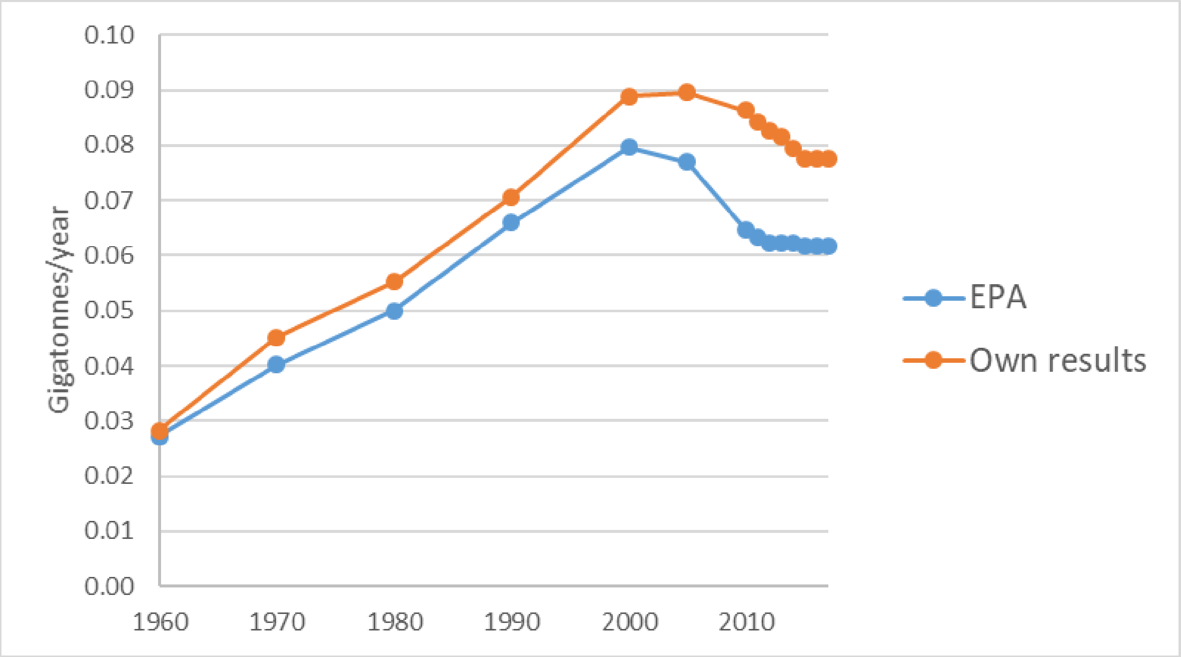

Note: We classified municipal solid waste of paper and paperboard from U.S. EPA, 2020 as End-of-Life outflows.

Figure S1.24: Comparison of estimates of End-of-Life outflows of solidwood in the USA for 2012-2015

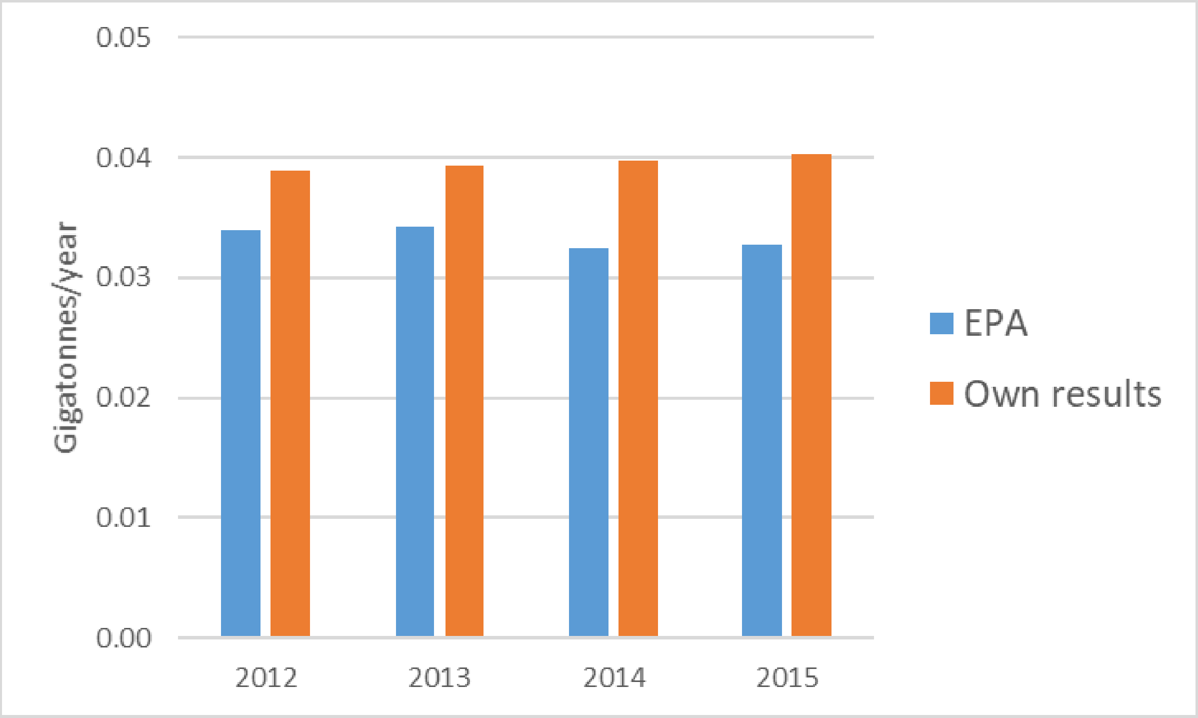

Notes: We classified demolition waste of wood products from U.S. EPA, 2015, 2016, 2018 as End-of-Life outflows for solidwood.

Figure S1.25: Own results for End-of-Life outflows of aluminum in the USA, 1900-2009

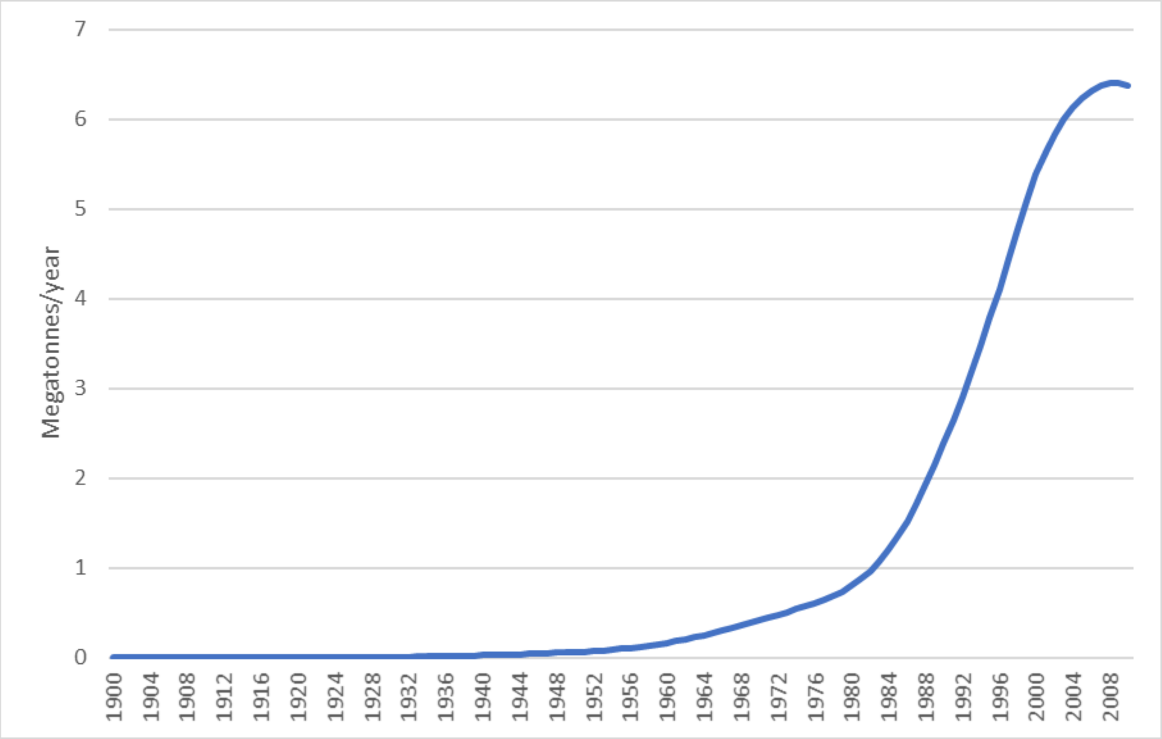

Notes: Estimates are from own calculations.

Figure S1.26: Comparison of estimates of End-of-Life outflows of concrete in the USA for 2012-2015

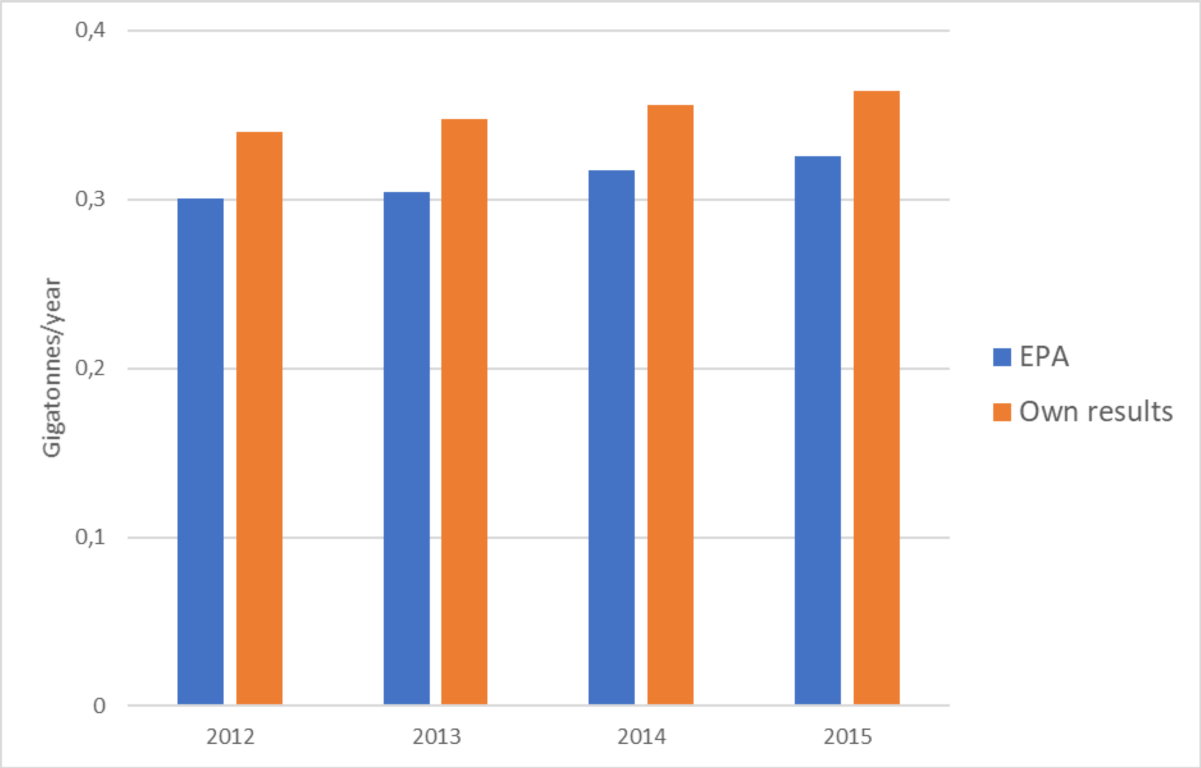

Notes: We classified demolition waste of portland cement concrete from (U.S. EPA, 2015, 2016, 2018) as End-of-Life outflows for concrete.

Figure S1.27: Comparison of estimates of End-of-Life outflows of bricks and stones in the USA for 2012-2015

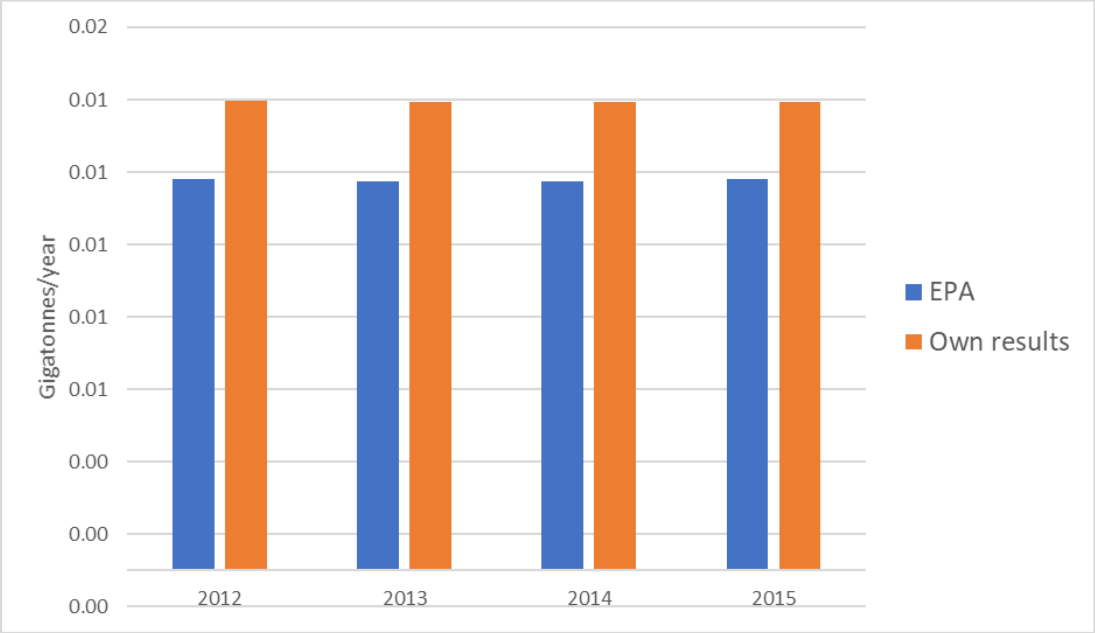

Notes: We classified demolition waste of brick and clay tile from (U.S. EPA, 2015, 2016, 2018) as End-of-Life outflows for bricks and tiles. End-of-Life outflows for stones are included in own results while EPA does not include End-of-Life outflows for stones.

Figure S1.28: Comparison of estimates of End-of-Life outflows of glass in the USA for 1960-2015

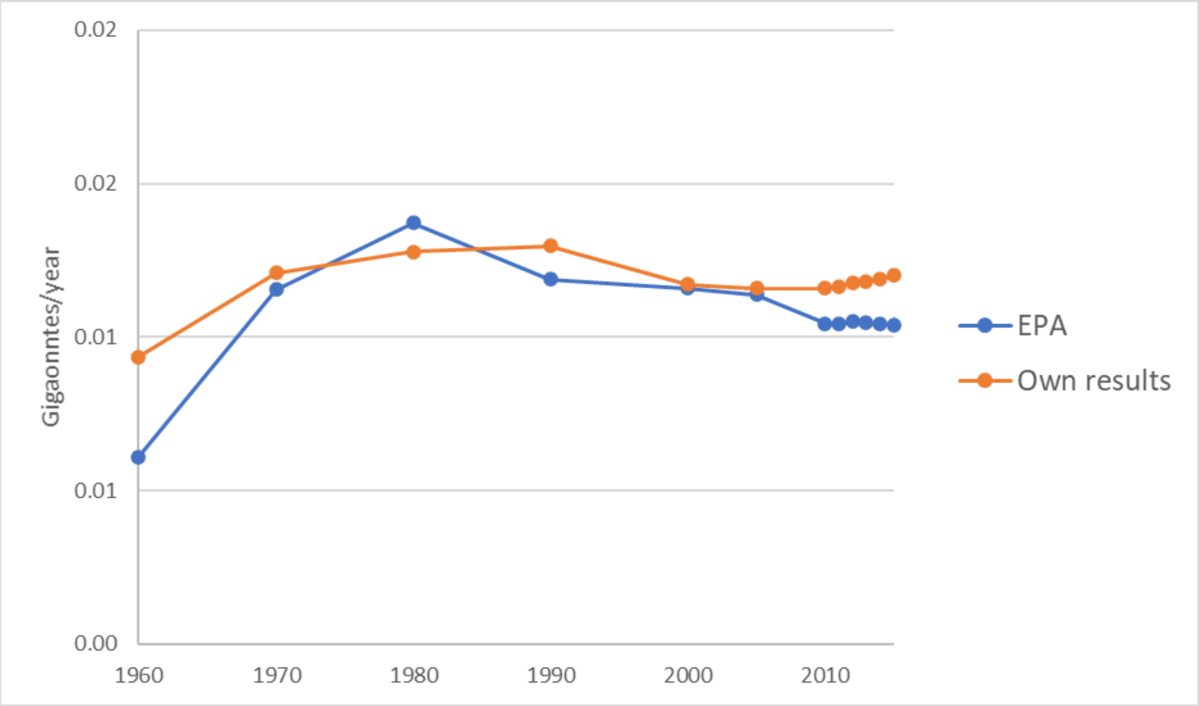

Notes: We classified municipal solid waste of glass from U.S. EPA, 2020 as End-of-Life outflows. Own results include both End-of-Life outflows for container and flat glass.

Figure S1.29: Comparison of estimates of End-of-Life outflows of plastics in the USA for 1960-2015

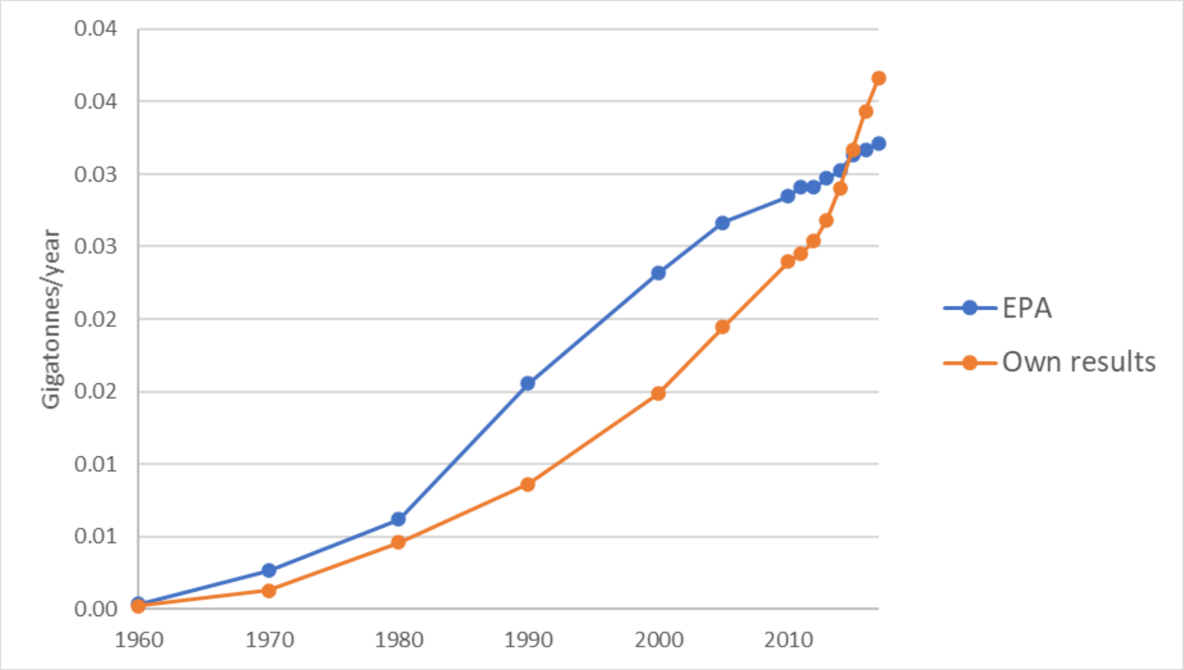

Notes: We classified municipal solid waste of plastics from U.S. EPA, 2020 as End-of-Life outflows.

4.2.3 Final Waste

Figure S1.30: Comparison of estimates of final waste flows of biomass in the USA for 1930-2005

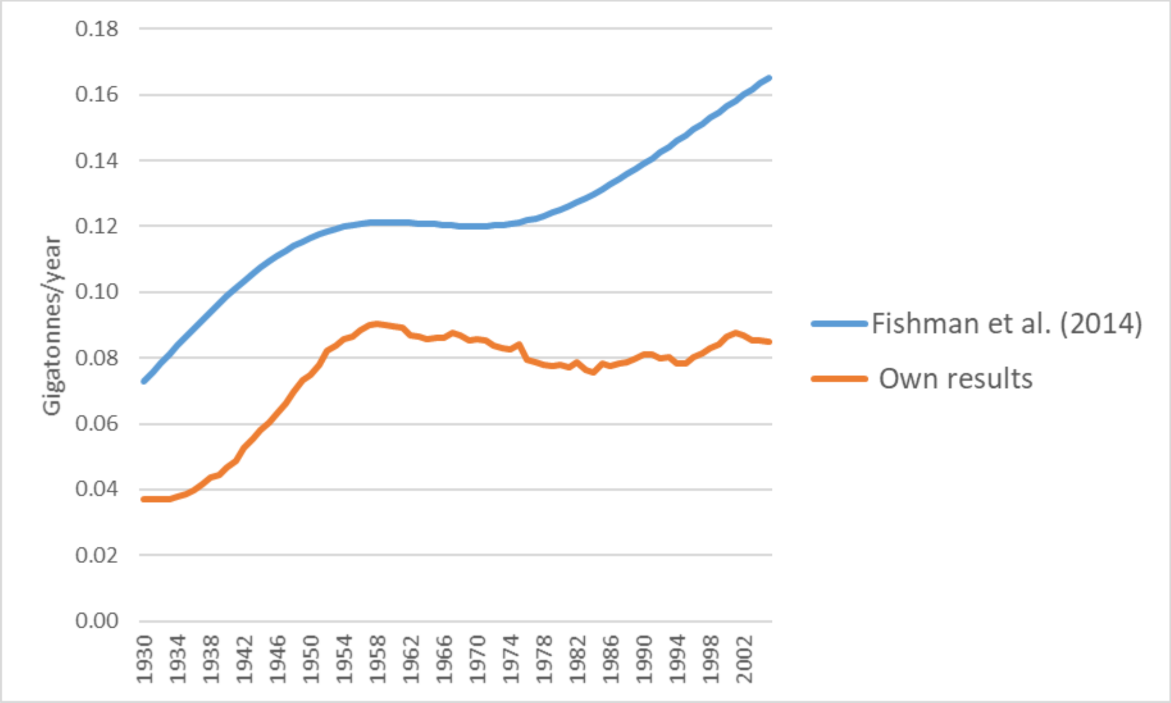

Notes: Own results include final waste for solidwood and paper and paperboard. Final waste estimates from Fishman et al., 2014 include timber.

Figure S1.31: Comparison of estimates of final waste flows of iron/steel in the USA for 1930-2005

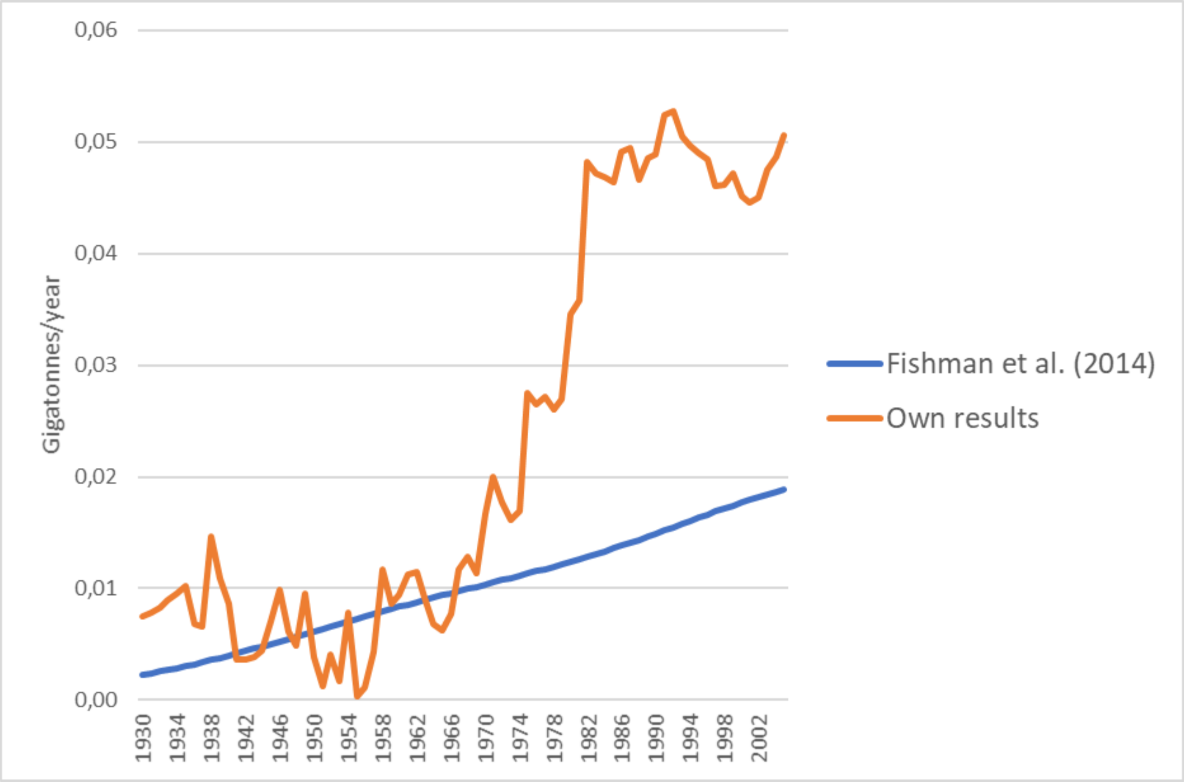

Notes: Own results include final waste for iron/steel. Final waste estimates from Fishman et al., 2014 include iron.

Figure S1.32: Comparison of estimates of final waste flows of non-ferrous metals in the USA for 1930-2005

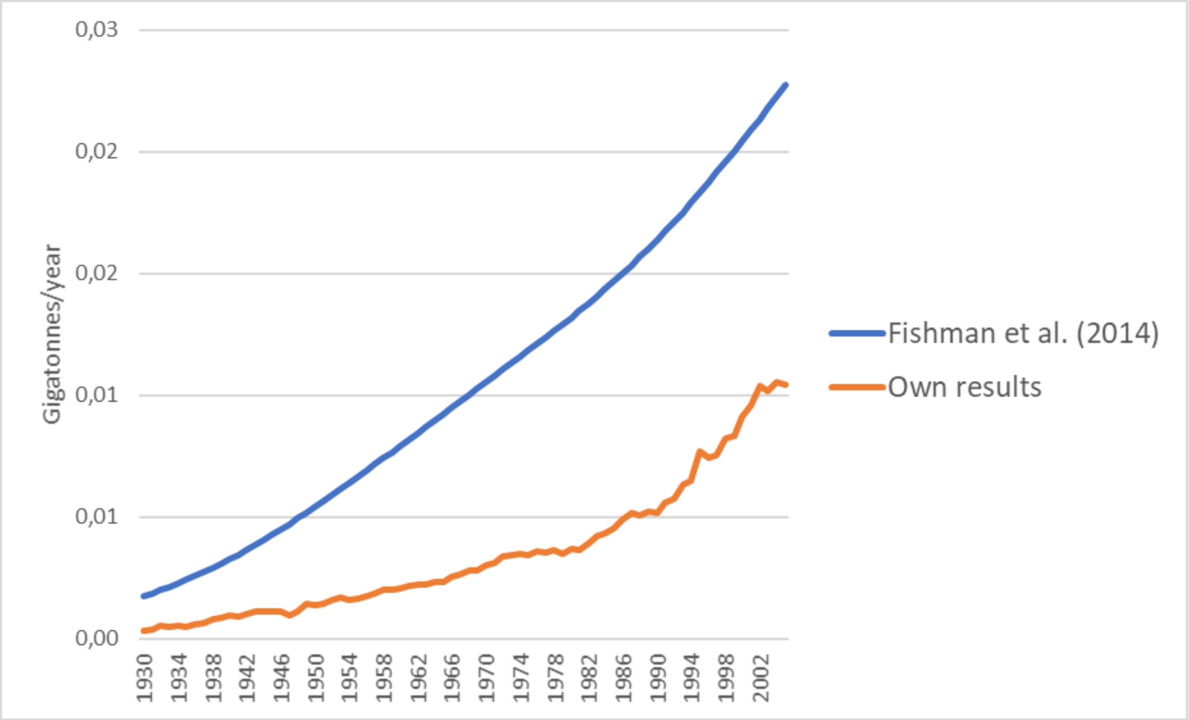

Notes: Own results include final waste for aluminum, copper and other metals. Final waste estimates from Fishman et al., 2014 include all metals other than iron.

Figure S1.33: Comparison of estimates of final waste flows of non-metallic minerals in the USA for 1930-2005

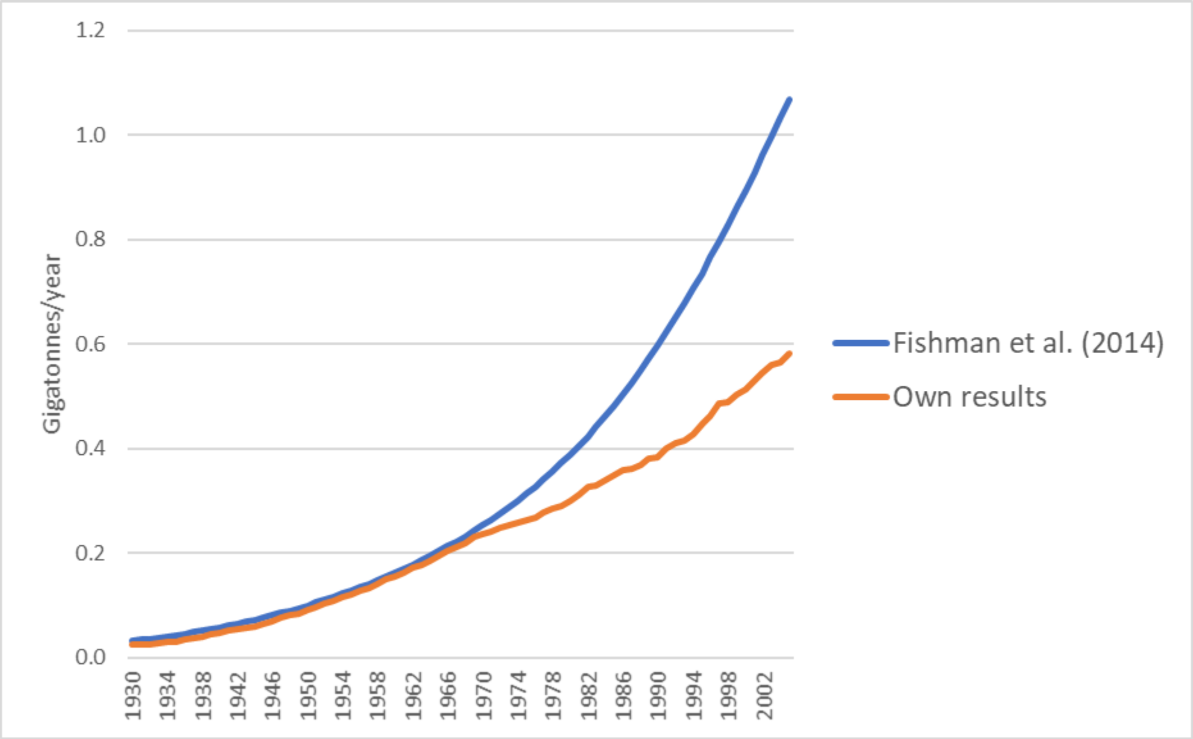

Notes: Own results include final waste for concrete, asphalt, aggregates in sub-base and base-course layers, bricks and stones, container glass and flat glass. Final waste estimates from Fishman et al., 2014 include all non-metallic minerals.

4.2.4 Recycling

Figure S1.34: Comparison of estimates of paper and paperboard recycling rates in the USA for 1960-2005

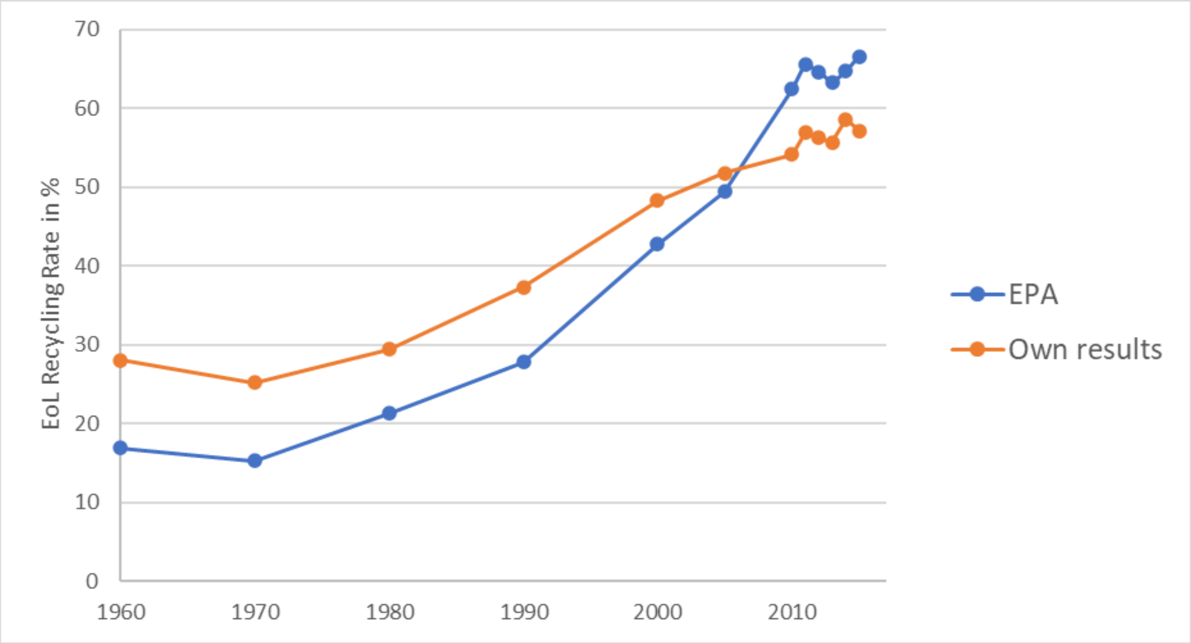

Notes: Recycling rates for U.S. EPA, 2020 are calculated as total paper and paperboard recycled flows divided by municipal solid waste. Own results are calculated as total paper and paperboard recycling flows divided by total End-of-Life outflows.

Figure S1.35: Comparison of estimates of glass recycling flows in the USA for 1960-2005

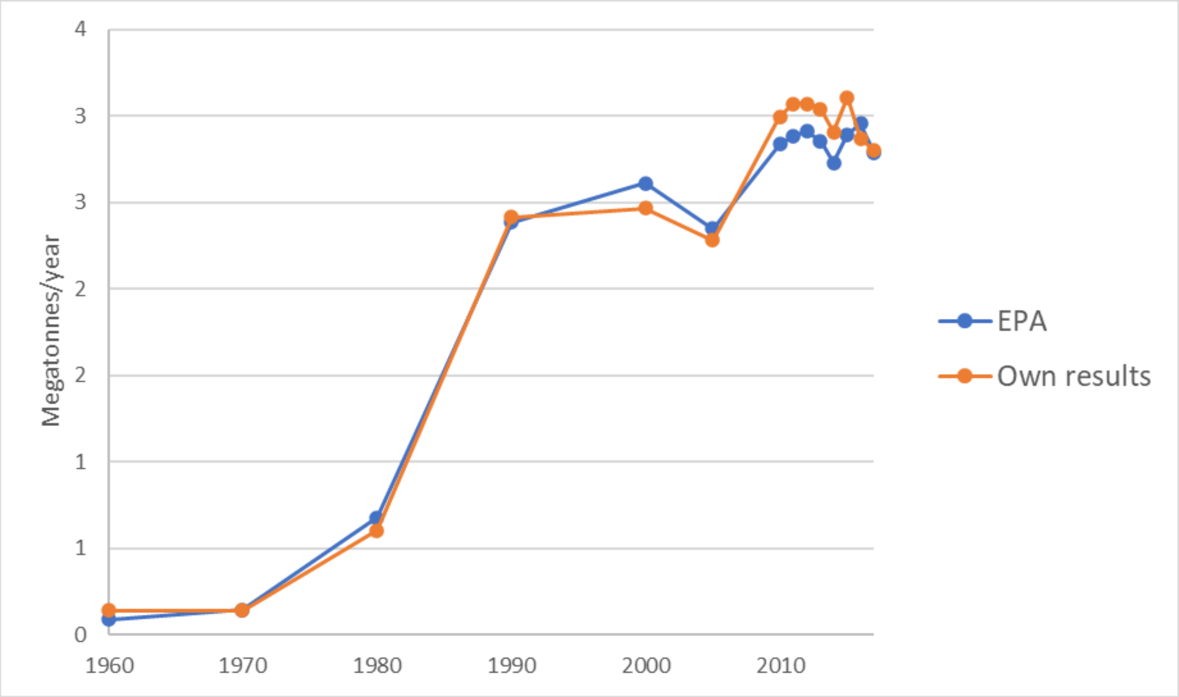

Notes: Values from U.S. EPA, 2020 are recycled municipal solid waste of glass. Own results include container and flat glass.

Figure S1.36: Comparison of estimates of plastics recycling flows in the USA for 1980-2015

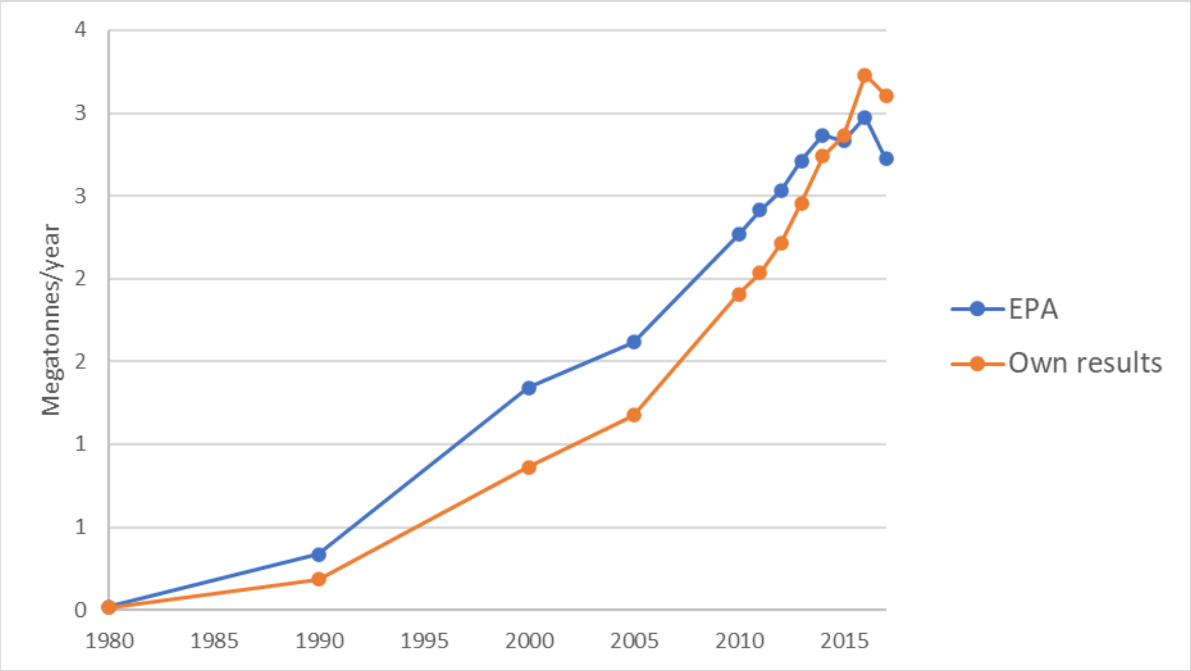

Notes: Values from U.S. EPA, 2020 are recycled municipal solid waste of plastics.

Table S1.10: Comparison of End-of-Life recycling rates for iron/steel, aluminum and copper in the USA for various years

Material	Author	Year	Estimate	Own results
Iron/Steel	Fenton, 2004	1998	46.6 %	43.2 %
Iron/Steel	Wang et al., 2007	2000	61.3 %	43.7 %
Aluminum	Plunkert, 2006	2000	34.3 %	25.4 %
Aluminum	Chen, 2013	1992/2009	49/24 %	54.9/19.6 %
Copper	Graedel et al., 2004	1994	33.8 %	26.6 %
Copper	Goonan, 2009	2004	11.5 %	9.4 %

Notes: Values for Fenton (2004: 3), Plunkert (2006: W6) and Goonan (2009: X3) were calculated as old scrap generated divided by old scrap consumed. The values of Graedel et al. (2004: Supporting Information p. 21) was calculated as old scrap/(old scrap + landfilled waste and dissipated plus + trade of old scrap). For Wang et al. (2007: 5123) we assume that industrial scrap is completely recycled and calculate the EoL recycling rate as (purchased scrap - industrial scrap)/(EoL discards). Values for Chen (2013: 933-934) depicts their reported domestic EoL recycling rate (i.e. excluding exported scrap).

4.3 Additional Figures, Tables and Results

Figure S1.37: Comparison of sand and gravel consumption in the USA 1870-2005 reported by Gierlinger and Krausmann 2012 and replicated figures

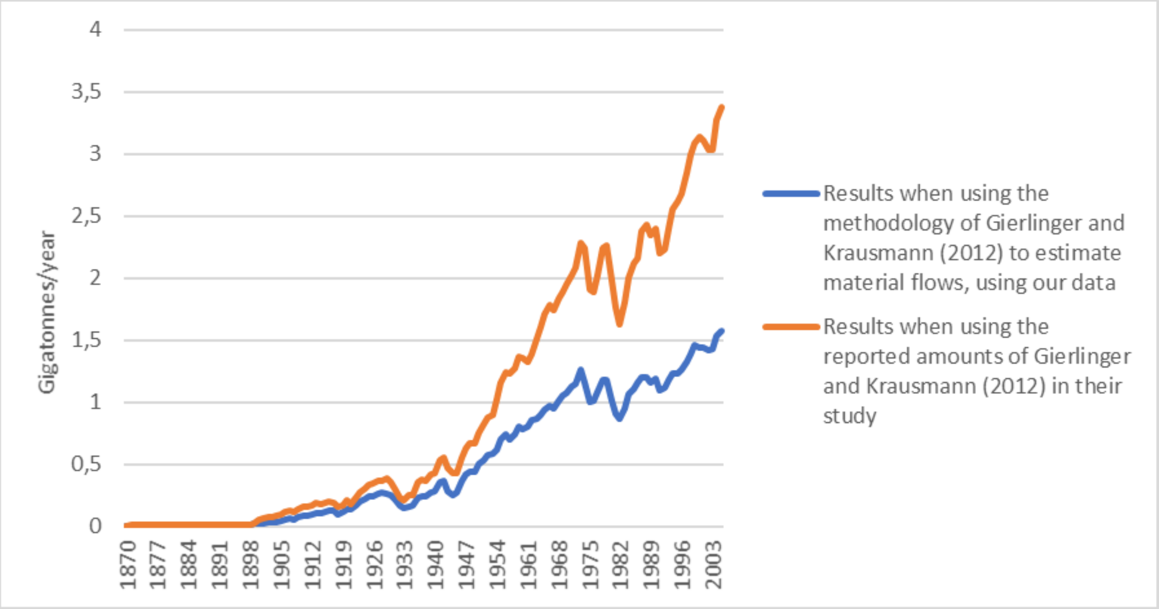

Notes: The figures represent the replication of the results of Gierlinger and Krausmann 2012, using their described methodology. To obtain sand and gravel for concrete, cement consumption was thus multiplied by the factor 6.1. To obtain sand and gravel used for asphalt, bitumen consumption was multiplied by the factor 20. The asphalt estimate was further increased by a factor of 1.5 to account for sand and gravel as filling material. Methods are described in Gierlinger and Krausmann 2012. The figures of Gierlinger and Krausmann 2012 are the reported amounts of sand and gravel consumption of their study. The figure shows that the reported values for sand and gravel are significantly larger than the figures replicated with our data.

Sources: Data for cement and bitumen consumption is from the Bureau of the Census 1975, Kelly and Matos 2014, USGS, 2019 and the IEA, 2019.

Figure S1.38: Difference of net additions to stock for non-metallic minerals between Fishman et al. 2014 and own results in the USA for 1930-2005

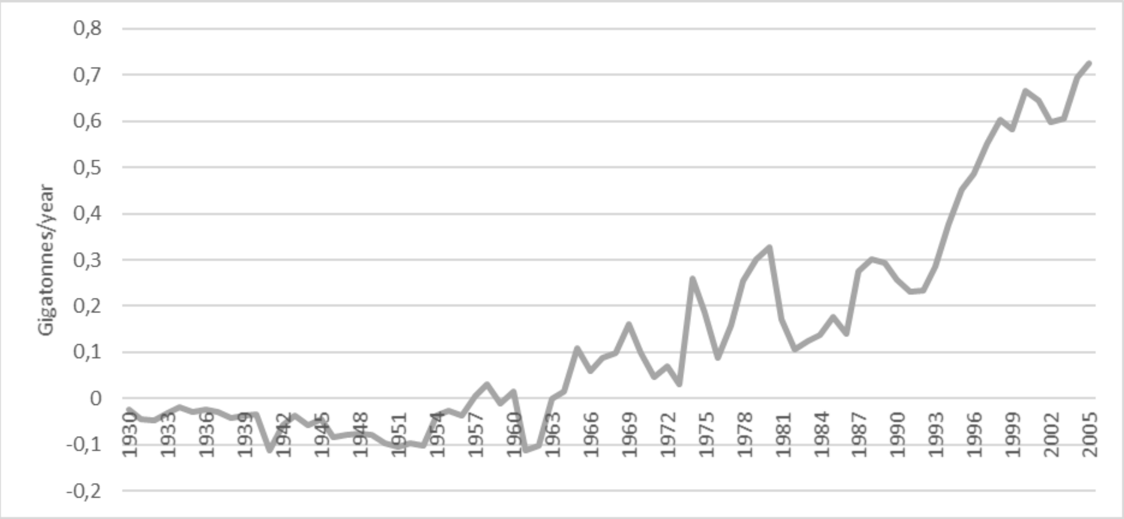

Notes: Differences are calculated as net additions to stock for non-metallic minerals from Fishman et al. 2014 minus net additions to stock for non-metallic minerals from own results. Non-metallic minerals from own results includes concrete, asphalt, bricks and stones, aggregates, flat glass and container glass.

Sources: Fishman et al. 2014, own calculations

Figure S1.39: Sensitivity analysis - material stock level results at different lifetime assumptions. (a) Mean lifetimes reduced by 50%, (b) mean lifetimes (see main paper Table 2) and (c) mean lifetimes extended by 50%.

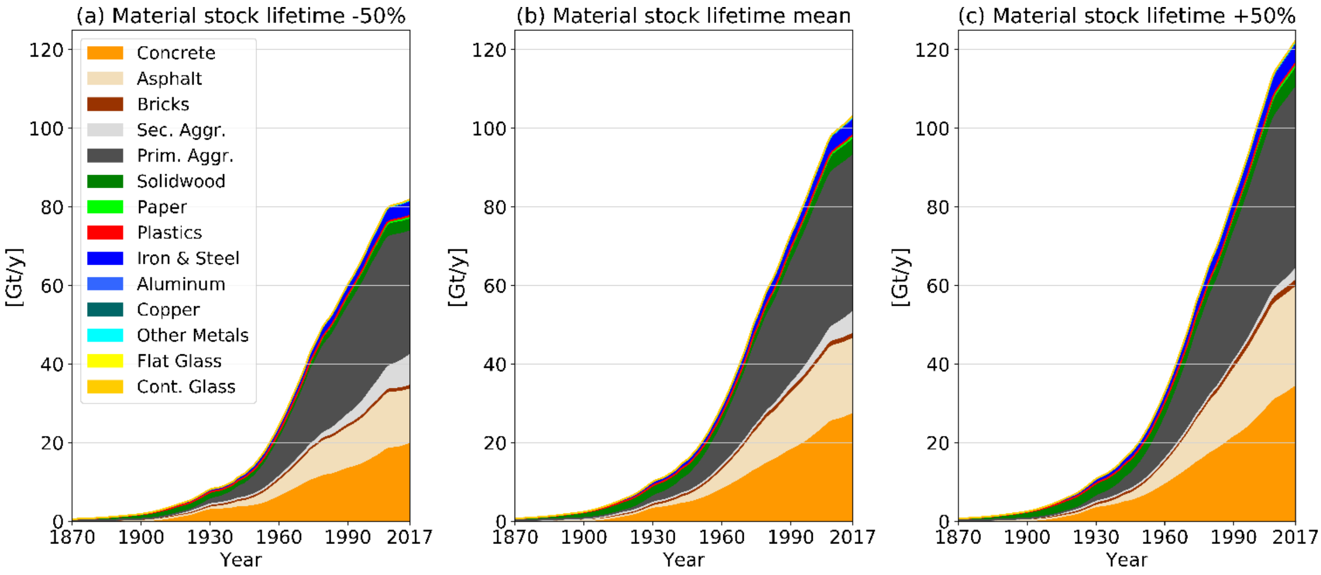

Figure S1.40: Sensitivity analysis – End-of-life outflows from stocks at different lifetime assumptions. (a) Mean lifetimes reduced by 50%, (b) mean lifetimes (see main paper Table 2) and (c) mean lifetimes extended by 50%.

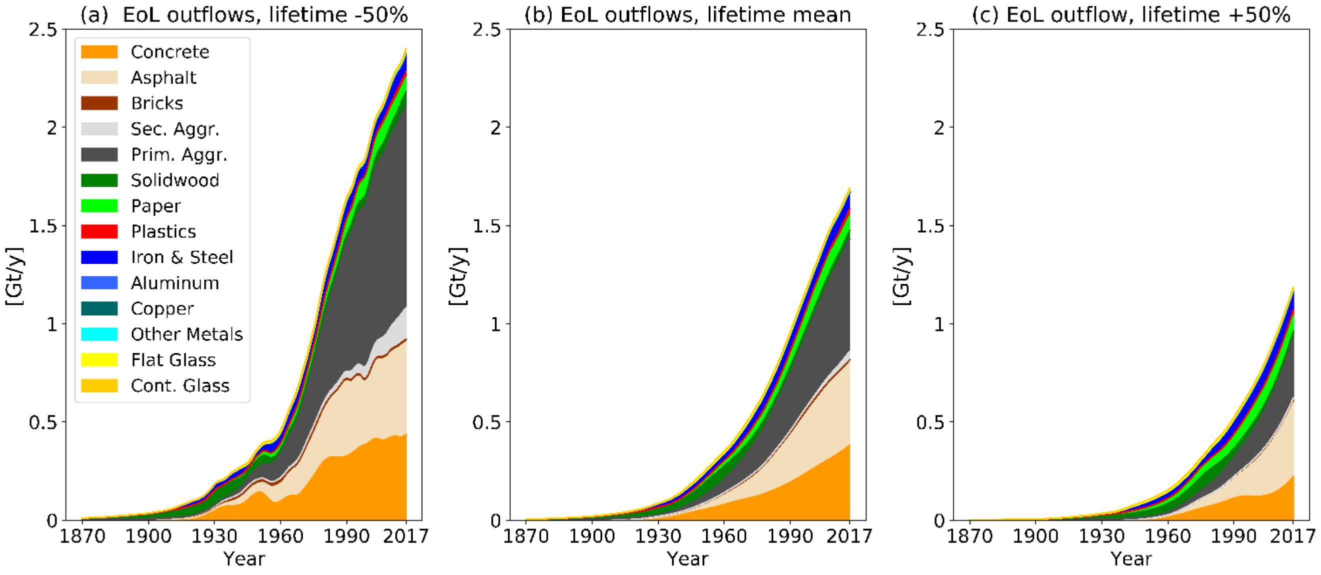

Figure S1.41: Sensitivity analysis - End-of-Life (EoL) outflows from stocks by treatment type for stock-building materials in the USA 1962-2017 when lifetimes are decreased by 50 %

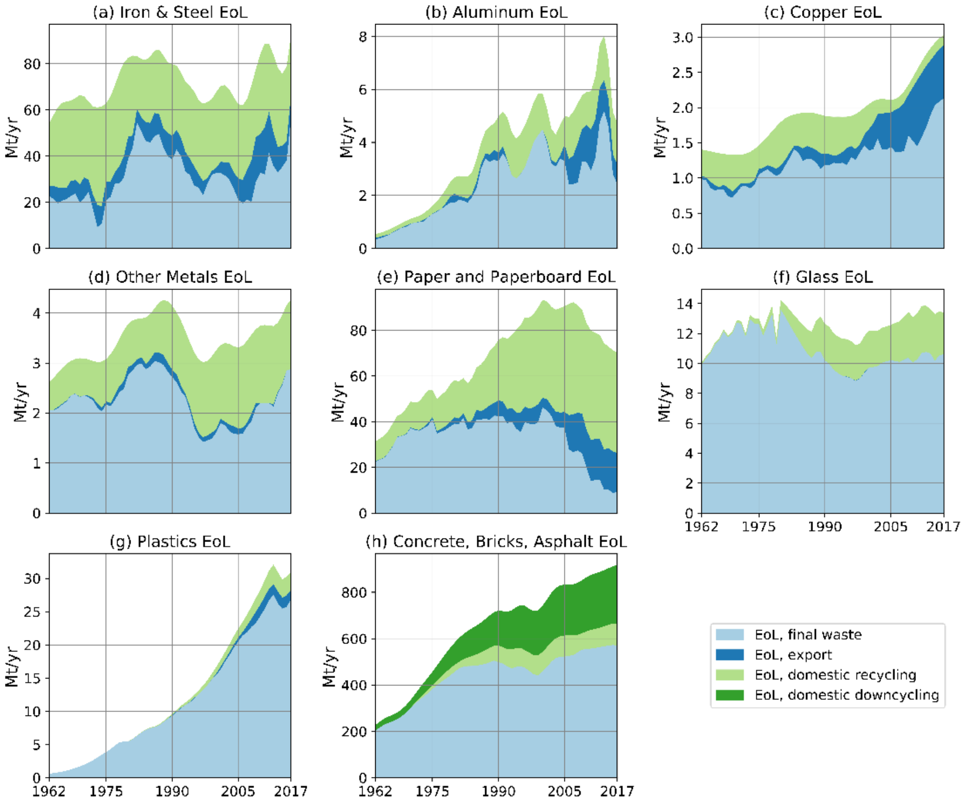

Figure S1.42: Sensitivity analysis - End-of-Life (EoL) outflows from stocks by treatment type for stock-building materials in the USA 1962-2017 when lifetimes are increased by 50 %

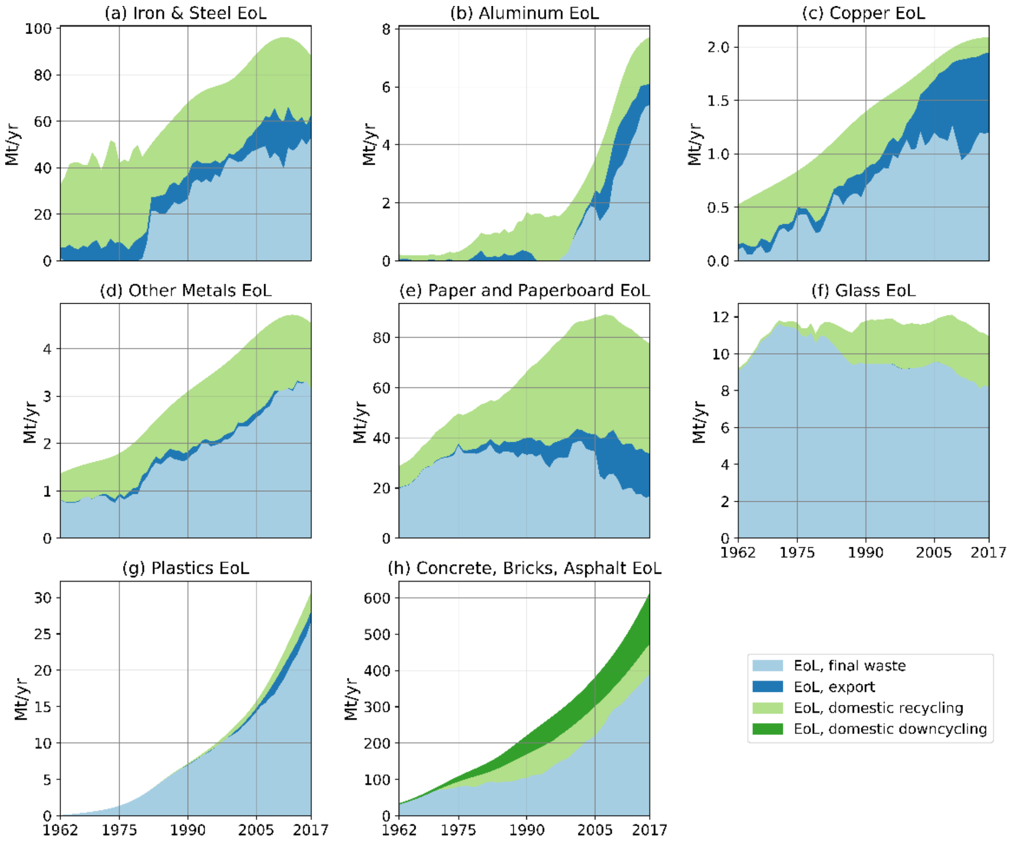

Figure S1.43: Sensitivity analysis - total material stock with conservative baseline multiplier (used in main results) and with modified multiplier based on Wiedenhofer et al., 2021.

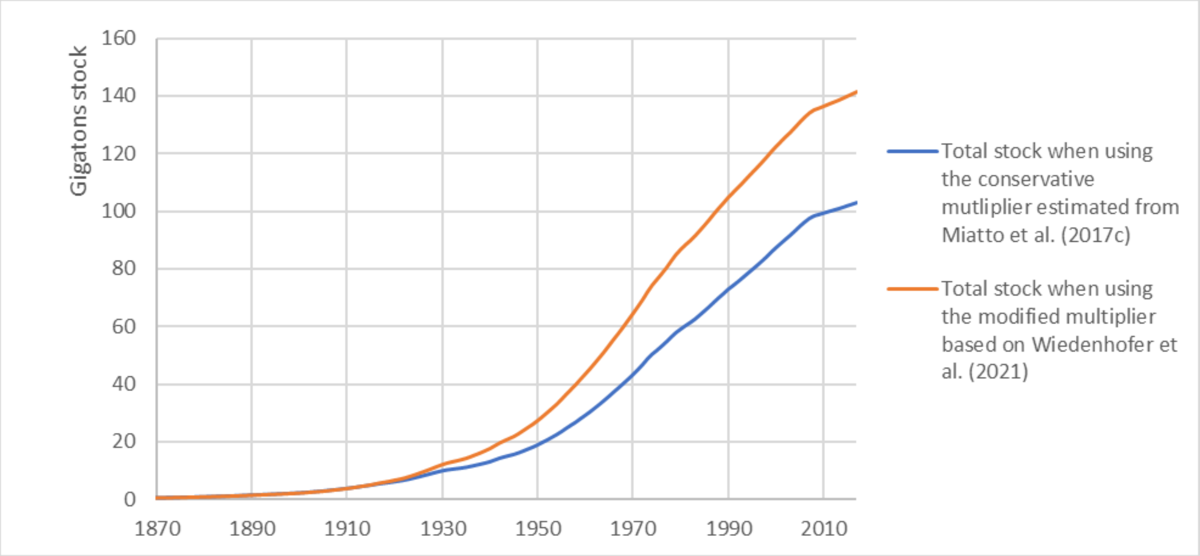

4.3.1 Additional prospective results

Below figures provide additional results on the prospective scenarios. Figure S1.44 shows the total end-of-life outflows (EoL) from stocks for the three prospective scenarios, Figure S1.45 shows the supplementary scenario's ('additional reduction') prospective material stocks, and Figure S1.46 shows prospective EoL outflows for selected materials and required material inputs for scenario 1: 'low growth continuation' in comparison to scenario 3: 'additional reduction'.

For scenario 3: 'additional reduction', an additional reduction in material inputs to stocks, equivalent to the one observed after the 2007 financial crisis (-32% from 2006 to 2009), was assumed which reduced inputs to stock to a total of 1.6 Gt/yr from 2020-2100. As a result, material stocks start to decline from 2017 onwards until they stabilize around 2070 close to 82 Gt (see Figure S1.45). Accordingly, cumulative inputs to stocks to from 2018 to 2100 are 17% lower (297 Gt) compared to the 356 Gt in the 'low growth continuation' scenario. Regarding EoL outflows, scenario 3 leads to an outflow peak in 2036 and afterwards to a decline to below 1.5 Gt/yr (Figure S1.44).

Figure S1.46 shows that scenario 3: 'additional reduction' would improve the match of EoL outflows from stocks to material inputs to stock, compared to the 'low growth continuation' scenario.

Figure S1.44: EoL outflows for scenarios, ‘low growth continuation’ (a), ‘high growth return’ (b) and ‘additional reduction (c).

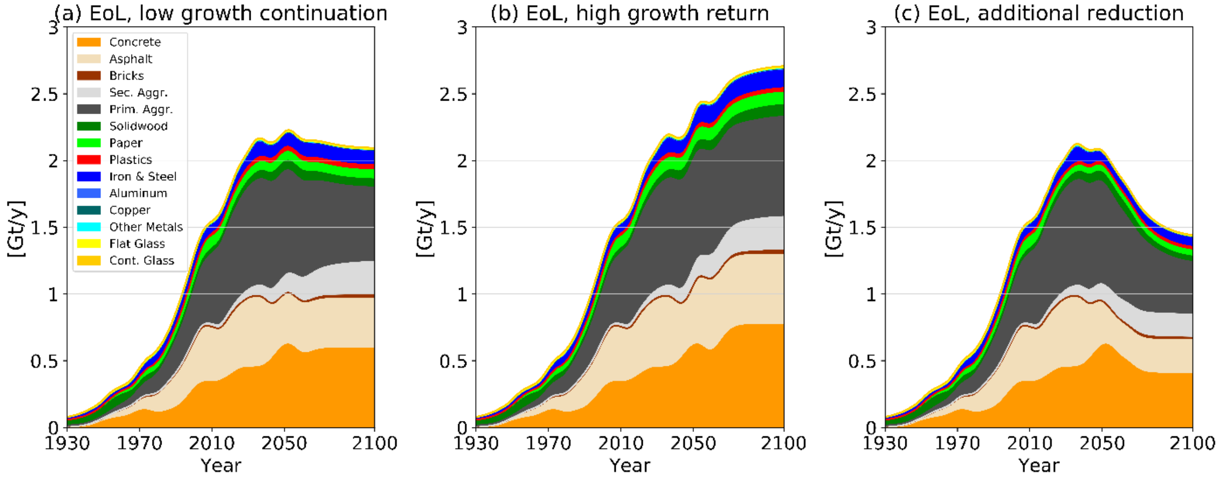

Figure S1.45: Prospective development of material stocks to 2100 in scenario 3: ‘additional reduction’.

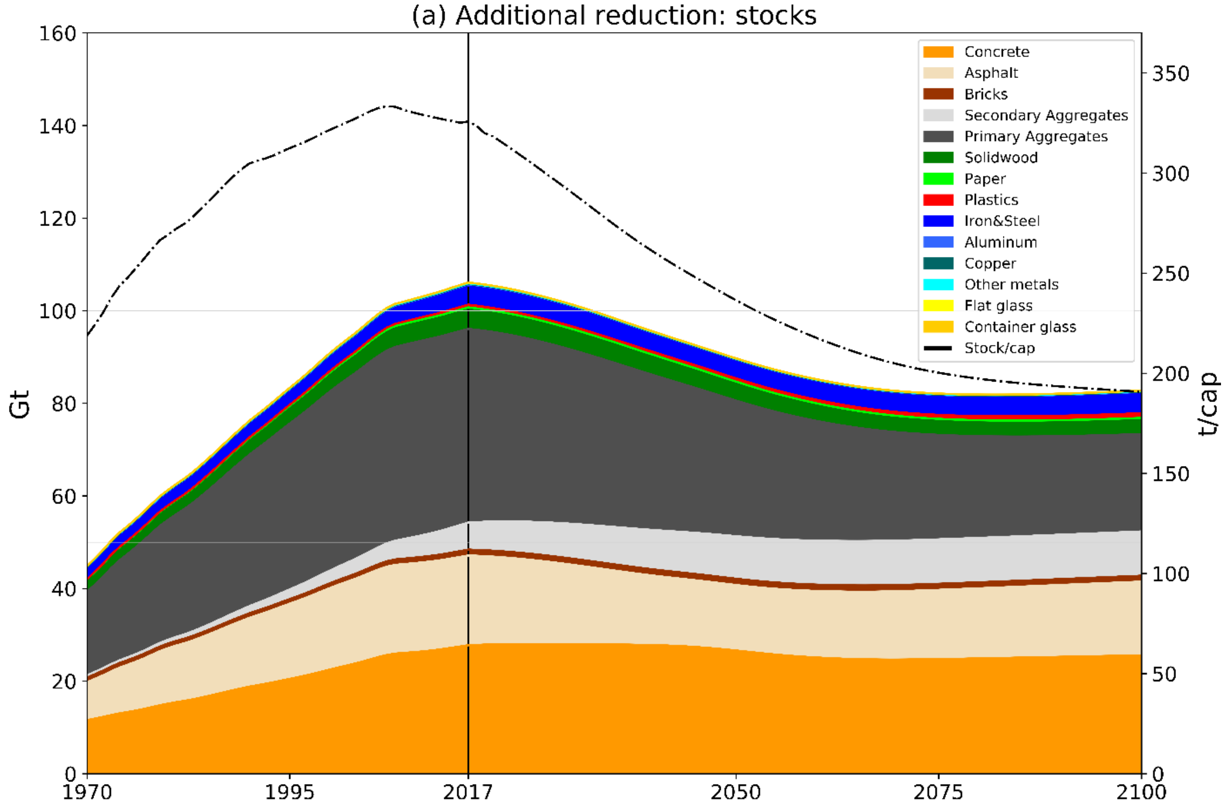

Notes: Prospective development of material stocks to 2100 assuming that a similar long term drop of inputs to stocks (-32%) occurs in as has been observed after the global financial crisis in 2007/8. In this ‘additional reduction’ scenario, we keep this low level of inputs to stocks constant until 2100. As for the other prospective scenarios, all other parameters were kept constant on 2017 levels (see main manuscript section 2.4).

Figure S1.46: Historical inputs to stock and end-of-life (EoL) outflows from 1970-2017 and modelled inputs for 2018-2100 under the scenario conditions for scenario 1: ‘low growth continuation’ and scenario 3: ‘additional reduction’

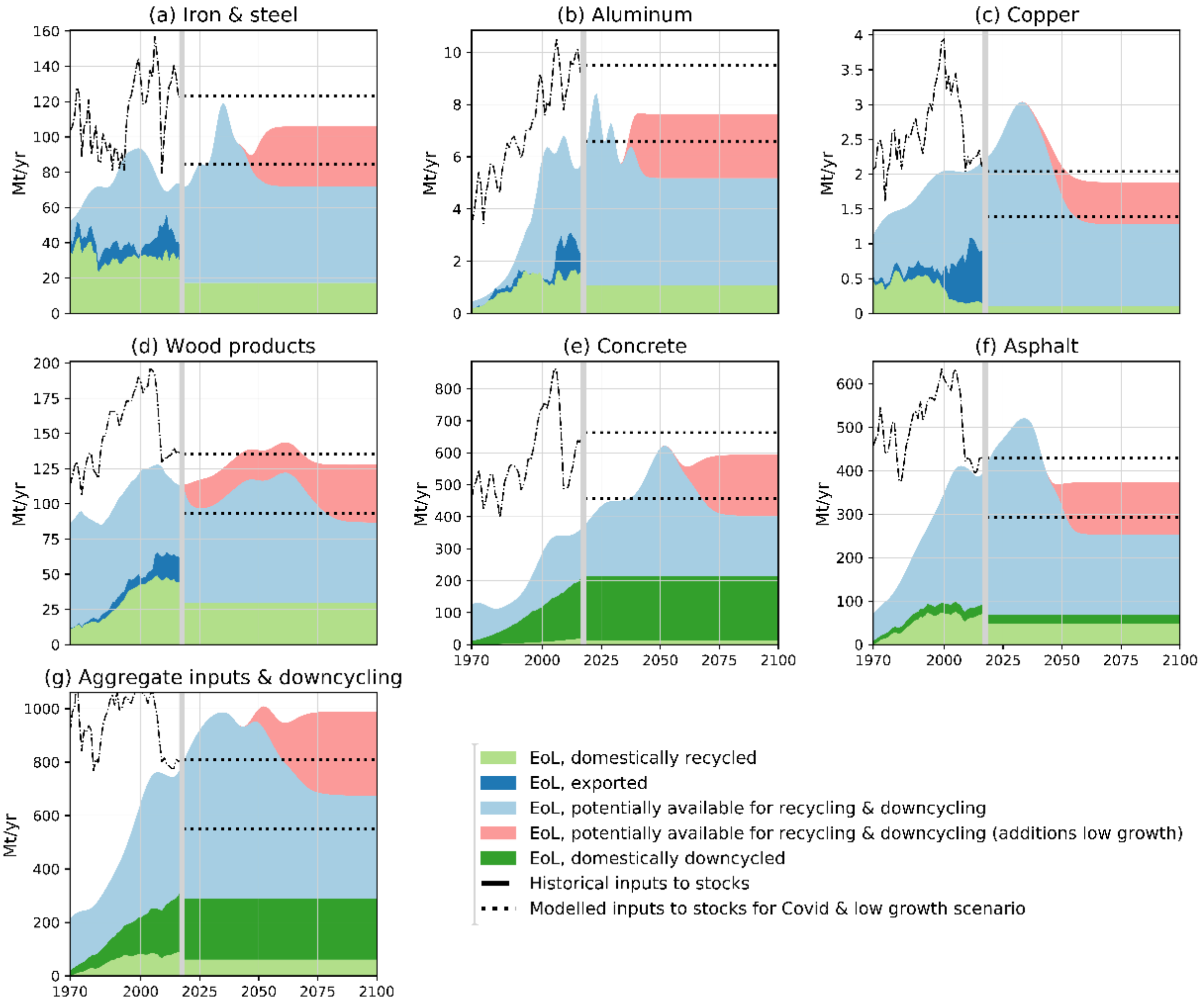

Notes: The figure shows which part of EoL outflows was recycled (light green), exported (dark blue), downcycled (dark green), and remained as outflows of final waste (light blue) for 1970-2017. For 2018-2100 the assessment is prospective. The pink area reflects the additional EoL outflows in scenario 1: ‘low growth continuation’ compared to scenario 3: ‘additional reduction’. For description of the scenarios please see main manuscript section 2.4, SI 4.3.1 and the caption of Figure S1.45.

Table S1.11: Commodity codes used to estimate net trade in addition to Streeck et al. (2020).

**Material**	**Commodity Code**
Tin	SITC1: 28409 (waste & scrap)
Nickel	SITC1: 28403 (waste & scrap)
Glass	SITC1: 664,665; HS92: 7001 (glass waste)
Sand & gravel	SITC1: 273
Plastic	SITC3: 579 (waste & scrap)

References

ACC. (2019). *Store: Economic: The Resin Review 2019 (electronic version)*. https://store.americanchemistry.com/ResinReview2019PDF

Aluminum Leader. (2018). *Aluminum Production*. https://www.aluminiumleader.com/production/aluminum_production/

American Foundry Society. (2011-2020). *Modern Casting*. https://www.moderncasting.com/issues

Ayres, R. U., Ayres, L. W., & Råde, I. (2002). The Life Cycle of Copper, itsCo-Products and By-Products.

Bento, A., Roth, K., & Zuo, Y. (2016). *Vehicle Lifetime Trends and Scrappage Behavior in the U.S. Used Car Market*. http://faculty.sites.uci.edu/kevinroth/files/2011/03/Scrappage_18Jan2016.pdf

Bolt, J., Inklaar, R., Jong, H. de, & van Zanden, J. L. (2018). *Rebasing Maddison: new income comparisons and the shape of long-run economic development"*. www.ggdc.net/maddison

Brown, H. (1954). *The Challenge of Man's Future;AnInquiry Concerning the Condition of Man during the Years That Lie Ahead: p. 290*. Viking.

Bureau of the Census. (1949). *Historical Statistics of the United States 1789-1945*. https://www2.census.gov/library/publications/1949/compendia/hist_stats_1789-1945/hist_stats_1789-1945.pdf?#

Bureau of the Census. (1975). *Bicentennial Edition: Historical Statistics of the United States, Colonial Times to 1970*. https://www.census.gov/library/publications/1975/compendia/hist_stats_colonial-1970.html

Butler, J. H., & Hooper, P. (2011). Chapter 11 - Glass Waste. In T. M. Letcher & D. A. Vallero (Eds.), *Waste: A handbook for management / edited by Trevor Letcher, Daniel Vallero* (pp. 151–165). Academic. https://doi.org/10.1016/B978-0-12-381475-3.10011-7

Cao, Z., Shen, L., Løvik, A. N., Müller, D. B., & Liu, G. (2017). Elaborating the History of Our Cementing Societies: An in-Use Stock Perspective. *Environmental Science & Technology*, *51*(19), 11468–11475. https://doi.org/10.1021/acs.est.7b03077

Cembureau. (1998). *World Statistical Review: World Cement Market in Figures 1913/1995*. Cembureau, Cement Statistical and Technical Association.

Cembureau. (2005). *World Statistical Review N° 19 - 26 / 1996 - 2003: Cement Production, Trade, Consumption Data*. Brussels.

Cembureau. (2017). *World Statistical Review 2004-2014: Cement Production, Trade, Consumption Data*. Brussels.

Chen, W.-Q. (2013). Recycling Rates of Aluminum in the United States. *Journal of Industrial Ecology*, *17*(6), 926–938. https://doi.org/10.1111/jiec.12070

Chen, W.-Q., & Graedel, T. E. (2012). Dynamic analysis of aluminum stocks and flows in the United States: 1900–2009. *Ecological Economics*, *81*, 92–102. https://doi.org/10.1016/j.ecolecon.2012.06.008

Cochran, K. M., & Townsend, T. G. (2010). Estimating construction and demolition debris generation using a materials flow analysis approach. *Waste Management (New York, N.Y.)*, *30*(11), 2247–2254. https://doi.org/10.1016/j.wasman.2010.04.008

Cullen, J. M., Allwood, J. M., & Bambach, M. D. (2012). Mapping the global flow of steel: From steelmaking to end-use goods. *Environmental Science & Technology*, *46*(24), 13048–13055. https://doi.org/10.1021/es302433p

FAO. (2019). *FAOSTAT-Forestry database*. http://www.fao.org/forestry/statistics/84922/en/

Fenton, M. D. (2004). *Iron and Steel Recycling in the United States in 1998: USGS Open-File Report*. https://pubs.er.usgs.gov/publication/ofr01224

FHA. (2016). *User Guidelines for Waste and Byproduct Materials in Pavement Construction: Reclaimed Asphalt Pavement*. https://www.fhwa.dot.gov/publications/research/infrastructure/structures/97148/rap132.cfm

Fishman, T., Schandl, H., Tanikawa, H., Walker, P., & Krausmann, F. (2014). Accounting for the Material Stock of Nations: Accounting for the Material Stock of Nations. *Journal of Industrial Ecology*, *18*(3), 407–420. https://doi.org/10.1111/jiec.12114

Gerst, M. D., & Graedel, T. E. (2008). In-Use Stocks of Metals: Status and Implications. *Environmental Science & Technology*, *42*(19), 7038–7045. https://doi.org/10.1021/es800420p

Geyer, R., Jambeck, J. R., & Law, K. L. (2017). Production, use, and fate of all plastics ever made. *Science Advances*, *3*(7), e1700782. https://doi.org/10.1126/sciadv.1700782

Gierlinger, S., & Krausmann, F. (2012). The Physical Economy of the United States of America. *Journal of Industrial Ecology*, *16*(3), 365–377. https://doi.org/10.1111/j.1530-9290.2011.00404.x

Glöser, S., Soulier, M., & Tercero Espinoza, L. A. (2013). Dynamic Analysis of Global Copper Flows. Global Stocks, Postconsumer Material Flows, Recycling Indicators, and Uncertainty Evaluation. *Environmental Science & Technology*, *47*(12), 6564–6572. https://doi.org/10.1021/es400069b

Goonan, T. (2009). *Cooper Recycling in the United States in 2004*. https://pubs.usgs.gov/circ/circ1196x/pdf/circ1196X.pdf

Gordon, R. B., Bertram, M., & Graedel, T. E. (2006). Metal stocks and sustainability. *Proceedings of the National Academy of Sciences*, *103*(5), 1209–1214. https://doi.org/10.1073/pnas.0509498103

Graedel, T. E., van Beers, D., Bertram, M., Fuse, K., Gordon, R. B., Gritsinin, A., Kapur, A., Klee, R. J., Lifset, R. J., Memon, L., Rechberger, H., Spatari, S., & Vexler, D. (2004). Multilevel cycle of anthropogenic copper. *Environmental Science & Technology*, *38*(4), 1242–1252. https://doi.org/10.1021/es030433c

Graedel, T.E., Bertram, M., Fuse, K., Gordon, R.B., Lifset, R., Rechberger, H., & Spatari, S. (2002). The contemporary European copper cycle: The characterization of technological copper cycles. *Ecological Economics*, *42*(1-2), 9–26. https://doi.org/10.1016/S0921-8009(02)00101-5

IEA. (2019). *World Energy Balances*. https://www.iea.org/statistics/balances/

Ingalls, W. R. (1935). *The Economics of Old Metals Especially Copper, Lead, Zinc, and Tin: (pp 75-97)*. Mining and Metallurgical Society of America.

Jolly, J. H. (1993). Materials flow of zinc in the United States 1850–1990. *Resources, Conservation and Recycling*, *9*(1-2), 1–30. https://doi.org/10.1016/0921-3449(93)90031-A

Kapur, A., Keoleian, G., Kendall, A., & Kesler, S. E. (2008). Dynamic Modeling of In-Use Cement Stocks in the United States. *Journal of Industrial Ecology*, *12*(4), 539–556. https://doi.org/10.1111/j.1530-9290.2008.00055.x

Kapur, A., van Oss, H. G., Keoleian, G., Kesler, S. E., & Kendall, A. (2009). The contemporary cement cycle of the United States. *Journal of Material Cycles and Waste Management*, *11*(2), 155–165. https://doi.org/10.1007/s10163-008-0229-x

Kelly, T. D. (1998). *Crushed Cement Concrete Substitution for Construction Aggregates—A Materials Flow Analysis: USGS Circular, 1177*.

Kelly, T. D., & Matos, G.R. (2014). *Historical statistics for mineral and material commodities in the United States (2016 version): U.S. Geological Survey Data Series 140*. https://minerals.usgs.gov/minerals/pubs/historical-statistics/

Krausmann, F., Weisz, H., Eisenmenger, N., Schütz, H., Haas, W., & Schaffartzik, A. (2018). *Economy-wide Material Flow Accounting Introduction and Guide Version 1.0*. https://www.wiso.boku.ac.at/fileadmin/data/H03000/H73000/H73700/Publikationen/Working_Papers/working-paper-151-web.pdf

Krausmann, F., Wiedenhofer, D., Lauk, C., Haas, W., Tanikawa, H., Fishman, T., Miatto, A., Schandl, H., & Haberl, H. (2017). Global socioeconomic material stocks rise 23-fold over the 20th century and require half of annual resource use. *Proceedings of the National Academy of Sciences*, *114*(8), 1880–1885. https://doi.org/10.1073/pnas.1613773114

Liu, G., Bangs, C. E., & Müller, D. B. (2011). Unearthing potentials for decarbonizing the U.S. Aluminum cycle. *Environmental Science & Technology*, *45*(22), 9515–9522. https://doi.org/10.1021/es202211w

Liu, G., & Müller, D. B. (2013). Mapping the global journey of anthropogenic aluminum: A trade-linked multilevel material flow analysis. *Environmental Science & Technology*, *47*(20), 11873–11881. https://doi.org/10.1021/es4024404

McMahon, A. D. (1965). *Copper: A Materials Survey.*

McMillan, C. A., Moore, M. R., Keoleian, G. A., & Bulkley, J. W. (2010). Quantifying U.S. aluminum in-use stocks and their relationship with economic output. *Ecological Economics*, *69*(12), 2606–2613. https://doi.org/10.1016/j.ecolecon.2010.08.005

Merrill, C. W. (Ed.) (1949). *The accumulation and conservation of metals in- use*.

Merrill, C. W. (1959). The significance of the mineral industries in the economy. In E. H. Robie (Ed.), *Economics of the Mineral Industries; A Series of Articles by Specialists* (1st ed., p. 787). American Institute of Mining, Metallurgical, and Petroleum Engineers.

Miatto, A., Schandl, H., Fishman, T., & Tanikawa, H. (2017). Global Patterns and Trends for Non-Metallic Minerals used for Construction: Global Non-Metallic Minerals Account. *Journal of Industrial Ecology*, *21*(4), 924–937. https://doi.org/10.1111/jiec.12471

Miatto, A., Schandl, H., & Tanikawa, H. (2017). How important are realistic building lifespan assumptions for material stock and demolition waste accounts? *Resources, Conservation and Recycling*, *122*, 143–154. https://doi.org/10.1016/j.resconrec.2017.01.015

Miatto, A., Schandl, H., Wiedenhofer, D., Krausmann, F., & Tanikawa, H. (2017). Modeling material flows and stocks of the road network in the United States 1905–2015. *Resources, Conservation and Recycling*, *127*, 168–178. https://doi.org/10.1016/j.resconrec.2017.08.024

Müller, D. B., Wang, T., Duval, B., & Graedel, T. E. (2006). Exploring the engine of anthropogenic iron cycles. *Proceedings of the National Academy of Sciences*, *103*(44), 16111–16116. https://doi.org/10.1073/pnas.0603375103

Müller, D. B., Wang, T., & Duval, B. (2011). Patterns of Iron Use in Societal Evolution. *Environmental Science & Technology*, *45*(1), 182–188. https://doi.org/10.1021/es102273t

NAPA. (2019a). *History of Asphalt*. http://www.asphaltpavement.org/index.php?option=com_content&task=view&id=21&Itemid=41

NAPA. (2019b). *Market Facts*. http://www.asphaltpavement.org/index.php?option=com_content&view=article&id=891

NAPA. (2019c). *Recycling*. http://www.asphaltpavement.org/recycling

Nathan Associates. (2004). *"The National Inventory of Obsolete Copper Scrap: Accumulation and Availability, 1982-2003*. https://www.isri.org/docs/default-source/recycling-analysis-%28reports-studies%29/nathan-report-the-national-inventory-of-obsolete-copper-scrap-2004.pdf

Pauliuk, S., Wang, T., & Müller, D. B. (2013). Steel all over the world: Estimating in-use stocks of iron for 200 countries. *Resources, Conservation and Recycling*, *71*, 22–30. https://doi.org/10.1016/j.resconrec.2012.11.008

Penman, J. (2003). *Good practice guidance for land use, land-use change and forestry*. Published by the Institute for Global Environmental Strategies for the IPCC. http://www.ipcc-nggip.iges.or.jp/public/gpglulucf/gpglulucf%5Fcontents.htm

Plastics Europe. (2019). *European Plastics Industry Market Data*. https://www.plasticseurope.org/en/resources/market-data

Plunkert, P. A. (2006). *Aluminum Recycling in the United States in 2000*. https://pubs.er.usgs.gov/publication/cir1196W

Pyshyev, S., Gunka, V., Grytsenko, Y., & Bratychak, M. (2016). POLYMER MODIFIED BITUMEN: REVIEW. *Chemistry & Chemical Technology*, *10*(4s), 631–636. https://doi.org/10.23939/chcht10.04si.631

Rauch, J. N. (2009). Global mapping of Al, Cu, Fe, and Zn in-use stocks and in-ground resources. *Proceedings of the National Academy of Sciences of the United States of America*, *106*(45), 18920–18925. https://doi.org/10.1073/pnas.0900658106

Ruth, M., & Dell'Anno, P. (1997). An industrial ecology of the US glass industry. *Resources Policy*, *23*(3), 109–124. https://doi.org/10.1016/S0301-4207(97)00020-2

Sandler, K. (2003). Analyzing what´s recyclable in C&D Debris". *Biocycle*, *BioCycle 44*(11), 51–54.

Sousa, L. J. (1981). *The U.S. Copper Industry: Problems, Issues, and Outlook.*

Spatari, S., Bertram, M., Gordon, R. B., Henderson, K., & Graedel, T. E. (2005). Twentieth century copper stocks and flows in North America: A dynamic analysis. *Ecological Economics*, *54*(1), 37–51. https://doi.org/10.1016/j.ecolecon.2004.11.018

Streeck, J., Wiedenhofer, D., Krausmann, F., & Haberl, H. (2020). Stock-flow relations in the socio-economic metabolism of the United Kingdom 1800–2017. *Resources, Conservation and Recycling*, *161*, 104960. https://doi.org/10.1016/j.resconrec.2020.104960

Sullivan, D. E. (2003). *Indicators of Stocks-in -use in the United States for Aluminum, Copper, Gold, Iron, Steel, Lead, and Zinc: Unpublished report.* United States Geological Survey.

Sullivan, D. E. (2005). *Metal Stocks in Use in the United States: USGS Fact Sheet*. https://pubs.usgs.gov/fs/2005/3090/2005-3090.pdf

U.S. EPA. (2015). *Advancing Sustainable Materials Management: Facts and Figures 2013*. https://www.epa.gov/sites/production/files/2015-09/documents/2013_advncng_smm_rpt.pdf

U.S. EPA. (2016). *Advancing Sustainable Materials Management: 2014 Fact Sheet*. https://www.epa.gov/sites/production/files/2016-11/documents/2014_smmfactsheet_508.pdf

U.S. EPA. (2018). *Advancing Sustainable Materials Management: 2015 Fact Sheet*. https://www.epa.gov/sites/production/files/2018-07/documents/2015_smm_msw_factsheet_07242018_fnl_508_002.pdf

U.S. EPA. (2020). *Sustainable Materials Management (SMM) - Materials and Waste Management in the United States Key Facts and Figures*. https://edg.epa.gov/metadata/catalog/search/resource/details.page?uuid=C9310A59-16D2-4002-B36B-2B0A1C637D4E

UNEP (2011). Recycling Rates of Metals – A Status Report.

United Nations Commodity Trade Statistics Database [Computer software]. (2019a). https://comtrade.un.org/

UNSD: Industrial Commodity Statistics Database 1950-2008 [Computer software]. (2019b). https://unstats.un.org/unsd/industry/Commodity/

UNSD. (2020). *Energy Statistics*. https://unstats.un.org/unsd/energystats/data/

USDT. (1993). *A study of the use of recycled paving material: Report to Congress*. http://www.asphaltrubber.org/ARTIC/Reports/RPA_A1025.pdf

USGS. (2014). *Forestry statistics, ", in Kelly, T.D., and Matos, G.R., comps., Historical statistics for mineral and material commodities in the United States: U.S. Geological Survey Data Series 140*. http://minerals.usgs.gov/minerals/pubs/historical-statistics/

USGS. (2018). *Mineral Commodity Summaries 2018*. https://minerals.usgs.gov/minerals/pubs/mcs/2018/mcs2018.pdf

USGS. (2019). *Mineral Commodity Summaries*. https://minerals.usgs.gov/minerals/pubs/mcs/

van Eygen, E., Feketitsch, J., Laner, D., Rechberger, H., & Fellner, J. (2017). Comprehensive analysis and quantification of national plastic flows: The case of Austria. *Resources, Conservation and Recycling*, *117*, 183–194. https://doi.org/10.1016/j.resconrec.2016.10.017

Wang, M., Chen, W., & Li, X. (2015). Substance flow analysis of copper in production stage in the U.S. from 1974 to 2012. *Resources, Conservation and Recycling*, *105*, 36–48. https://doi.org/10.1016/j.resconrec.2015.10.012

Wang, T., Müller, D. B., & Graedel, T. E. (2007). Forging the anthropogenic iron cycle. *Environmental Science & Technology*, *41*(14), 5120–5129. https://doi.org/10.1021/es062761t

Wernick, I., Herman, R., Govind, S., & Ausubel, J. (1996). Materialization and dematerialization: Measures and trends. *Daedalus*, *125*(3), 171–198.

Wiedenhofer, D., Fishman, T., Lauk, C., Haas, W., & Krausmann, F. (2019). Integrating Material Stock Dynamics Into Economy-Wide Material Flow Accounting: Concepts, Modelling, and Global Application for 1900–2050. *Ecological Economics*, *156*, 121–133. https://doi.org/10.1016/j.ecolecon.2018.09.010

Wiedenhofer, D., Plank, B., Fishman, T., Miatto, A., Haas, W., Haberl, H., & Krausmann, F. (2021). Towards a saturation of the socio-economic metabolism? Maintenance and expansion of growing material stocks in 9 world-regions from 1900-2035: Ín preparation.

Wilburn, D., & Goonan, T. (1998). *Aggregates from Natural and Recycled Sources: Economic Assessments for Construction Applications—A Materials Flow Analysis*. https://pubs.usgs.gov/circ/1998/c1176/c1176.pdf

World Steel Association. (2019). *Steel Statistical Yearbook 1978-2018*. https://www.worldsteel.org/steel-by-topic/statistics/steel-statistical-yearbook.html

Zeltner, C., Bader, H.-P., Scheidegger, R., & Baccini, P. (1999). Sustainable metal management exemplified by copper in the USA. *Regional Environmental Change*, *1*(1), 31–46. https://doi.org/10.1007/s101130050006
